# Structural and Interfacial Characterization of a Photocatalytic Titanium MOF-Phosphate Glass Composite

**DOI:** 10.1021/acsami.4c18444

**Published:** 2025-03-04

**Authors:** Celia Castillo-Blas, Montaña J. García, Ashleigh M. Chester, Matjaž Mazaj, Shaoliang Guan, Georgina P. Robertson, Ayano Kono, James M. A. Steele, Luis León-Alcaide, Bruno Poletto-Rodrigues, Philip A. Chater, Silvia Cabrera, Andraž Krajnc, Lothar Wondraczek, David A. Keen, Jose Alemán, Thomas D. Bennett

**Affiliations:** † Department of Materials Science and Metallurgy, University of Cambridge, Cambridge CB3 0FS, United Kingdom; ‡ Organic Chemistry Department, Science Faculty, Universidad Autónoma de Madrid, C/Francisco Tomás y Valiente, 7, 28049 Madrid, Spain; § Institute of Chemistry, Hajdrihova 19, SI-1000 Ljubljana, Slovenia; ∥ Maxwell Centre, Cavendish Laboratory, University of Cambridge, J. J. Thomson Avenue, Cambridge CB3 0HE, United Kingdom; ⊥ Diamond Light Source Ltd., Diamond House, Harwell Campus, Didcot, Oxfordshire OX11 0QX, United Kingdom; # Department of Chemistry Yusuf Hamied, University of Cambridge, Lensfield Road, Cambridge CB2 1EW, United Kingdom; ∇ Instituto de Ciencia Molecular (ICMol), Universidad de Valencia, c/Catedrático José Beltrán 2, Paterna 46980, Spain; ○ Otto-Schott Institute of Materials Research, University of Jena, Fraunhoferstrasse 6, 07743 Jena, Germany; ◆ Inorganic Chemistry Department, Science Faculty, Universidad Autónoma de Madrid, C/Francisco Tomás y Valiente, 7, 28049 Madrid, Spain; ¶ Institute for Advanced Research in Chemical Sciences (IAdChem), Universidad Autónoma de Madrid, C/ Francisco Tomás y Valiente, 7, 28049 Madrid, Spain; †† ISIS Facility, Rutherford Appleton Laboratory, Harwell Campus, Didcot, Oxfordshire OX11 0QX, United Kingdom

**Keywords:** MOF composite, pair distribution function, photocatalysis, materials characterization, interfacial analysis

## Abstract

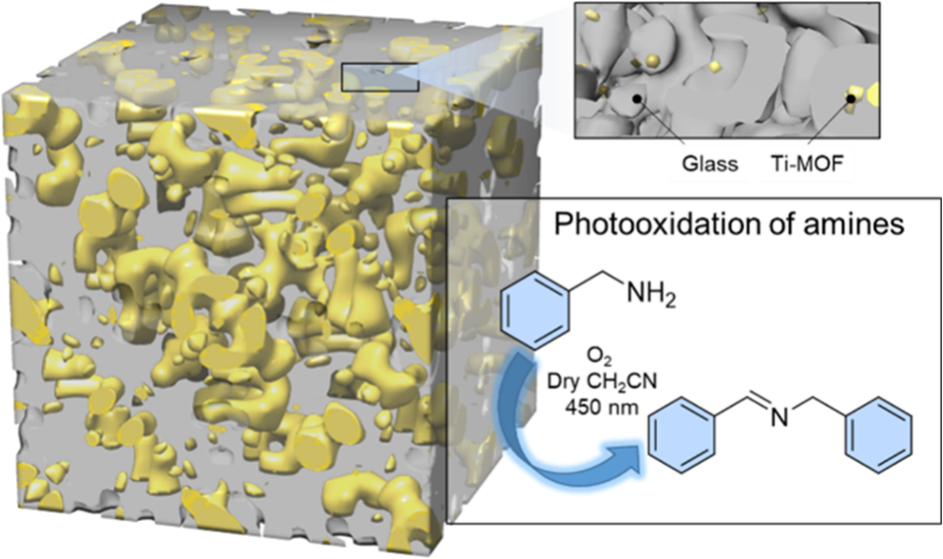

Metal–organic framework (MOF) composites are proposed as solutions to the mechanical instability of pure MOF materials. Here, we present a new compositional series of recently discovered MOF–crystalline inorganic glass composites. In this case, formed by the combination of a photocatalytic titanium MOF (MIL-125-NH_2_) and a phosphate-based glass (20%Na_2_O–10%Na_2_SO_4_–70%P_2_O_5_). This new family of composites has been synthesized and characterized using powder X-ray diffraction, thermal gravimetric analysis, differential scanning calorimetry, scanning electron microscopy, and X-ray total scattering. Through analysis of the pair distribution function extracted from X-ray total scattering data, the atom–atom interactions at the MOF–glass interface are described. Nitrogen and carbon dioxide isotherms demonstrate good surface area values despite the pelletization and mixing of the MOF with a dense inorganic glass. The catalytic activity of these materials was investigated in the photooxidation of amines to imines, showing the retention of the photocatalytic effectiveness of the parent pristine MOF.

## Introduction

The metal–organic framework (MOF) materials family is one of the biggest porous materials families that has been explored by the scientific community in the last 25 years.^1^ They consist of the self-assembly of organic linkers with inorganic clusters, also called secondary building units (SBUs), resulting in porous structures with different topologies.^2^ However, MOFs are typically obtained as microcrystalline powders exhibiting poor mechanical properties, which presents a challenge to industrial implementation.^3^ For example, many lose their desirable physical properties such as porosity during processing, the retention of which is crucial for many applications including water harvesting,^4^ gas sorption,^5^ drug delivery,^6^ and heterogeneous catalysis.^7^

MOF composites have recently received great attention as they offer a realistic solution to maintain MOF properties while mitigating mechanical instability and processing issues.^8^ MOF composites are typically formed by combining the crystalline MOF with another porous or nonporous material. There are many kinds of composites, including mixed-matrix membranes,^9^ silica–MOF composites,^10^ MOFs coated with metal oxides (MO)^11^ and graphene oxide (GOx) containing MOFs.^12^ These kinds of composites are designed to enhance properties like electrocatalytic performance (GOx–MOF composites),^13^ gas sensing (CuO nanoclusters in MOF-808),^14^ water remediation (Fe_3_O_4_@MIL-100)^15^ or photocatalysis (TiO_2_@ZIF-8).^16^ A common issue with compositing, however, is a reduction in the chemical and physical efficacy of the resultant material. In addition, composites, in general, are highly sensitive to the nature of the interface.

A new family of MOF composites has recently been reported in which MOF crystallites are embedded in an inorganic glass matrix.^17−19^ Glasses are amorphous materials that upon heating, above the glass transition temperature (*T*_g_), exhibit a phase transition from a brittle solid to a more viscoelastic material.^20^ The fabrication of MOF–glass composites involves the preparation of a physical mixture of both materials, followed by pelletization and a thermal treatment at a temperature over the *T*_g_ of the glass.^21^ However, extending this procedure to other MOF–inorganic glass combinations is extremely challenging due to the relatively low decomposition temperatures (*T*_d_s) of MOFs compared to that needed for the thermal treatment.^22−24^ Moreover, the MOF pores might be blocked due to the dense matrix used, and this might impact their chemical and physical properties, reducing their efficacy in multiple applications such as gas sorption or catalysis.

Here we present a new family of MOF-crystalline inorganic glass composites (MOF–CIGCs) combining a Ti–MOF (MIL-125-NH_2_) and phosphate-based glass (70%P_2_O_5_–20%Na_2_O–10%Na_2_SO_4_). The atomic interfacial interactions between phases were unveiled by X-ray total scattering analysis using the pair distribution function (PDF) and the photocatalytic activity of this materials family was evaluated and compared with the pristine MOF.

## Results and Discussion

### Fabrication and Characterization of the Composite

**Figure 1:**
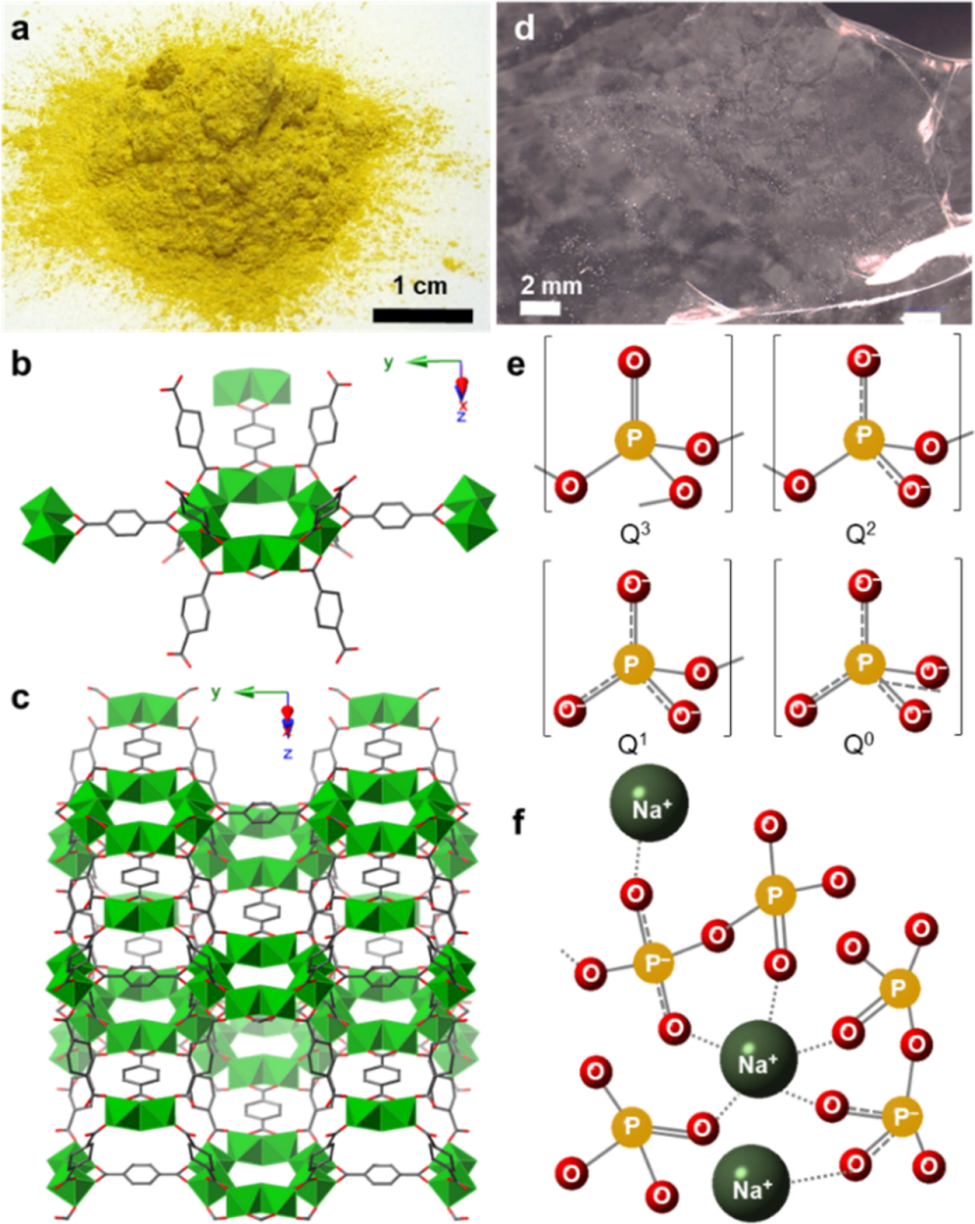
(a) Optical image of pristine as-synthesized Ti–MOF (MIL-125-NH_2_). (b) Secondary building unit of the Ti–MOF and coordinated linkers depiction. (c) 3D Ti–MOF structure. Ti octahedra are shown in green, and C and O atoms are depicted in gray and red, respectively. N atoms from the amino group and H atoms were omitted for the sake of clarity. (d) Optical image of a 70%P_2_O_5_–20%Na_2_O–10%Na_2_SO_4_ glass piece. (e) Depictions of the PO_4_ tetrahedral units that make up phosphate glass structures. *Q*^3^, *Q*^2^, *Q*^1^, and *Q*^0^ correspond to phosphorus pentoxide, meta-, pyro-, and ortho-phosphate, respectively. (f) Schematic depiction of a sodium phosphate glass structure.

Ti–MOF (MIL-125-NH_2_, Ti_8_O_36_H_34_N_6_C_48_) and the inorganic glass (IG, 70%P_2_O_5_–20%Na_2_O–10%Na_2_SO_4_) were synthesized following a slight modification to the reported procedures.^25,26^ Ti–MOF was obtained as a yellow, microcrystalline powder (Figure 1a). Its structure consists of a Ti_8_O_8_(OH)_4_ SBU linked through 2-aminoterephthalate (NH_2_BDC) ligands (Figure 1b), forming a porous structure with *fcu* topology (Figure 1c). The MOF structure was confirmed by Pawley refinement of powder X-ray diffraction (PXRD) data (Figure S1 and Table S1). The MOF particles’ size and shape were analyzed by scanning electron microscopy (SEM) (Figure S2), and the formula (Ti_8_O_8_(OH)_4_(NH_2_BDC)_6_) containing a small number of solvent molecules was confirmed by CHN analysis (Table S2). The *T*_d_ (380 °C) was obtained from thermogravimetric analysis (TGA) of the activated Ti–MOF (Figure S3).

70%P_2_O_5_–20%Na_2_O–10%Na_2_SO_4_ glass was obtained as a transparent glass chunk (Figure 1d). It was ball milled at 30 Hz for 30 min and stored in dry acetone (Figure S4). These phosphate-based glasses are composed of PO_4_ tetrahedral groups classified using the *Q*^*n*^ terminology, where *n* is the number of bridging oxygen atoms per polyhedral (Figure 1e,f).^27^ The amorphous nature of the IG was confirmed by PXRD, which displayed a diffraction pattern typical of phosphate-based glasses (Figure S5).^27^ Differential scanning calorimetry (DSC) of the IG showed a *T*_g_ at 178 °C (Figure S6), lower than the MOF *T*_d_ and thus indicating that this material is thermally compatible with the Ti–MOF to form a composite. *T*_g_ is the minimum temperature necessary to form the more viscoelastic phase of the IG.^21^

**Figure 2:**
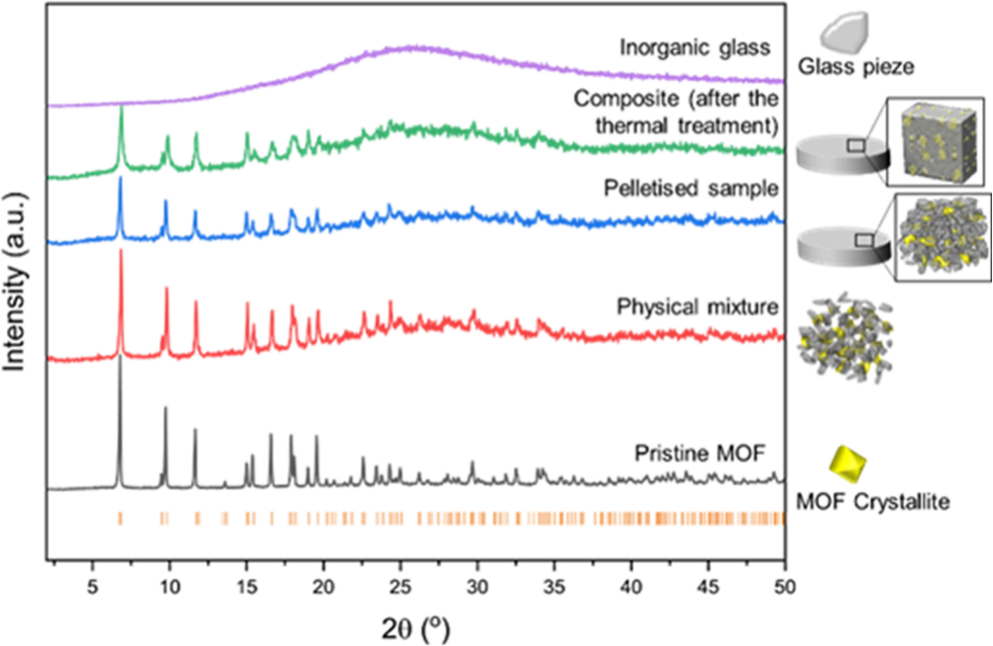
(Left) PXRD of the glass (violet), pristine MOF (gray), and the physical mixture, sample after pelletization and sample after the thermal treatment of the composite containing 50% by weight of Ti–MOF. The Bragg positions of the Ti–MOF are depicted as orange tick marks. (Right) schematic depiction of each stage of the composite preparation.

The samples from the compositional series of composites [(Ti–MOF)*_x_*(IG)_1–*x*_] were prepared by using different weight percentages of the two constituents. They were synthesized following a slightly modified reported procedure that consisted of ball milling both materials at 20 Hz for 5 min to form a physical mixture ((Ti–MOF)*_x_*/(IG)_1–*x*_).^18,28^ This was then pelletized at 0.074 GPa, and the pellets were heated under vacuum at 180 °C for 30 min.^18^ These pressure and temperature parameters were optimized to obtain cohesive pellets by utilizing the flow of the IG while maintaining the Ti–MOF structure and avoiding any partial decomposition. Sample weight percentages of Ti–MOF were 15, 25, 50, and 75%, which resulted in the [(Ti–MOF)_0.15_(IG)_0.85_], [(Ti–MOF)_0.25_(IG)_0.75_], [(Ti–MOF)_0.50_(IG)_0.50_] and [(Ti–MOF)_0.75_(IG)_0.25_] composites, respectively. Every synthetic step of these materials was monitored by PXRD (Figures 2, S7 and S8); higher amounts of Ti–MOF in the mixture resulted in higher intensities of the Bragg peaks from the Ti–MOF structure. After pelletization, the Bragg peak intensities decrease but are then maintained after thermal treatment for compositions containing 75, 50, 25, and 15% of Ti–MOF loads (Figure S9–S12 and Table S3). CHN analyses of the pristine MOF and the composites were carried out to compare to the calculated one to check their formulas (Table S4). FTIR spectroscopy was also performed to check the vibrational modes of both components (Figure S14). However, due to the complexity and insufficient resolution of the composites spectra, the identification of potential contributions at the interface or modifications to the MOF or glass structure is not feasible.

**Figure 3:**
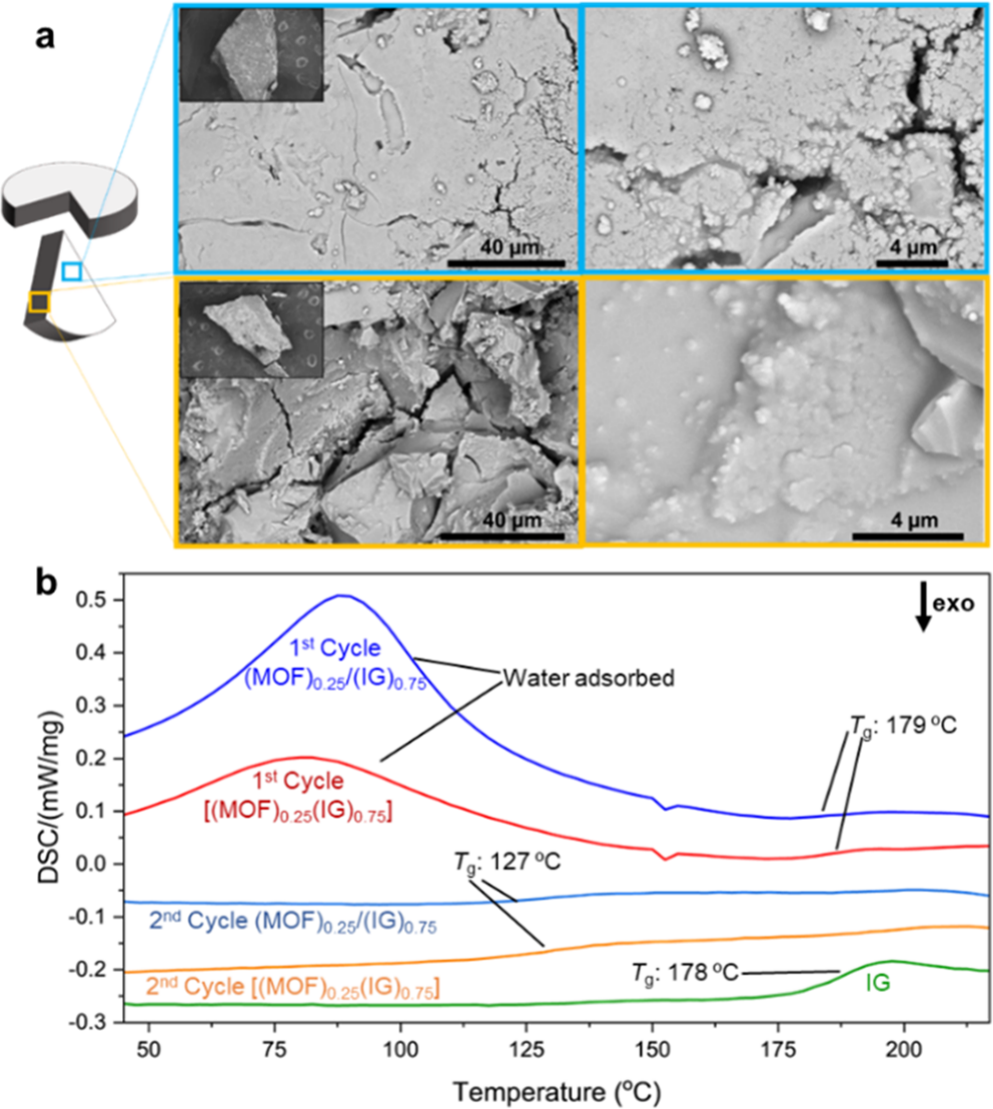
(a) Schematic of a pellet piece of the [(Ti–MOF)_0.50_(IG)_0.50_] composite (left) where SEM images with cyan edges (upper) were taken from the surface of the pellet. Yellow–edged images from inside the pellet show MOF crystallites embedded in the inorganic glass matrix. The insets show the entire pellet pieces, which are 1 mm in size. (b) DSC upscans of the composite [(Ti–MOF)_0.25_(IG)_0.75_], its correspondent physical mixture (Ti–MOF)_0.25_/(IG)_0.75_, and the inorganic glass (IG). First upscans of the physical mixture and the composite release water, likely adsorbed at the pellet surface. Composite *T*_g_s in the first upscan are similar to the pristine glass *T*_g_. However, in the second upscan, they have lower values due to the water partially disrupting the glass network.

SEM images at the surface and within the bulk of the composites revealed a smooth surface interspersed with some small microcracks (Figures S15–S18). The number of cracks is noticeably higher in the [(Ti–MOF)_0.50_(IG)_0.50_] and [(Ti–MOF)_0.75_(IG)_0.25_] composites due to the agglomeration of the MOF crystallites into domains due to the different hydrophobicity properties of the glass and the MOF, and the inability of the glass to form a homogeneous mixture at the surface. MOF agglomerates are formed during ball milling, which persist through pelletization and thermal treatment. Once the glass transition temperature is exceeded, the softened glass embeds the MOF particles, reducing domain visibility in the bulk but leaving them prominent at the surface, as analyzed by SEM and EDX. These surface domains often cause small cracks, likely to minimize repulsive forces between the hydrophilic glass and hydrophobic MOF, and were further characterized using EDX (Figures S23–S30). However, bulk analysis in all cases showed that the glass matrix was able to embed even high loadings of MOF (Figure 3a). This indicates a homogeneous composite formation (Figures S15 and S16). Energy dispersive X-ray (EDX) analyses were carried out at five different composite areas at the surface and within the bulk to determine the percentage of P and Ti and showed homogeneous distributions of both elements (Figures S19–S22). Mapping demonstrated different size domains at the surface (Figures S23–S26) and the bulk (Figures S27–S30), and minor cracks were visible between domains.

The TGA of the composites (Figure S31) shows that there are no major weight losses before 300 °C, confirming the absence of partial decomposition of the MOF components. DSC analyses show a broad feature between 50 and 150 °C, ascribed to water desorption at the first upscan (Figures 3b and S32–S39). This occurs because the phosphate-based glasses are highly hygroscopic, especially when they contain more than 50% of P_2_O_5_.^27,29^ However, the second upscans of composites and physical mixtures containing 15 and 25% of Ti–MOF show a *T*_g_ feature occurring at ∼130 °C. This is far below the pristine glass *T*_g_ (178 °C) (Figures S32–S34). This likely occurs due to the partial depolymerization of the glass network with the presence of the water upon heating.^29^ DSCs from the composite and the physical mixture containing 50% of the Ti–MOF show in the first upscans a smaller broad peak associated with the presence of water and at the second two processes at 130 and 178 °C (Figures S36 and S37). This reinforces the hypothesis that the presence of water disrupts the glass network during heating. However, a *T*_g_ was not identified in the second upscans of the physical mixture and composite containing 75% Ti–MOF, likely due to the extremely small amount of glass in the mixture (Figures S38 and S39).

### Atomic Interfacial Bonding

**Figure 4:**
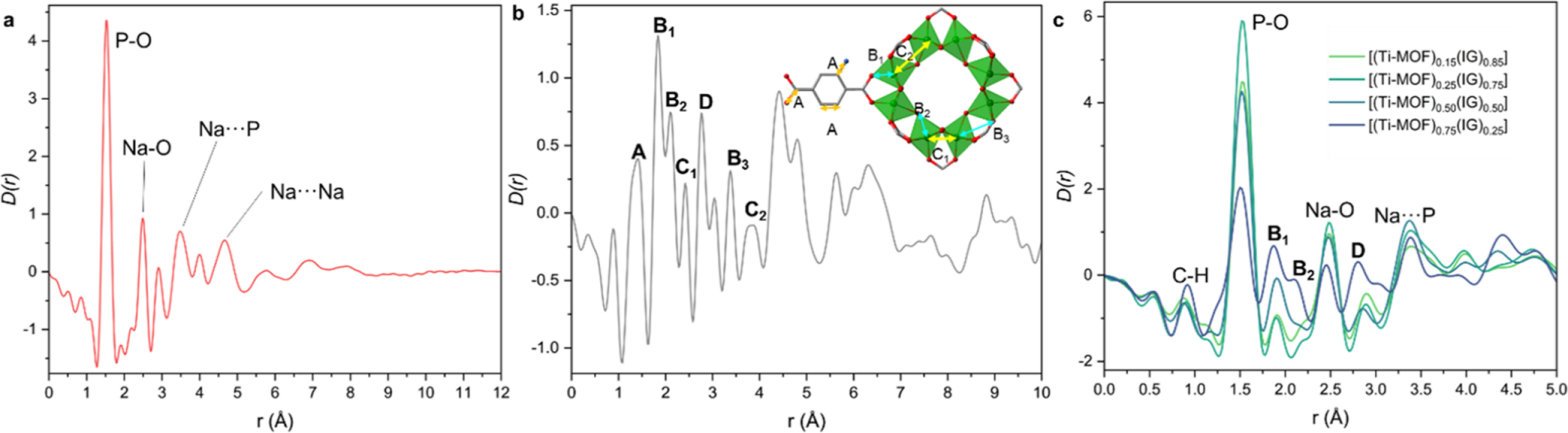
(a) *D*(*r*) of the inorganic glass showing main correlations labeled. (b) *D*(*r*) of the pristine Ti–MOF indicating main correlations labeled. (c) *D*(*r*) of the composites indicating main correlations labeled.

To better understand the structure of this material, and the behavior inferred from the DSC curves, synchrotron X-ray total scattering measurements were collected using kapton capillaries to minimize the background at the I15–1 beamline at the Diamond Light Source facility (U.K.) (Figures S40 and S41). Data were processed to account for adsorption, background, and various scattering corrections using the GudrunX software to extract total scattering structure factors, *S*(*Q*), of all the materials.^30−32^ The *S(Q)* of the IG was consistent with the observed amorphous PXRD pattern (Figure S42). Composites and Ti–MOF materials exhibited the expected Bragg peaks in their *S*(*Q*) (Figures S43 and S44). Pair distribution functions (PDFs) were generated by Fourier transform of the *S*(*Q*) data using the *D*(*r*) function to accentuate high *r* correlations.^32,33^ PDF analysis has been demonstrated to be a powerful technique for describing interatomic distances in MOFs, providing crucial insight into their local structure.^34^ Total and partial PDFs from the Ti–MOF crystal structure from crystal structures related to the IG were calculated using PDFGUI,^35^ identifying the major contributions of the experimental Ti–MOF and IG *D*(*r*) functions (Figures S45–S48). As expected, the PDF of the IG contained peaks at 1.54, 2.47, and 3.43 Å associated with P–O, Na–O, and Na–O–P correlations, similar to the ones found in previous works (Figure 4a).^36^ The Ti–MOF PDF pattern (Figure 4b) contains correlations similar to those in the calculated partial PDFs.

The *D*(*r*) functions of the composites and their corresponding physical mixtures have the same main correlations as their constituents (Figure 4c), which confirms the structural integrity of both the Ti–MOF and IG component materials (Figures S49 and S50). The peak areas vary depending on the proportion of Ti–MOF and glass in the composite. C–H and Ti–O correlations located at 0.9, 1.98 (B_1_), 2.1 Å (B_2_), respectively, are related to the Ti–MOF. P–O (1.53 Å) and P···Na (3.46 Å) correlations increase in area when the proportion of glass in the composite is higher. Other features related to atomic correlations from the Ti–MOF and the inorganic glass overlap, which makes their identification more complex.

Principal component analysis (PCA) and multilinear regression analysis (MLR) of PDF data have recently been demonstrated to be effective approaches to analyze components of MOF composite series as well as describing atom–atom correlations at their interface.^18,19^ PCA is a multivariate analysis that can reduce complex data into its components. Extracted principal components (PCs) may correspond to atomic correlations, atomic distortions, mathematical compensations or noise within the data, and therefore, they are not always chemically intuitive given their purely mathematical derivation.^37^ PCA can be employed to study highly disordered MOF materials, including glasses and amorphous MOFs as well as composites. Here, three PCs (Figure S51a) were extracted from PCA of all the *D*(*r*)*s* of the materials, i.e. *x* = 0 wt % (pristine glass), 15, 25, 50 and 75 wt % composites [(Ti–MOF)*_x_*(IG)_1–*x*_], and 100 wt % (pristine Ti–MOF).

Most of the peaks in PC1 (72.68%) correspond to the peaks in the glass PDF and its weighting decreases as the amount of Ti–MOF in the mixture increases (Figures S52 and S51b). PC2 (24.93%), in contrast, exhibits the same features as the pristine Ti–MOF PDF with its weighting increasing as the amount of Ti–MOF in the mixture increases (Figures S53 and S51b). The equivalence between PC1 and the IG *D*(*r*) and between PC2 and the Ti–MOF *D*(*r*) is not exact though, as seen by the longer-ranged features in PC1 that are absent in the glass *D*(*r*) but are reminiscent of the Ti–MOF *D*(*r*) in this *r*-range (Figures S52 and S53). This also explains why the weightings in (Figure S51b) are sometimes negative and why the end members of the series (pristine Ti–MOF and IG) are not composed of pure PC1 or PC2.

The weightings of the PC3 function, which accounts for 1.29% of the PCA, are positive for 0, 15, and 100 wt % samples and negative for the other composites. PC3 is a repository for parts of the PDFs that are not fully accounted for by PC1 and PC2. These can be additional real interactions in the composites, as well as indicators of deficiencies in the overall analysis method. Taking the latter first, PC3 includes correlations at higher *r*-values that mimic the PDF from Ti–MOF in this *r*-region (Figure S55). The weightings in (Figure S52b) show that PC3 acts to diminish the contribution from PC2 (∼Ti–MOF *D*(*r*)) for the 0 and 15 wt % samples’ PDFs while increasing the Ti–MOF *D*(*r*) content in the 100 wt % sample’s *D*(*r*). It acts in a similar, but less obvious, manner to increase the glass-like features present at low-*r* in PC1 for the *D*(*r*) from 0 and 15 wt % samples. Of more interest is whether PC3 contains features not associated with either Ti–MOF or IG *D*(*r*). The two main *negative* peaks in PC3 (Figure S54a,b, note that the PC3 weighting is negative for all but one of the composite samples) are located at 1.90 and 3.25 Å and might be related to the Ti–O shorter distance because of the thermal treatment and a Ti–O···P correlation likely related to an interaction at the interface (Figure S55).

Multilinear regression (MLR) analysis has also been used to obtain atom–atom correlations at the interface. MLR consists of using end member (Ti–MOF and IG, in this case) *D*(*r*) functions to fit composite *D*(*r*) functions of the compositional series according to eq 1.
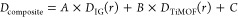
1

**Figure 5:**
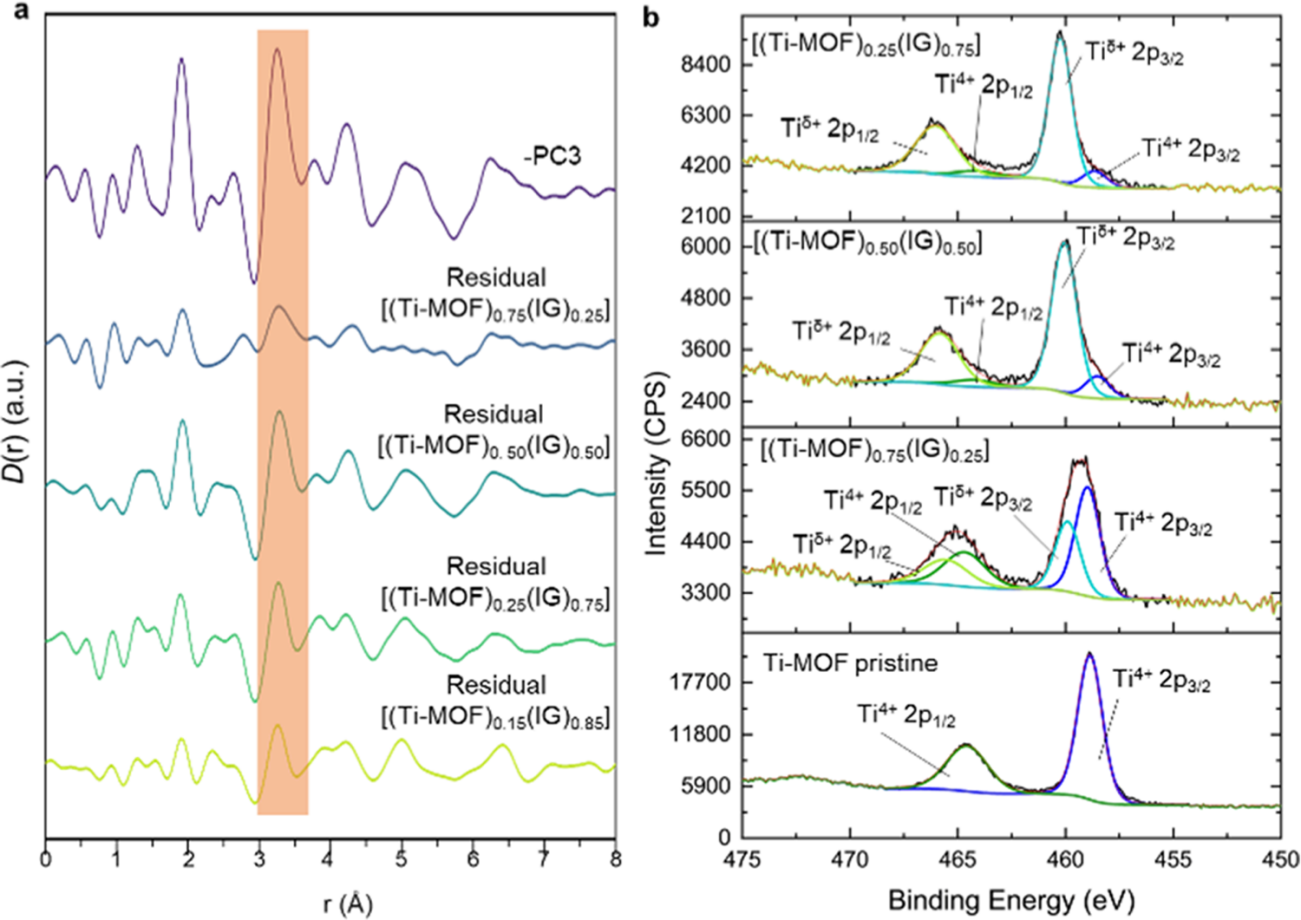
(a) Comparison between residuals of the multilinear regression analysis and negative PC3, which exhibit similar features. Correlation located at 3.25 Å is highlighted in orange. (b) Ti2p XPS spectra including the deconvolution peaks (Ti2p_1/2_ and Ti2p_3/2_) for the pristine Ti–MOF and the composites show a shift to higher binding energies when the proportion in the composite of the IG increases.

The residual obtained from subtracting the calculated composite *D*(*r*) from the experimental *D*(*r*) will contain any contributions from interactions at composite interfaces.^18,19^ The calculated composite *D*(*r*)s fit to the experimental *D*(*r*)s well, evidenced by good correlation coefficients and maintaining a *C* value equal to zero (Figures S56 and Table S5). Their residuals have very similar features, albeit with different scales, to those of -PC3 (Figure 5). This further indicates that these features might be related to the Ti···O···P correlation from the Ti–MOF–glass interface. A similar behavior was observed in other MOF–IG composites containing ZIF-8 or ZIF-62, where Zn···O···P have been observed from PDF data analysis.^18,19^ These correlations also satisfy the charge balance.^17^ Thanks to the use of kapton capillaries during the collection, correlations from the capillary were minimized, and this raises the probability of identifying potential correlations at the interface and the potential distortions of the coordination environments during composite fabrication.

To check whether the titanium cation maintains its oxidation state after the formation of the composite, X-ray photoelectron spectroscopy (XPS) experiments were carried out for the pristine MOF and the [(Ti–MOF)_0.75_(IG)_0.25_], [(Ti–MOF)_0.50_(IG)_0.50_] and [(Ti–MOF)_0.25_(IG)_0.75_] composites. [(Ti–MOF)_0.15_(IG)_0.85_] was not studied because of the presence of Bragg peaks in the PXRD and X-ray total scattering experiments. Survey scans revealed the presence of O, Ti, C, and N in all the samples and, in addition, P and Na for all the composites (Figures S57–S60).

High-resolution XPS spectra of the C1s, O1s, and Ti2p regions were analyzed with the corresponding deconvolution (Figures S57–S60 and Tables S6–S8). The C 1s spectra showed four potential contributions, according to the literature,^38,39^ corresponding to C–O (287.04 eV), C–N (286.25 eV), C–C (284.8 eV) and C=O (288.71 eV) for the pristine Ti–MOF. These contributions varied slightly with the incorporation of the inorganic glass into the mixture in the composites (Table S6). The O 1s profile showed contributions from C–O/O–H groups (531.92 eV) and Ti–O (530.24 eV) for the pristine material. The Ti–O contribution was drastically minimized in the composites, and the C–O/O–H and P–O (∼533 eV) gained a great influence with increasing ratios of the inorganic glass in the composites (Table S7). The Ti2p spectra from all the materials exhibited two peaks corresponding to Ti2p_1/2_ and Ti2p_3/2_ with 458.86 and 464.56 eV values from the pristine Ti–MOF, respectively. These values shifted to higher binding energies with increasing content of the inorganic glass (Figure 5b), indicating the formation of a bond or interaction with a more electronegative species, such as a phosphate group. The deconvolution of the Ti2p_3/2_ and Ti2p_1/2_ peaks revealed two distinct contributions, corresponding to titanium in different oxidation states, Ti^4+^ and Ti^δ+^ (Figure 5b),^40^ likely due to coordination with phosphate groups. This also evidenced the potential P···O···Ti interactions that were unveiled by using the PDF analysis.

### Sorption Properties

**Figure 6:**
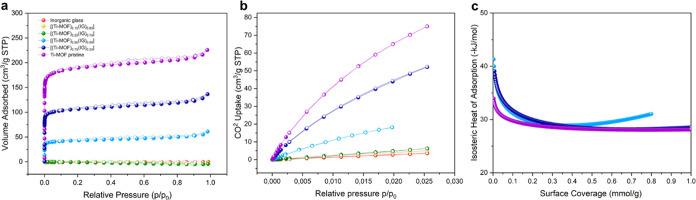
(a) N_2_ adsorption/desorption isotherms at 77 K show the increasing surface area upon Ti–MOF weight ratio incorporation in the composites. (b) CO_2_ adsorption/desorption isotherms were collected at 273 K. (c) Isosteric heat of adsorption was observed for the composite series and the pristine Ti–MOF and inorganic glass materials. Legend showed in (a) is applicable also to the (b, c) isotherms.

Gas sorption measurements were performed to determine the pore availability of the material, which is an essential property of many MOF applications. This is especially crucial when a dense matrix, such as inorganic glass, is employed in MOF composites. According to N_2_ sorption measurements, composites containing higher loads of Ti–MOF produce good results with 327, 618 and 821 m^2^/g Brunauer–Emmett–Teller (BET) values for [(Ti–MOF)_0.50_(IG)_0.50_], [(Ti–MOF)_0.75_(IG)_0.25_] and pristine Ti–MOF (pelletized at 0.74 GPa for comparison with the composites), respectively (Table S9 and Figure 6a). The BET surface area of the pristine Ti–MOF is substantially smaller than the reported one;^41^ this might be due to a partial structural collapse during pelletization (Figure S13). Carbon dioxide isotherms at 273 and 283 K were also collected, showing a higher CO_2_ uptake for composites containing a larger proportion of Ti–MOF (Table S10, Figures 6b and S61). The variation of the ratio MOF/IG vs the gas uptake values is not linear; in fact, the amount of gas adsorbed for the [(Ti–MOF)_0.50_(IG)_0.50_] and [(Ti–MOF)_0.75_(IG)_0.25_] composites is presumably higher than expected (Figure S62). This might be due to the presence of small cracks in the pellet surface between domains that allow better carbon dioxide diffusion.

An isosteric heat of adsorption (Δ*H*_ads_) study was performed based on Clausius–Clapeyron method, calculated from isotherms measured at both temperatures using the Sips model (see SI, Table S11, Figures S63, S64 and 6).^42,43^ The composites with a MOF contribution of up to 25% show stronger binding sites for CO_2_ at low coverages (ca. 45 kJ/mol). With a larger amount of Ti–MOF, the enthalpy trend approximates the values observed for the pure Ti–MOF at ca. 30 kJ/mol. This indicates that the glass component provides strong polar sites that enhance the interactions with CO_2_ on its surface. This might also be related to the presence of the interface glass–MOF containing titanium phosphate at a local scale described in the section above. However, these data are not enough to confirm this synergic effect.

### Photocatalytic Study

Although MOF composite materials usually exhibit improved mechanical properties, they often show decreased chemical and physical properties because the matrix may change the active sites, and consequently, their catalytic activity can be reduced or even lost. Therefore, the assessment of their catalytic activity may help in testing the chemical properties of those materials. More concretely, the photocatalytic activity of this family of materials was evaluated toward the oxidative coupling of amines to afford imines as a model reaction.^44,45^

**Figure 7:**
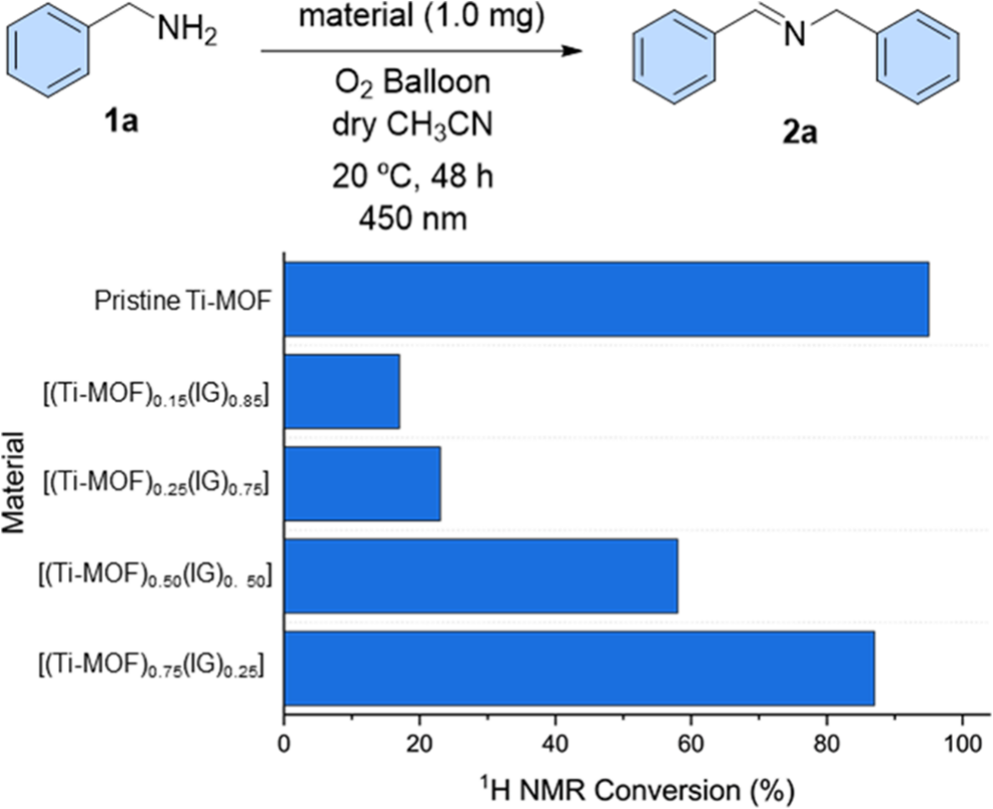
Oxidative coupling of amines into imines as model reaction catalyzed by composites with different Ti–MOF content and pristine Ti–MOF (MIL-125-NH_2_). Measurement error has not been possible to estimate due to the insufficient number of repetitions.

First, the activity of the composites containing 25, 50, and 75% of Ti–MOF loads was evaluated and compared to that of the pristine MIL-125-NH_2_. This study was performed using benzylamine (**1a**) and 1 mg of the corresponding material in dry acetonitrile, and the mixture was stirred under an O_2_ atmosphere while irradiated at 450 nm for 48 h (Figure 7). There is a linear relationship between the Ti–MOF content in the composite and its catalytic activity, and consequently, the most catalytically active material contained 75% of MOF. This material [(Ti–MOF)_0.75_(IG)_0.25_] was able to achieve the corresponding imine in 87% of conversion, which is very close to that of the pristine material (95%). This result confirms the preservation of the catalytic activity of the MOF after the composite formation but with a decrease of 8% due to the partial collapse of the structure after pressure. In order to clarify this point, a fresh batch of the pristine Ti–MOF and a pelletized Ti–MOF were also tested, demonstrating a 6% loss in the photocatalytic activity after pelletization (Table S12). However, this value remains surprisingly high, considering that the presence of a dense and nonphotoactive phosphate-based glass would typically be expected to worsen the catalytic properties. This deviation might be attributed to the small presence of the titanium–phosphate interactions at the interface that have been demonstrated as photoactive.^46^ It is important to mention that the reaction did not occur in the absence of light, oxygen, or material. These results were compared to the pristine Ti–MOF and other Ti–MOF derivatives under different catalytic conditions (Table S13),^47,48^ highlighting the composite’s potential for its application as a photocatalyst particularly due to the smaller amount of material used in this work.

Next, different reaction parameters were optimized using [(Ti–MOF)_0.75_(IG)_0.25_] material. More concretely, the effects of the solvent (Table S14), the amount of the catalytic material (Table S15), and the irradiation source (Figures S66 and S67) were studied. These experiments showed that the best catalytic performance of [(Ti–MOF)_0.75_(IG)_0.25_] was reached using 0.5 mg of the composite, acetonitrile as a solvent, and a 420 nm light-emitting diode (LED) system for irradiating the reaction.

**Figure 8:**
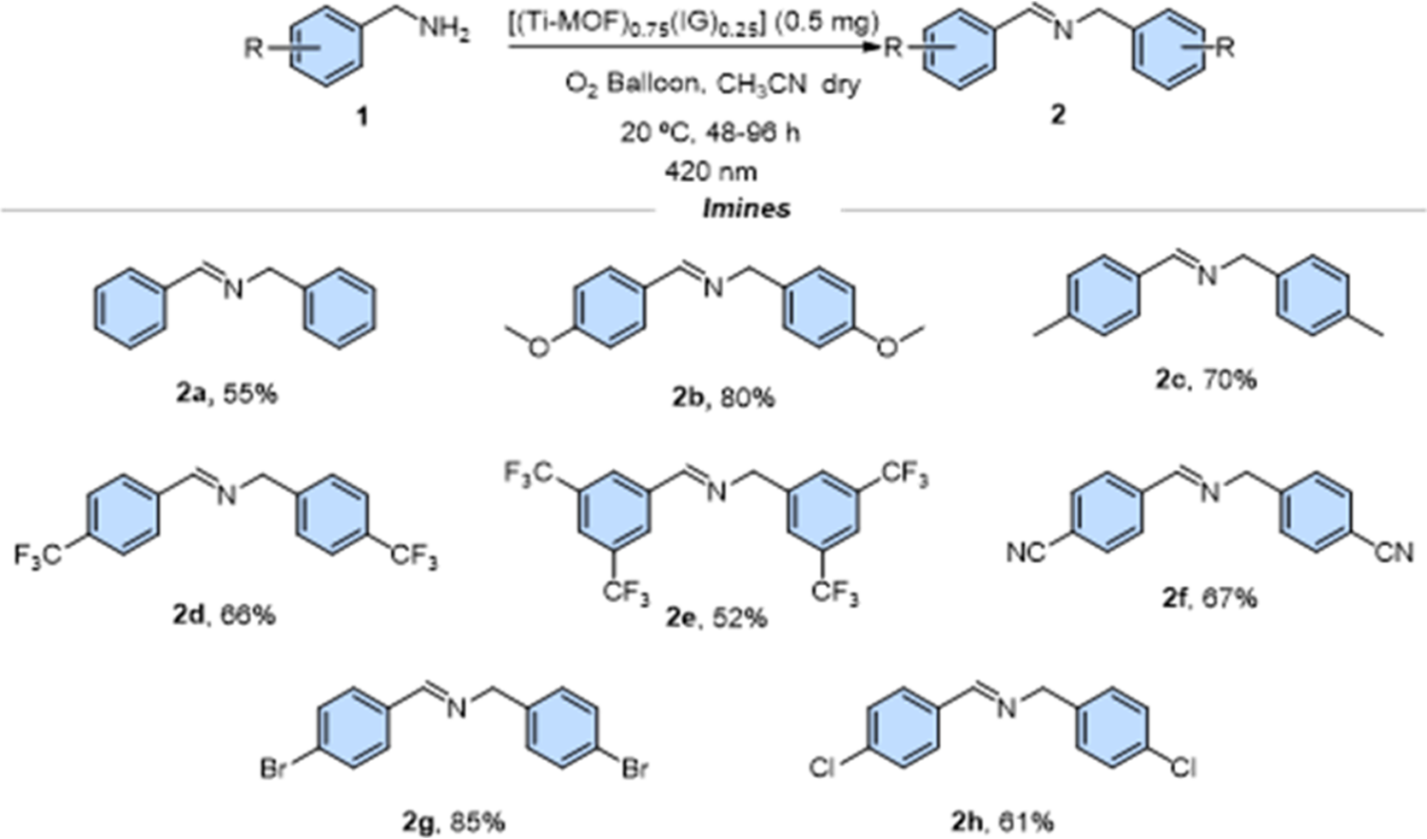
Reaction conditions: The corresponding benzylamine 1 (0.1 mmol) and 0.5 mg of [(Ti–MOF)_0.75_(IG)_0.25_] in 1 mL of dry acetonitrile were irradiated at 420 nm. The yields were determined by ^1^H NMR analysis using 1,3,5-trimethoxybenzene as the quantitative standard.

Composite [(Ti–MOF)_0.75_(IG)_0.25_] was able to catalyze the oxidative coupling of benzyl amines **1** having both electron-donating and electron-withdrawing groups in the aromatic ring, affording the corresponding imines **2** in good yields (52–85%) (Figure 8).

One of the most important factors to assess in heterogeneous catalysis is the recyclability of the catalyst. For this purpose, the oxidative coupling of benzylamine **1a** using [(Ti–MOF)_0.75_(IG)_0.25_] under optimized conditions was stopped after 48 h. The material was separated from the reaction mixture by filtration, and it was washed and dried. Then, the recovered material was used for a second reaction run by adding new benzylamine (**1a**) and solvent, and the reaction was submitted to irradiation. As can be seen in Figure S68, the catalytic activity of the recovered material is maintained, which indicates not only the recyclability of the composite but also its stability under photocatalytic conditions. However, no further runs could be performed due to the small amount of catalyst (0.5 mg). Nevertheless, Ti–MOF has been demonstrated in the literature to have good recyclability for up to three cycles.^47^

### Stability in the Air

Phosphate-based glasses are highly hygroscopic, readily absorbing significant amounts of water on their surface.^49^ This property makes them very unstable in air, particularly when their P_2_O_5_ content exceeds 50%, as in the case of the glass used in this study (70%P_2_O_5_–20%Na_2_O–10% Na_2_SO_4_). Notably, within just two h of manual grinding for 5 min or ball milling at 30 Hz for 30 min in air, the surface of all glass pieces becomes covered with water (Figure S77). This behavior significantly impacts their suitability for composite preparation.

In this work, we investigated the air stability of the composites and pristine materials by exposing them to ambient conditions (room temperature and 66% relative humidity) for 7 days. According to PXRD patterns, the structure of all materials remains intact, though a significant decrease in Bragg intensities is observed for the Ti–MOF (Figure S78). This suggests that the presence of water increases the instability of the MOF.

SEM images of the composites after exposure to air reveal that composites with lower MOF ratios ([(Ti–MOF)_0.15_(IG)_0.85_] and [(Ti–MOF)_0.25_(IG)_0.75_]) show water droplets covering the glass surface domains. In contrast, composites with a higher MOF content maintain water-free glass surfaces (Figure S79). This indicates that the Ti–MOF, even when embedded in a glass matrix, retains its ability to absorb water.^41^

These findings suggest that the Ti–MOF could enhance the potential applications of such composites, for example, in the development of smart devices like self-cleaning windows due to their water stability.

## Conclusions

This work describes the synthesis, characterization, and adsorption and photocatalytic properties of a new family of MOF-inorganic glass composites containing different proportions of a Ti–MOF (MIL-125-NH_2_) and a phosphate glass (20%Na_2_O–10%Na_2_SO_4_–70%P_2_O_5_). Each synthetic step was monitored by PXRD showing that the Ti–MOF maintains its crystallinity within the composite using the optimized synthetic conditions. The composites’ microstructure was characterized by SEM showing a smooth distribution of the MOF crystallites in the bulk of the pellets. EDS showed a homogeneous distribution of P and Ti in ∼ 270 μm^2^ areas but having ∼ 30 μm^2^ domains of each precursor material, according to the mapping analyses. Thermal characterization of these composites has identified a *T*_g_ that is lower than that of the pristine inorganic glass, indicating a partial depolymerization of the glass network upon heating. This is likely because of the presence of water on the composite surface.

PDF analyses of the composites helped to understand their structure, and correlations between the two components were observed. In addition, a recent methodology was employed to decipher the potential interactions at the interface, showing potential Ti···O···P correlations. This hypothesis is also supported by XPS data analysis, where in the Ti2p spectra, Ti2p_1/2_ and Ti2p_3/2_ peaks are shifted to higher binding energy when the proportion of the IG increases in the composite, suggesting the bond or the interaction between the Ti and a more electronegative moiety, such as the phosphate.

Gas sorption analysis was performed by collecting N_2_ and CO_2_ isotherms. BET surface areas were calculated for the compositions containing higher proportions of Ti–MOF in the composite. CO_2_ isotherms also show that composites containing higher MOF loadings have higher gas uptake values. These values are higher than expected based on their molar proportion. Based on the isosteric heat of adsorption (Δ*H*_ads_) values, it is observed that glass provides strong polar sites that demonstrated that with an increment in the Ti–MOF proportion in the composite, Δ*H*_ads_ values are similar to those of the pristine Ti–MOF.

The photocatalytic activity of this family of materials has been tested toward the photooxidation of amines to imines. The catalytic results indicate that a composite with a 75% weight of the Ti–MOF exhibits a photocatalytic activity similar to that of the pristine material, which confirms the preservation of the catalytic activity after the composite formation.

Therefore, this work opens exciting avenues in the use of new MOF composite materials that might be employed, for example, in the fabrication of self-cleaning windows thanks to the use of transparent inorganic glasses as matrices.

## Materials and Methods

### Synthesis of MIL-125-NH_2_ (Ti–MOF)

MIL-125-NH_2_ was obtained starting from 3 mmol of 2-aminoterephthalic (560 mg), 2 mmol of titanium isoproproxide Ti(OiPr)_4_ (0.6 mL), introduced in a solution of 9 mL of *N*,*N*-dimethylformamide (DMF) and 1 mL of dry methanol. The mixture was stirred gently for 5 min at room temperature and then further introduced in a 23 mL Teflon liner and then put into a stainless-steel autoclave and placed into a preheated oven at 150 °C for 15 h. Back to room temperature, the yellow solid was recovered by centrifugation 5 min at 4000 rpm and washed 3 times with 20 mL of fresh DMF and twice with 20 mL of methanol and dried under vacuum at room temperature. The free solvent was removed by calcination at 150 °C overnight for 12 h.

### Synthesis of the Inorganic Glass

The 70%P_2_O_5_–20%Na_2_O–10%Na_2_SO_4_ inorganic glass was prepared by weighing the following precursors in the desired proportions: (NH_4_)_2_HPO_4_, Na_2_CO_3_ and Na_2_SO_4_. Then 50 g batches were melted at 900 °C under air for 1h in alumina crucibles and finally quenched to form homogeneous glassy samples. After quenching each the glass was annealed at 160 °C for 30 min and allowed to cool to room temperature. The glass was ball milled under the nitrogen atmosphere at 30 Hz for 30 min prior to its use for the synthesis of the composites.

### Composite Synthesis

The previously activated Ti–MOF (MIL-125-NH_2_) and the ball-milled inorganic glass (70%P_2_O_5_–20%Na_2_O–10%Na_2_SO_4_) were added in the appropriate weight ratios to a total mass of 200 mg into a 10 mL stainless steel jar. The powders were mixed through ball milling with two 5 mm diameter stainless steel balls for 5 min at 20 Hz in a Retsch MM400 grinder mill. 150 mg samples of the ball-milled powder mixture (physical mixture) were pelletized at 0.074 GPa using 1 ton and 13 mm pellet dye. These pellets were placed in a Thermo Fisher vacuum furnace and heated at different temperature 180 °C and held for 30 min under dynamic vacuum.

### Powder X-ray Diffraction Measurements (PXRD)

Data were collected on a Bruker D8 DAVINCI diffractometer equipped with a position-sensitive LynxEye detector with a Bragg–Brentano parafocusing geometry. Cu Kα1 (λ = 1.5406 Å) radiation was used through a 0.012 mm Ni filter. The samples were compacted into 5 mm disks on a low background silicon substrate and rotated during data collection in the 2θ range of 2–50° at ambient.

### Thermogravimetric Analysis (TGA)

curves were conducted using a TA Instruments Q-650 series. Approximately 5–10 mg of powdered samples were placed in open 90 μL alumina crucibles. The samples were left to equilibrate for 5 min at 30 °C under an argon flow of 100 μL/min before the thermal treatment. A thermal heating using ramp of 10 °C/min was applied between 30 and 800 °C. Data were analyzed using TA Universal Analysis software.

### Optical Microscopy

A Leica MZ95 microscope and a Optika C–B10 camera with a 10 megapixel CMOS sensor was used to obtain optical images of all samples.

### Differential Scanning Calorimetry (DSC)

DSC curves were recorded on a NETSCH DSC 214 Polyma instrument. Approximately 5–10 mg of powdered samples were placed in sealed 70 μL aluminum crucibles. Crucibles were used with a hole punctured in the lid to prevent buildup of pressure. An empty aluminum pan was used as a reference. Background corrections were performed using the same heating cycle on an empty aluminum crucible. All data analysis was performed using the Netzsch Proteus software package.

### CHN Microanalysis

CHN combustion analysis experiments were performed using a CE440 Elemental Analyzer, EAI Exeter Analytical Inc. ∼1.3–1.5 mg of sample was used for each run. Measurements were collected up to 3 times per sample.

### Scanning Electron Microscopy (SEM) and EDS Analysis

SEM images were collected with a high-resolution scanning electron microscope FEI Nova Nano SEM 450, accelerating voltage 15 kV for image acquisition and 20 kV for EDS collection. All samples were prepared by dispersing the material onto a double sided adhesive conductive carbon tape that was attached to a flat aluminum sample holder and were coated with a platinum layer of 15 nm using an Emtech K575 sputter coater.

### X-ray Total Scattering Data

X-ray total scattering data were collected using an X-ray energy of 76.69 KeV (λ = 0.161669 Å) at the I-15–1 beamline, Diamond Light Source, U.K. All powder samples were ground and loaded into kapton capillaries (1 mm inner diameter and 30 mm of length) to heights of 5–8 mm of sample. The capillaries were then sealed with plasticine by both extremes and placed over a 1 mm-diameter stainless steel (25 mm) until it was touching the one of extreme of the plasticine to minimize precession of the capillary while it was spinning. The capillary was fixed to the rod using plasticine, and then the rod was mounted in the standard I15–1 chucks. Total scattering data were collected at room temperature for the background (i.e., empty instrument), empty kapton capillary, and both samples in a *Q* range of 0.35–20.0. Subsequent Fourier transformation of the normalized total scattering data produced in a real space pair distribution function *G*(*r*) for each material. In this work, we use the *D*(*r*) form of the pair distribution function to accentuate high *r* correlations. All processing of the total scattering data was performed using GudrunX following well-documented procedures.^30−32^

### XPS Analysis

XPS analysis was performed using a Thermo NEXSA G2 XPS fitted with a monochromated Al kα X-ray source (1486.7 eV), a spherical sector analyzer, and 3 multichannel resistive plate, 128 channel delay line detectors. All data was recorded at 19.2W and an X-ray beam size of 400 μm × 200 μm. Survey scans were recorded at a pass energy of 200 eV, and high-resolution scans were recorded at a pass energy of 50 eV. Electronic charge neutralization was achieved using an ion source (Thermo Scientific FG-03). Ion gun current = 150 μA. Ion gun voltage = 40 V. All sample data was recorded at a pressure below 10–8 Torr and a room temperature of 294 K. Data was analyzed using CasaXPS v2.3.26rev1.0N. Peaks were fit with a Shirley background prior to component analysis. Lineshapes of LA(1.53,243) were used to fit components.

### Gas Adsorption

Gas adsorption between 100 and 130 mg was degassed by heating under vacuum at 110 °C for two h prior to measurement (vacuum strength 0.1–1 Torr). Carbon dioxide uptake values were recorded using using Autosorb iQ gas adsorption analyzer (Anton Paar) equipped with a temperature-controlled bath at 273 and 283 K. For N_2_ sorption experiments, iQ3 gas adsorption analyzer (Anton Paar) was used at 77 K.

### ^1^H NMR

^1^H NMR spectra were acquired on a BRUKER AVANCE 300 or BRUKER AVANCE-II 300 spectrometer running at 300 MHz for ^1^H and were internally referenced to residual solvent signals (CDCl_3_ referenced at δ 7.26 ppm for ^1^H NMR). Data for ^1^H NMR are reported as follows: chemical shift (δ ppm), multiplicity (s = singlet, d = doublet, t = triplet, q = quartet, m = multiplet, br = broad), coupling constant (Hz), and integration.

### Photoreactor

The reactor used for photocatalytic reactions consists of a custom-made temperature-controlled system, where the reaction mixture was kept at 20.0 °C by passing coolant through the system by employing a recirculating chiller. The vial is placed inside the fitted, located 1 cm below the base of the vial and is irradiated at 420 nm using 380 mW single LEDs (Figure S65).

## Supporting Information Available

Synthesis and characterization details; SEM images; PXRD; synchrotron X-ray total scattering data analysis; gas adsorption isotherms; FTIR, XPS, DSC; photocatalytic studies; and TGAs (PDF)

## References

[ref1] Freund R.; Canossa S.; Cohen S. M.; Yan W.; Deng H.; Guillerm V.; Eddaoudi M.; Madden D. G.; Fairen-Jimenez D.; Lyu H.; Macreadie L. K.; Ji Z.; Zhang Y.; Wang B.; Haase F.; Wöll C.; Zaremba O.; Andreo J.; Wuttke S.; Diercks C. S. 25 Years of Reticular Chemistry. Angew. Chem., Int. Ed. 2021, 60 (45), 23946–23974. 10.1002/anie.202101644. 33783111

[ref2] Furukawa H.; Cordova K. E.; O’Keeffe M.; Yaghi O. M. The Chemistry and Applications of Metal-Organic Frameworks. Science 2013, 341 (6149), 1230444 10.1126/science.1230444. 23990564

[ref3] Ren J.; Langmi H. W.; North B. C.; Mathe M. Review on Processing of Metal–Organic Framework (MOF) Materials towards System Integration for Hydrogen Storage. Int. J. Energy Res. 2015, 39 (5), 607–620. 10.1002/er.3255.

[ref4] Hanikel N.; Prévot M. S.; Yaghi O. M. MOF Water Harvesters. Nat. Nanotechnol. 2020, 15 (5), 348–355. 10.1038/s41565-020-0673-x. 32367078

[ref5] Li J.-R.; Kuppler R. J.; Zhou H.-C. Selective Gas Adsorption and Separation in Metal–Organic Frameworks. Chem. Soc. Rev. 2009, 38 (5), 1477–1504. 10.1039/b802426j. 19384449

[ref6] Horcajada P.; Gref R.; Baati T.; Allan P. K.; Maurin G.; Couvreur P.; Férey G.; Morris R. E.; Serre C. Metal-Organic Frameworks in Biomedicine. Chem. Rev. 2012, 112 (2), 1232–1268. 10.1021/cr200256v. 22168547

[ref7] Lee J.; Farha O. K.; Roberts J.; Scheidt K. A.; Nguyen S. T.; Hupp J. T. Metal–Organic Framework Materials as Catalysts. Chem. Soc. Rev. 2009, 38 (5), 1450–1459. 10.1039/b807080f. 19384447

[ref8] Zhu Q. L.; Xu Q. Metal-Organic Framework Composites. Chem. Soc. Rev. 2014, 43 (16), 5468–5512. 10.1039/C3CS60472A. 24638055

[ref9] Cheng Y.; Datta S. J.; Zhou S.; Jia J.; Shekhah O.; Eddaoudi M. Advances in Metal–Organic Framework-Based Membranes. Chem. Soc. Rev. 2022, 51 (19), 8300–8350. 10.1039/D2CS00031H. 36070414

[ref10] Yuan N.; Zhang X.; Wang L. The Marriage of Metal–Organic Frameworks and Silica Materials for Advanced Applications. Coord. Chem. Rev. 2020, 421, 213442 10.1016/j.ccr.2020.213442.

[ref11] Subudhi S.; Tripathy S. P.; Parida K. Metal Oxide Integrated Metal Organic Frameworks (MO@MOF): Rational Design, Fabrication Strategy, Characterization and Emerging Photocatalytic Applications. Inorg. Chem. Front. 2021, 8 (6), 1619–1636. 10.1039/D0QI01117G.

[ref12] Jayaramulu K.; Mukherjee S.; Morales D. M.; Dubal D. P.; Nanjundan A. K.; Schneemann A.; Masa J.; Kment S.; Schuhmann W.; Otyepka M.; Zbořil R.; Fischer R. A. Graphene-Based Metal–Organic Framework Hybrids for Applications in Catalysis, Environmental, and Energy Technologies. Chem. Rev. 2022, 122 (24), 17241–17338. 10.1021/acs.chemrev.2c00270. 36318747 PMC9801388

[ref13] Jahan M.; Liu Z.; Loh K. P. A Graphene Oxide and Copper-Centered Metal Organic Framework Composite as a Tri-Functional Catalyst for HER, OER, and ORR. Adv. Funct. Mater. 2013, 23 (43), 5363–5372. 10.1002/adfm.201300510.

[ref14] del Castillo-Velilla I.; Sousaraei A.; Romero-Muñiz I.; Castillo-Blas C.; Méndez A. S. J.; Oropeza F. E.; de la Peña O’Shea V. A.; Cabanillas-González J.; Mavrandonakis A.; Platero-Prats A. E. Synergistic Binding Sites in a Metal-Organic Framework for the Optical Sensing of Nitrogen Dioxide. Nat. Commun. 2023, 14 (1), 2506 10.1038/s41467-023-38170-9. 37130858 PMC10154382

[ref15] Zhang C.-F.; Qiu L.-G.; Ke F.; Zhu Y.-J.; Yuan Y.-P.; Xu G.-S.; Jiang X. A Novel Magnetic Recyclable Photocatalyst Based on a Core–Shell Metal–Organic Framework Fe_3_O_4_@MIL-100(Fe) for the Decolorization of Methylene Blue Dye. J. Mater. Chem. A 2013, 1 (45), 14329–14334. 10.1039/c3ta13030d.

[ref16] Zhang M.; Shang Q.; Wan Y.; Cheng Q.; Liao G.; Pan Z. Self-Template Synthesis of Double-Shell TiO2@ZIF-8 Hollow Nanospheres via Sonocrystallization with Enhanced Photocatalytic Activities in Hydrogen Generation. Appl. Catal. B 2019, 241, 149–158. 10.1016/j.apcatb.2018.09.036.

[ref17] Longley L.; Calahoo C.; Limbach R.; Xia Y.; Tuffnell J. M.; Sapnik A. F.; Thorne M. F.; Keeble D. S.; Keen D. A.; Wondraczek L.; Bennett T. D. Metal-Organic Framework and Inorganic Glass Composites. Nat. Commun. 2020, 11, 5800 10.1038/s41467-020-19598-9. 33199681 PMC7669864

[ref18] Castillo-Blas C.; Chester A. M.; Cosquer R. P.; Sapnik A. F.; Corti L.; Sajzew R.; Poletto-Rodrigues B.; Robertson G. P.; Irving D. J. M.; McHugh L. N.; Wondraczek L.; Blanc F.; Keen D. A.; Bennett T. D. Interfacial Bonding between a Crystalline Metal–Organic Framework and an Inorganic Glass. J. Am. Chem. Soc. 2023, 145 (42), 22913–22924. 10.1021/jacs.3c04248. 37819708 PMC10603780

[ref19] Chester A. M.; Castillo-Blas C.; Sajzew R.; Rodrigues B. P.; Mas-Balleste R.; Moya A.; Snelson J. E.; Collins S. M.; Sapnik A. F.; Robertson G. P.; Irving D. J. M.; Wondraczek L.; Keen D. A.; Bennett T. D. Structural Insights into Hybrid Immiscible Blends of Metal–Organic Framework and Sodium Ultraphosphate Glasses. Chem. Sci. 2023, 14 (42), 11737–11748. 10.1039/D3SC02305B. 37920351 PMC10619634

[ref20] Debenedetti P. G.; Stillinger F. H. Supercooled Liquids and the Glass Transition. Nature 2001, 410, 259–267. 10.1038/35065704. 11258381

[ref21] Chester A. M.; Castillo-Blas C.; Wondraczek L.; Keen D. A.; Bennett T. D. Materials Formed by Combining Inorganic Glasses and Metal-Organic Frameworks. Chem. – Eur. J. 2022, 28 (38), e202200345 10.1002/chem.202200345. 35416352 PMC9400909

[ref22] Longley L.; Calahoo C.; Southern T. J. F.; Evans R. C.; Wondraczek L.; Bennett T. D. The Reactivity of an Inorganic Glass Melt with ZIF-8. Dalton Trans. 2021, 50 (10), 3529–3535. 10.1039/D1DT00152C. 33599672

[ref23] Healy C.; Patil K. M.; Wilson B. H.; Hermanspahn L.; Harvey-Reid N. C.; Howard B. I.; Kleinjan C.; Kolien J.; Payet F.; Telfer S. G.; Kruger P. E.; Bennett T. D. The Thermal Stability of Metal-Organic Frameworks. Coord. Chem. Rev. 2020, 419, 213388 10.1016/j.ccr.2020.213388.

[ref24] Castillo-Blas C.; Chester A. M.; Keen D. A.; Bennett T. D. Thermally Activated Structural Phase Transitions and Processes in Metal–Organic Frameworks. Chem. Soc. Rev. 2024, 54, 2606–2629. 10.1039/D3CS01105D. 38426588

[ref25] Dan-Hardi M.; Serre C.; Frot T.; Rozes L.; Maurin G.; Sanchez C.; Férey G. A New Photoactive Crystalline Highly Porous Titanium(IV) Dicarboxylate. J. Am. Chem. Soc. 2009, 131 (31), 10857–10859. 10.1021/ja903726m. 19621926

[ref26] Rodrigues B. P.; Limbach R.; de Souza G. B.; Ebendorff-Heidepriem H.; Wondraczek L. Correlation Between Ionic Mobility and Plastic Flow Events in NaPO_3_-NaCl-Na_2_SO_4_ Glasses. Front. Mater. 2019, 6, 128 10.3389/fmats.2019.00128.

[ref27] Brow R. K. Review: The Structure of Simple Phosphate Glasses. J. Non-Cryst. Solids 2000, 263–264, 1–28. 10.1016/S0022-3093(99)00620-1.

[ref28] Chester A. M.; Castillo-Blas C.; Sajzew R.; Rodrigues B. P.; Lampronti G. I.; Sapnik A. F.; Robertson G. P.; Mazaj M.; Irving D. J. M.; Wondraczek L.; Keen D. A.; Bennett T. D. Loading and Thermal Behaviour of ZIF-8 Metal–Organic Framework-Inorganic Glass Composites. Dalton Trans. 2024, 53 (25), 10655–10665. 10.1039/D4DT00894D. 38860528

[ref29] Brow R. K.; Kirkpatrick R. J.; Turner G. L. The Short Range Structure of Sodium Phosphate Glasses I. MAS NMR Studies. J. Non-Cryst. Solids 1990, 116 (1), 39–45. 10.1016/0022-3093(90)91043-Q.

[ref30] Soper A. K.; Barney E. R. Extracting the Pair Distribution Function from White-Beam X-Ray Total Scattering Data. J. Appl. Crystallogr. 2011, 44 (4), 714–726. 10.1107/S0021889811021455.

[ref31] Soper A. K. GudrunN and GudrunX: Programs for Correcting Raw Neutron and X-Ray Diffraction Data to Differential Scattering Cross Section, Science & Technology Facilities Council 2011.

[ref32] Keen D. A. A Comparison of Various Commonly Used Correlation Functions for Describing Total Scattering. J. Appl. Crystallogr. 2001, 34 (2), 172–177. 10.1107/S0021889800019993.

[ref33] Soper A. K. The Radial Distribution Functions of Water and Ice from 220 to 673 K and at Pressures up to 400 MPa. Chem. Phys. 2000, 258 (2), 121–137. 10.1016/S0301-0104(00)00179-8.

[ref34] Castillo-Blas C.; Moreno J. M.; Romero-Muñiz I.; Platero-Prats A. E. Applications of Pair Distribution Function Analyses to the Emerging Field of Non-Ideal Metal–Organic Framework Materials. Nanoscale 2020, 12 (29), 15577–15587. 10.1039/D0NR01673J. 32510095

[ref35] Farrow C. L.; Juhas P.; Liu J. W.; Bryndin D.; Božin E. S.; Bloch J.; Proffen T.; Billinge S. J. L. PDFfit2 and PDFgui: Computer Programs for Studying Nanostructure in Crystals. J. Phys.: Condens. Matter 2007, 19 (33), 335219 10.1088/0953-8984/19/33/335219. 21694142

[ref36] Alhasni B. Insight into the Structure of Magnesium and Sodium Mixed Phosphate Glasses: A Molecular Dynamics Study. J. Non-Cryst. Solids 2022, 578, 121338 10.1016/j.jnoncrysol.2021.121338.

[ref37] Sapnik A. F.; Bechis I.; Bumstead A. M.; Johnson T.; Chater P. A.; Keen D. A.; Jelfs K. E.; Bennett T. D. Multivariate Analysis of Disorder in Metal–Organic Frameworks. Nat. Commun. 2022, 13 (1), 2173 10.1038/s41467-022-29849-6. 35449202 PMC9023516

[ref38] Zhang Z.; Chen Y.; Wang P.; Wang Z.; Zuo C.; Chen W.; Ao T. Facile Fabrication of N-Doped Hierarchical Porous Carbons Derived from Soft-Templated ZIF-8 for Enhanced Adsorptive Removal of Tetracycline Hydrochloride from Water. J. Hazard. Mater. 2022, 423, 127103 10.1016/j.jhazmat.2021.127103. 34534809

[ref39] Solís R. R.; Gómez-Avilés A.; Belver C.; Rodriguez J. J.; Bedia J. Microwave-Assisted Synthesis of NH2-MIL-125(Ti) for the Solar Photocatalytic Degradation of Aqueous Emerging Pollutants in Batch and Continuous Tests. J. Environ. Chem. Eng. 2021, 9 (5), 106230 10.1016/j.jece.2021.106230.

[ref40] Wang Z.; Kong D.; Wang M.; Wang G.; Li N.; Li D. Sealing Effect of Surface Porosity of Ti–P Composite Films on Tinplates. RSC Adv. 2019, 9 (23), 12990–12997. 10.1039/C8RA10523E. 35520766 PMC9063787

[ref41] Kim S.-N.; Kim J.; Kim H.-Y.; Cho H.-Y.; Ahn W.-S. Adsorption/Catalytic Properties of MIL-125 and NH2-MIL-125. Catal. Today 2013, 204, 85–93. 10.1016/j.cattod.2012.08.014.

[ref42] Nuhnen A.; Janiak C. A Practical Guide to Calculate the Isosteric Heat/Enthalpy of Adsorption via Adsorption Isotherms in Metal–Organic Frameworks, MOFs. Dalton Trans. 2020, 49 (30), 10295–10307. 10.1039/D0DT01784A. 32661527

[ref43] Serafin J.; Dziejarski B. Application of Isotherms Models and Error Functions in Activated Carbon CO2 Sorption Processes. Microporous Mesoporous Mater. 2023, 354, 112513 10.1016/j.micromeso.2023.112513.

[ref44] Chen B.; Wang L.; Gao S. Recent Advances in Aerobic Oxidation of Alcohols and Amines to Imines. ACS Catal. 2015, 5 (10), 5851–5876. 10.1021/acscatal.5b01479.

[ref45] Jiménez-Almarza A.; López-Magano A.; Mas-Ballesté R.; Alemán J. Tuning the Activity–Stability Balance of Photocatalytic Organic Materials for Oxidative Coupling Reactions. ACS Appl. Mater. Interfaces 2022, 14 (14), 16258–16268. 10.1021/acsami.2c01646. 35348315 PMC9011354

[ref46] Yu J. C.; Zhang L.; Zheng Z.; Zhao J. Synthesis and Characterization of Phosphated Mesoporous Titanium Dioxide with High Photocatalytic Activity. Chem. Mater. 2003, 15 (11), 2280–2286. 10.1021/cm0340781.

[ref47] Sun D.; Ye L.; Li Z. Visible-Light-Assisted Aerobic Photocatalytic Oxidation of Amines to Imines over NH2-MIL-125(Ti). Appl. Catal. B 2015, 164, 428–432. 10.1016/j.apcatb.2014.09.054.

[ref48] Wang H.; Yu J.; Wei S.; Lin M.; Song Y.; Wu L. Surface Coordination Enhanced Visible-Light Photocatalytic Coupling of Benzylamine to N-Benzylidene Benzylamine over the Pd/NH2-MIL-125(Ti) Nanosheets. Chem. Eng. J. 2022, 441, 136020 10.1016/j.cej.2022.136020.

[ref49] Palavit G. Phosphate glasses and water. Phosphorus Res. Bull. 1996, 6, 85–90. 10.3363/prb1992.6.0_85.

