# Supplementary material for: Structural and Interfacial Characterization of a Photocatalytic Titanium MOF-Phosphate Glass Composite
Source: ACS Appl Mater Interfaces. 2025 Mar 4;17(10):15793–803. doi: 10.1021/acsami.4c18444 (PMC11912187; doi:10.1021/acsami.4c18444)
Supplement: Supplementary file 1 — am4c18444_si_001.pdf [file am4c18444_si_001.pdf]

Supporting Information

Structural and Interfacial Characterisation of a Photocatalytic Titanium MOF-Phosphate Glass Composite

Celia Castillo-Blas,*[a] Montaña J. García,[b] Ashleigh M. Chester,[a] Matjaž Mazaj,[c] Shaoliang Guan, [a],[d] Georgina P. Robertson,[a],[e] Ayano Kono,[a] James M.A. Steele,[d],[f] Luis León-Alcaide,[g] Bruno Poletto-Rodrigues,[h] Philip. A. Chater,[e] Silvia Cabrera,[i],[j] Andraž Krajnc,[c] Lothar Wondraczek,[h] David A. Keen,[k] Jose J. Alemán,[b] and Thomas D. Bennett*[a]

[a] Department of Materials Science and Metallurgy, University of Cambridge, 27 Charles Babbage Road, CB30FS, Cambridge, United Kingdom

[b] Organic Chemistry Department, Science Faculty, Universidad Autónoma de Madrid, C/ Francisco Tomás y Valiente, 7, 28049, Madrid, Spain

[c] National Institute of Chemistry, Hajdrihova 19, SI-1000 Ljubljana, Slovenia

[d] Cavendish Laboratory, University of Cambridge, J. J. Thomson Avenue, Cambridge, CB3 0HE United Kingdom

[e] Diamond Lightsource Ldt., Diamond House, Harwell Campus, Didcot, OX110QX, Oxfordshire, United Kingdom

[f] Yusuf Hamied Department of Chemistry, University of Cambridge, Lensfield Road, Cambridge, CB2 1EW, United Kingdom

[g] Instituto de Ciencia Molecular (ICMol), Universidad de Valencia, c/Catedrático José Beltrán 2, Paterna 46980, Spain

[h] Otto-Schott Institute of Materials Research, University of Jena, Fraunhoferstrasse 6, 07743 Jena, Germany

[i] Inorganic Chemistry Department, Science Faculty, Universidad Autónoma de Madrid, C/ Francisco Tomás y Valiente, 7, 28049, Madrid, Spain

[j] Institute for Advanced Research in Chemical Sciences (IAdChem), Universidad Autónoma de Madrid, C/ Francisco Tomás y Valiente, 7, 28049, Madrid, Spain

[k] ISIS, Rutherford Appleton Laboratory, Harwell Campus, Didcot, OX110QX, Oxfordshire, United Kingdom

* Contact corresponding authors: cc2078@cam.ac.uk, thomas.bennett@canterbury.ac.nz

Table of contents

1. Characterisation of MIL-125-NH_2 3
2. Characterisation of the inorganic glass 5
3. Composites synthesis. 7
4. Powder X-ray diffraction 8
5. Elemental analysis and molar calculations 16
6. Fourier Transformed Infrared Spectroscopy 17
7. Scanning electron microscopy study 18
8. Thermal characterisation 27
9. Pair distribution function study 32
10. X-ray photoelectron spectroscopy (XPS) 43
11. Gas sorption 48
    11.1 Nitrogen isotherms 48
    11.2 Carbon dioxide isotherms 49
    11.3 Heat of adsorption 52
12. Photocatalytic activity 55
    12.1 Photocatalytic oxidative coupling of benzylamines. Optimisation of the reaction conditions 56
    12.2 General procedure for the oxidative coupling of amines 59
13. ^{1}H Nuclear magnetic resonance spectra 62
14. Stability study 66
15. References 69

1. Characterisation of MIL-125-NH_2

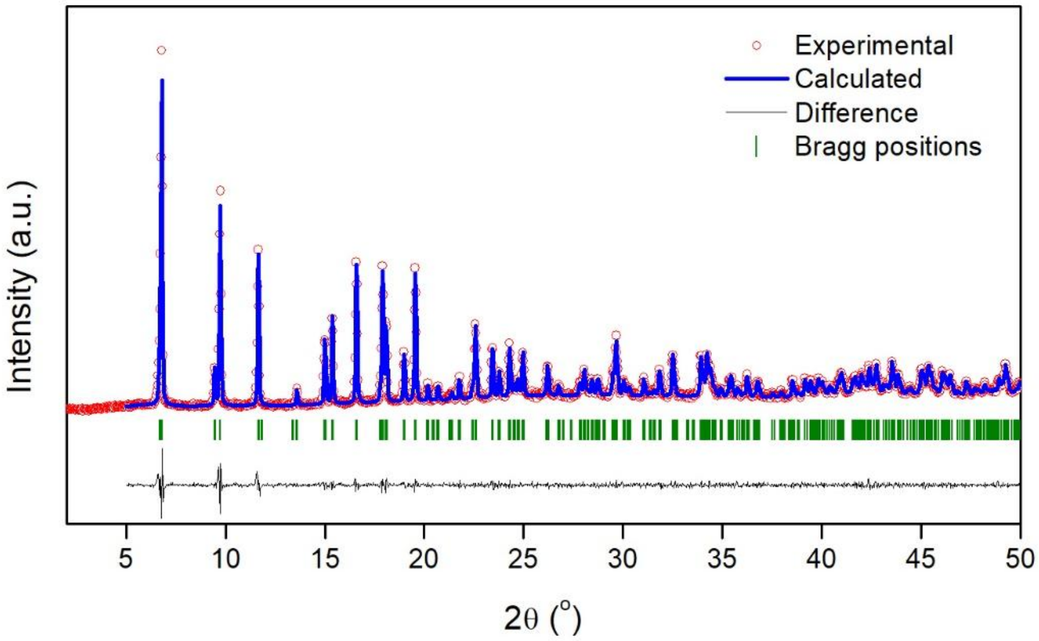

Figure S1. Experimental (red dots), calculated (blue line), difference plot plot [($I_{obs}-I_{calc}$)] (black line) and Bragg positions (green ticks) for the Pawley refinement of experimental diffraction data with the obtained cell: R_{wp} = 8.97%, R_p = 6.39% (λ = 1.5406 Å). Initial parameters were obtained from the CIF file previously reported for crystalline MIL-125.[1]

Table S1. Pawley refinement details.

2θ range (º)	R_{wp} %	Space group	Zero	Lattice parameter / Å	Profile parameters	Lattice parameter reported from [1]
5-50	8.97%	*I4/mmm*	-0.0015(8)	a = 18.7072 (9)<br>c = 18.1980 (11)	U = 0.059 (17)<br>V = -0.007 (6)<br>W = 0.0060 (5)	a = 18.6453(10) Å<br>c = 18.1444(10) Å

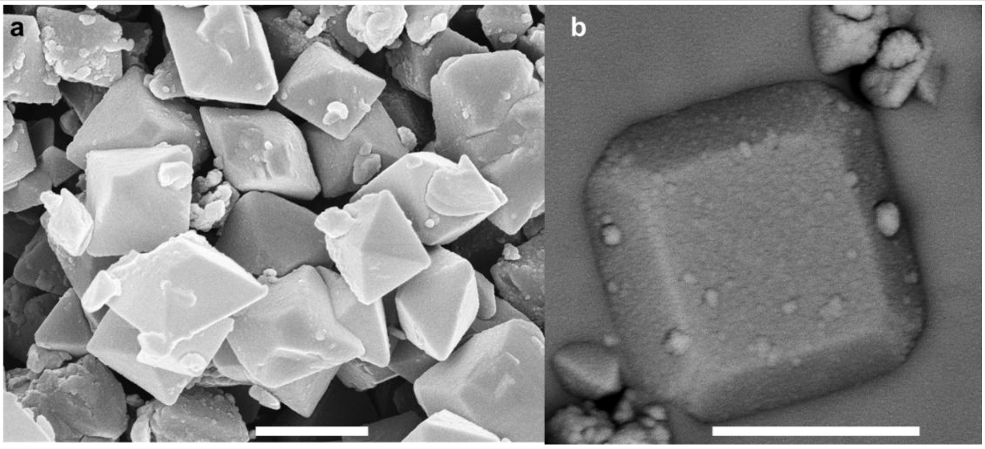

Figure S2. SEM images of activated pristine Ti-MOF (MIL-125-NH_2). White bar measurement has a length of 1 µm.

Table S2. Formula and CHN analysis.

CHN analysis experimental	Formula found
C 33.84%, H 3.42%, N 4.83%	$[Ti_8O_{36}H_{44}N_6C_{48}](H_2O)_{1.2}(DMF)_{0.3}$

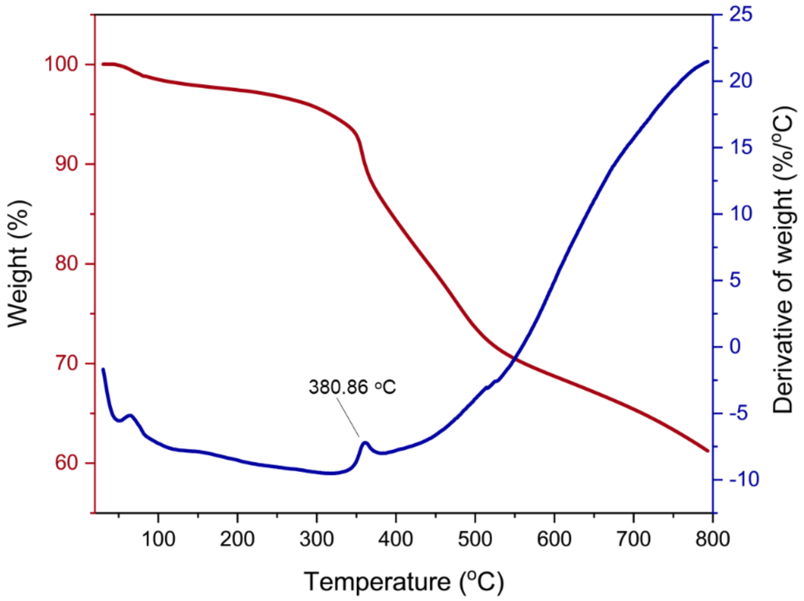

Figure S3. Thermogravimetric analysis of the activated Ti-MOF heated at 10 ºC/min up to 800 ºC. Weight (%) curve shown in scarlet and derivative weight (%/ºC) shown in blue. Decomposition temperature (T_d) was calculated using the TA Instrument analysis software package. Weight loss at 100 ºC (~2%) corresponds to adsorbed water loss.

2. Characterisation of the inorganic glass

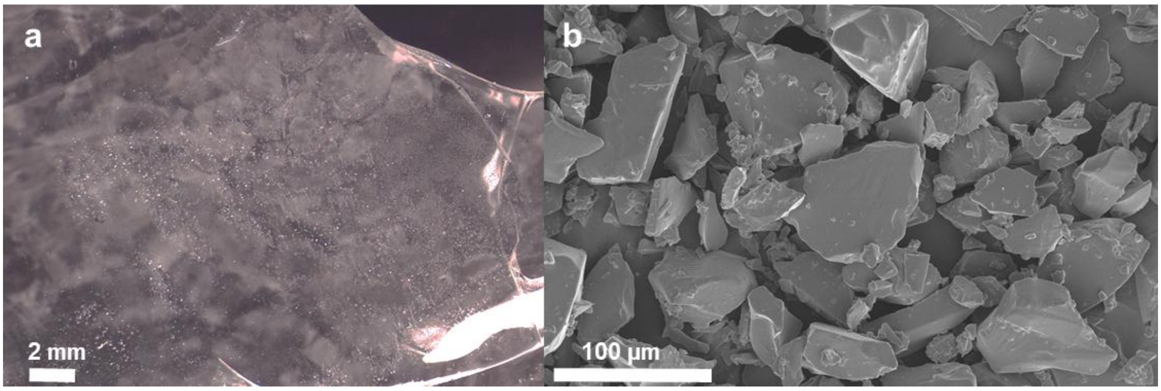

Figure S4. a. Optical image of the 70%P_2O_5-20%Na_2O-10%Na_2SO_4 inorganic glass before. **b.** SEM image after ball milled at 30 Hz for 30 min.

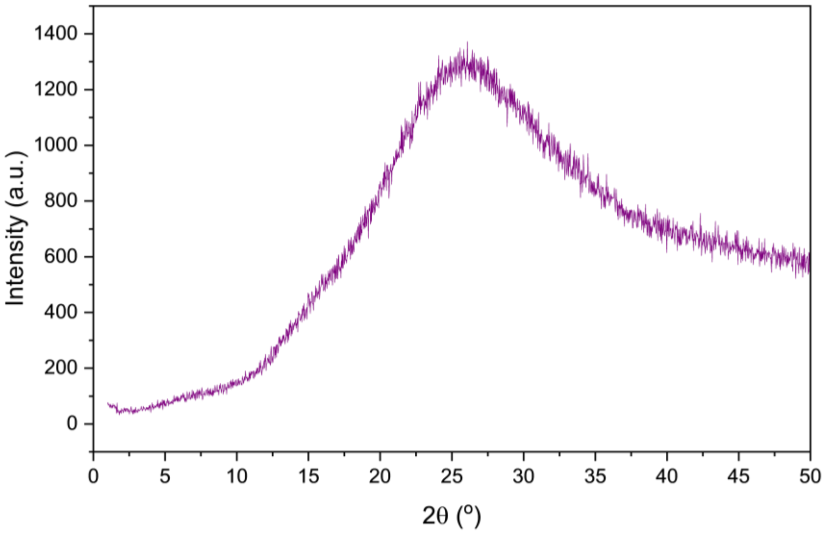

Figure S5. PXRD of 70%P_2O_5-20%Na_2O-10%Na_2SO_4 glass (IG) showing a typical pattern for an amorphous material.

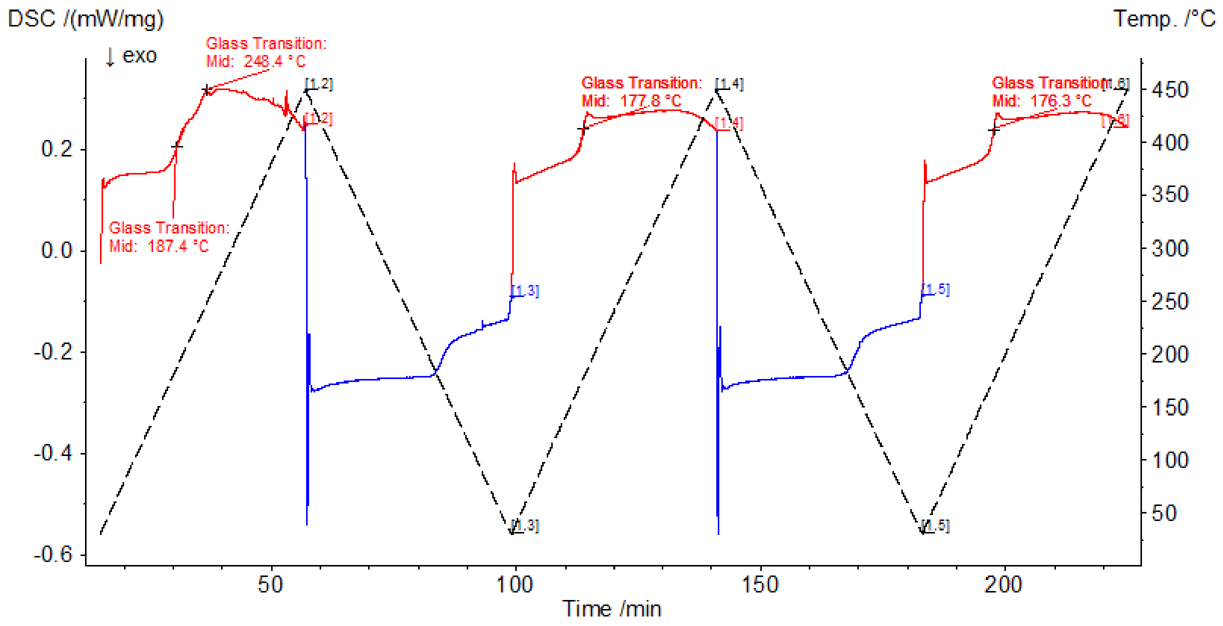

Figure S6. DSC heating/cooling cycles of 70%P_2O_5-20%Na_2O-10%Na_2SO_4 glass performed at a maximum temperature of 450 ºC under an argon atmosphere with a heating/cooling rate of 10 ºC/min.

3. Composites synthesis.

- Optimisation of the pressure for the pellet formation

Applied pressure (0.074 GPa) was considered as the optimum one, as applying higher pressures (0.22 GPa, 0.44 GPa and 0.74 GPa) Ti-MOF lose most intensity of Bragg peaks and after heating, other crystalline phases are formed.

- Optimisation of the temperature for the successfully composite formation

The optimised temperature was 180 ºC as it allowed the inorganic glass flow around the MOF crystallites and avoid the decomposition of the MOF.

4. Powder X-ray diffraction

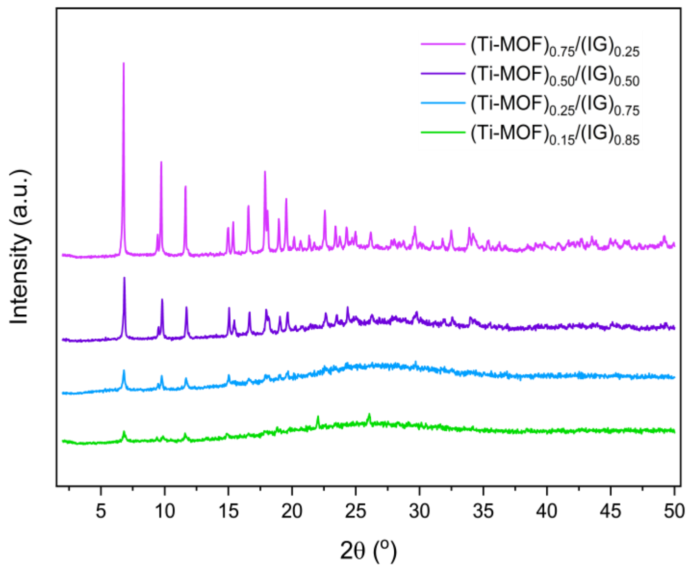

Figure S7. PXRD of the physical mixtures before pelletisation.

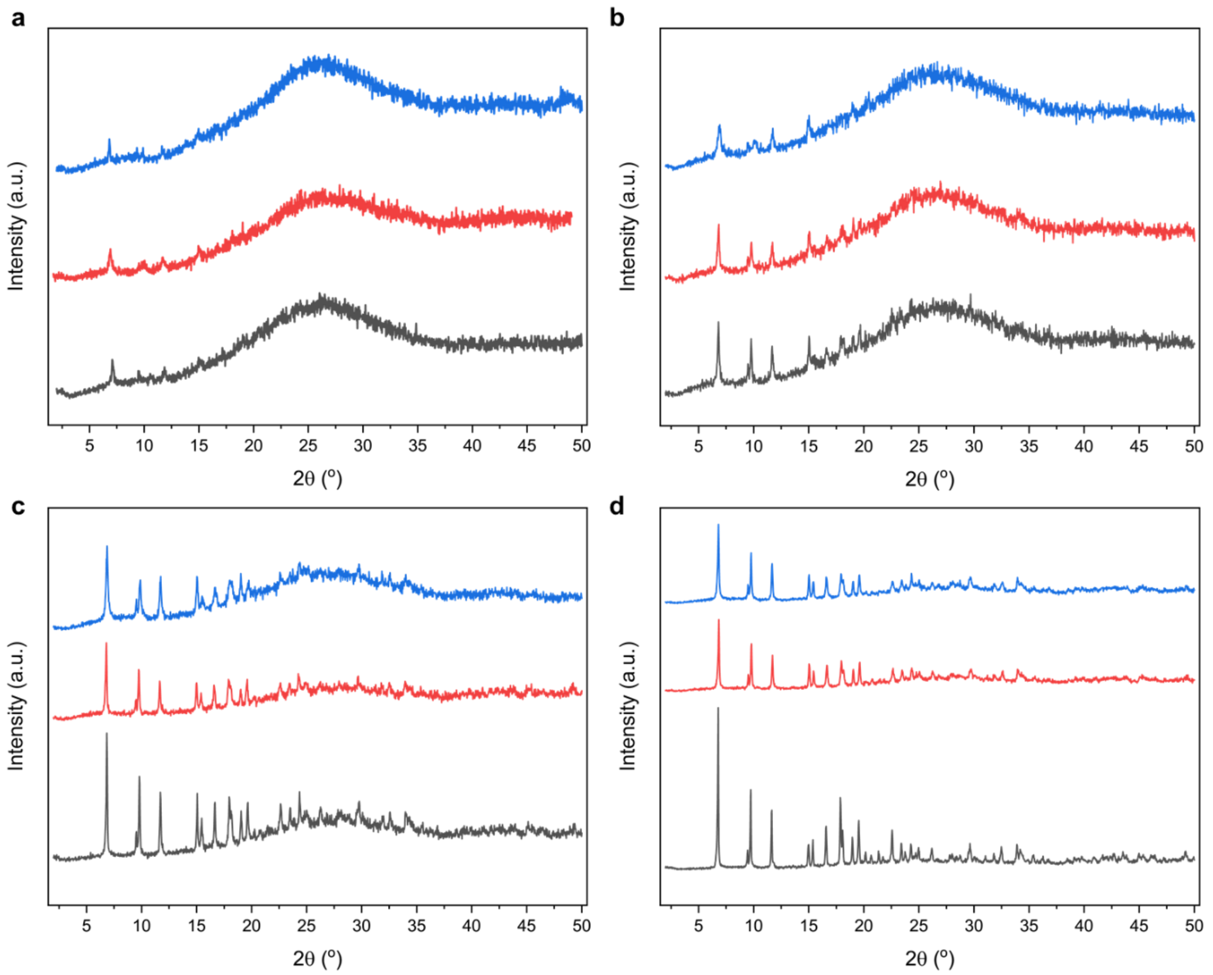

Figure S8. PXRD patterns from different stages of composite sample preparation showing: the physical mixture (sample after ball milling) (grey), after pelletisation (red) and the composite after thermal treatment (blue). **a**. [(Ti-MOF)$_{0.15}$(IG)$_{0.85}$]; **b**. [(Ti-MOF)$_{0.25}$(IG)$_{0.75}$]; **c**. [(Ti-MOF)$_{0.50}$(IG)$_{0.50}$] and **d**. [(Ti-MOF)$_{0.75}$(IG)$_{0.25}$].

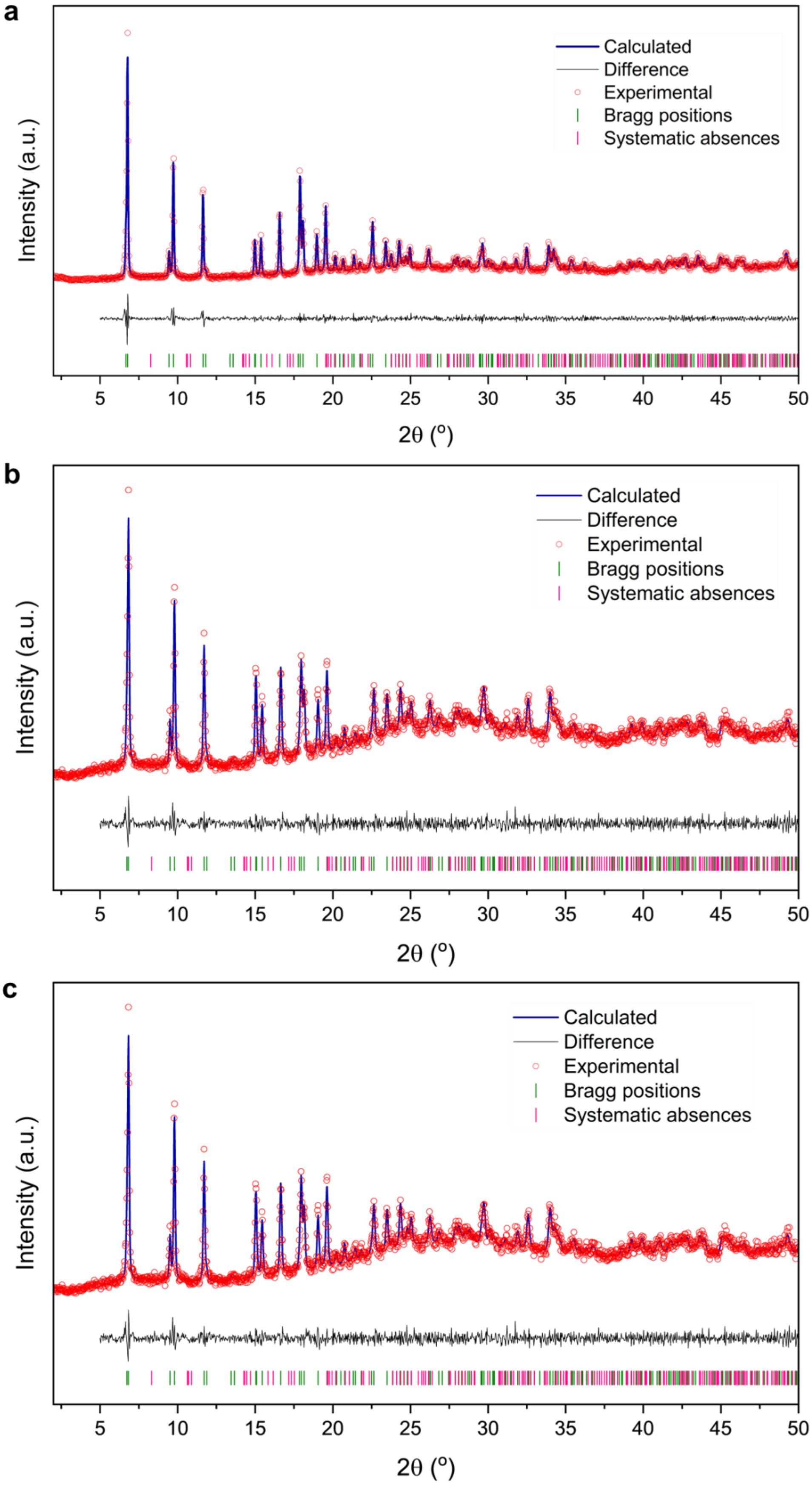

Figure S9. Pawley refinements for the composite containing 75% of Ti-MOF. **a**. physical mixture., **b**. pelletised sample and **c**. composite [(Ti-MOF)$_{0.75}$(IG)$_{0.25}$].

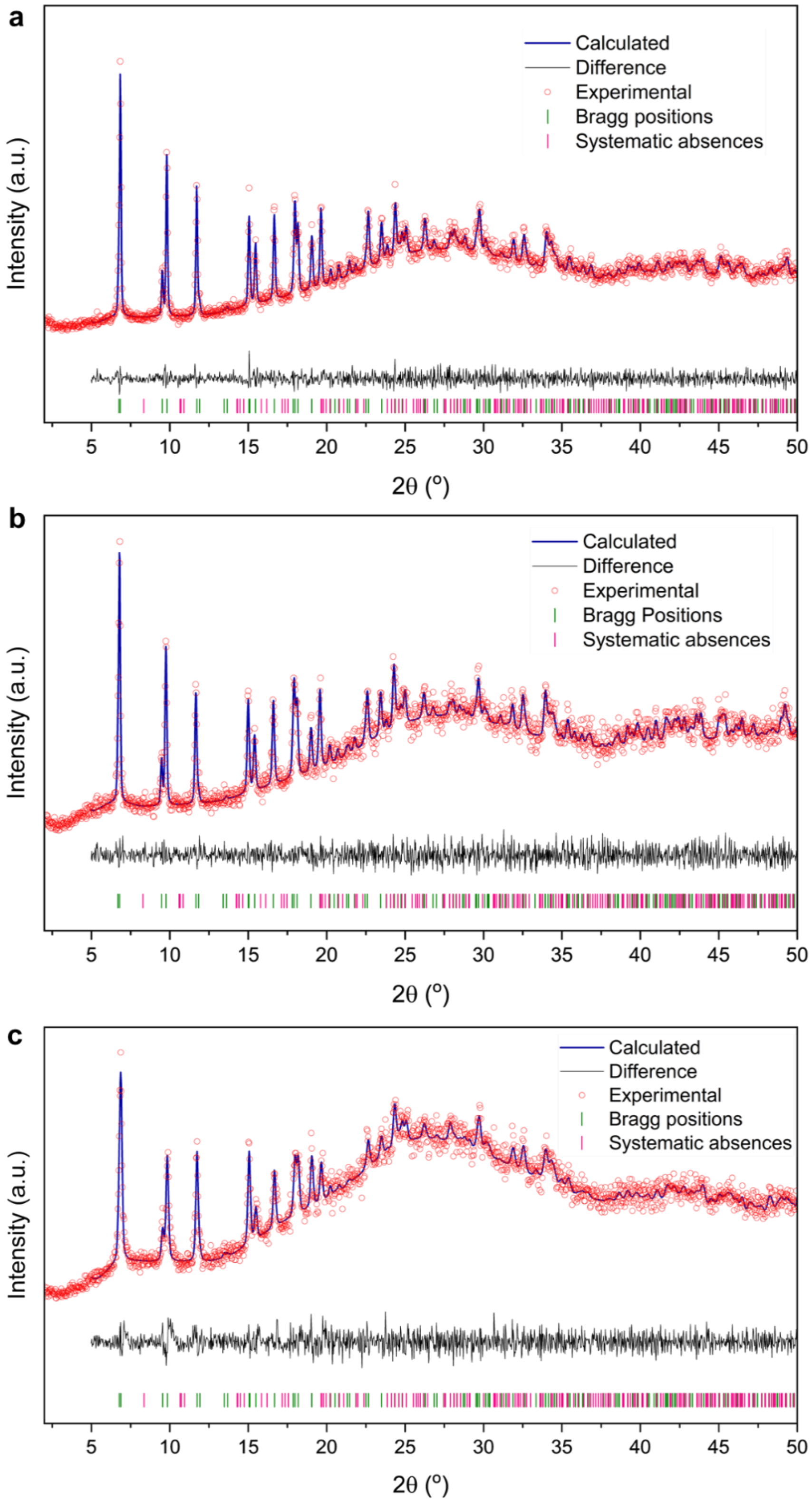

Figure S10. Pawley refinements for the composite containing 50% of Ti-MOF. **a**. physical mixture., **b**. pelletised sample and **c**. composite [$(Ti\text{-}MOF)_{0.50}(IG)_{0.50}$].

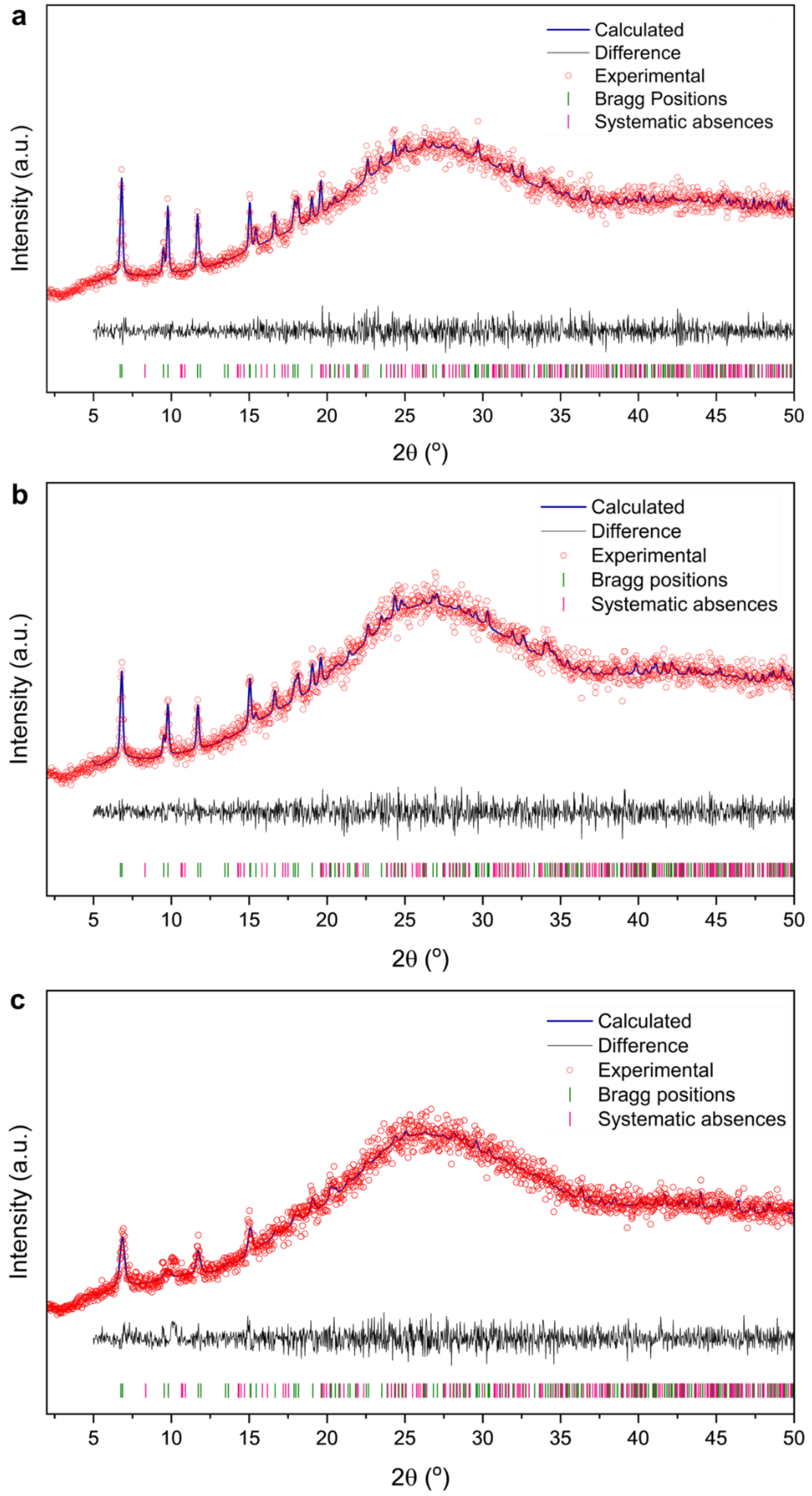

Figure S11. Pawley refinements for the composite containing 25% of Ti-MOF. **a**. physical mixture., **b**. pelletised sample and **c**. composite $[(Ti\text{-}MOF)_{0.25}(IG)_{0.75}]$.

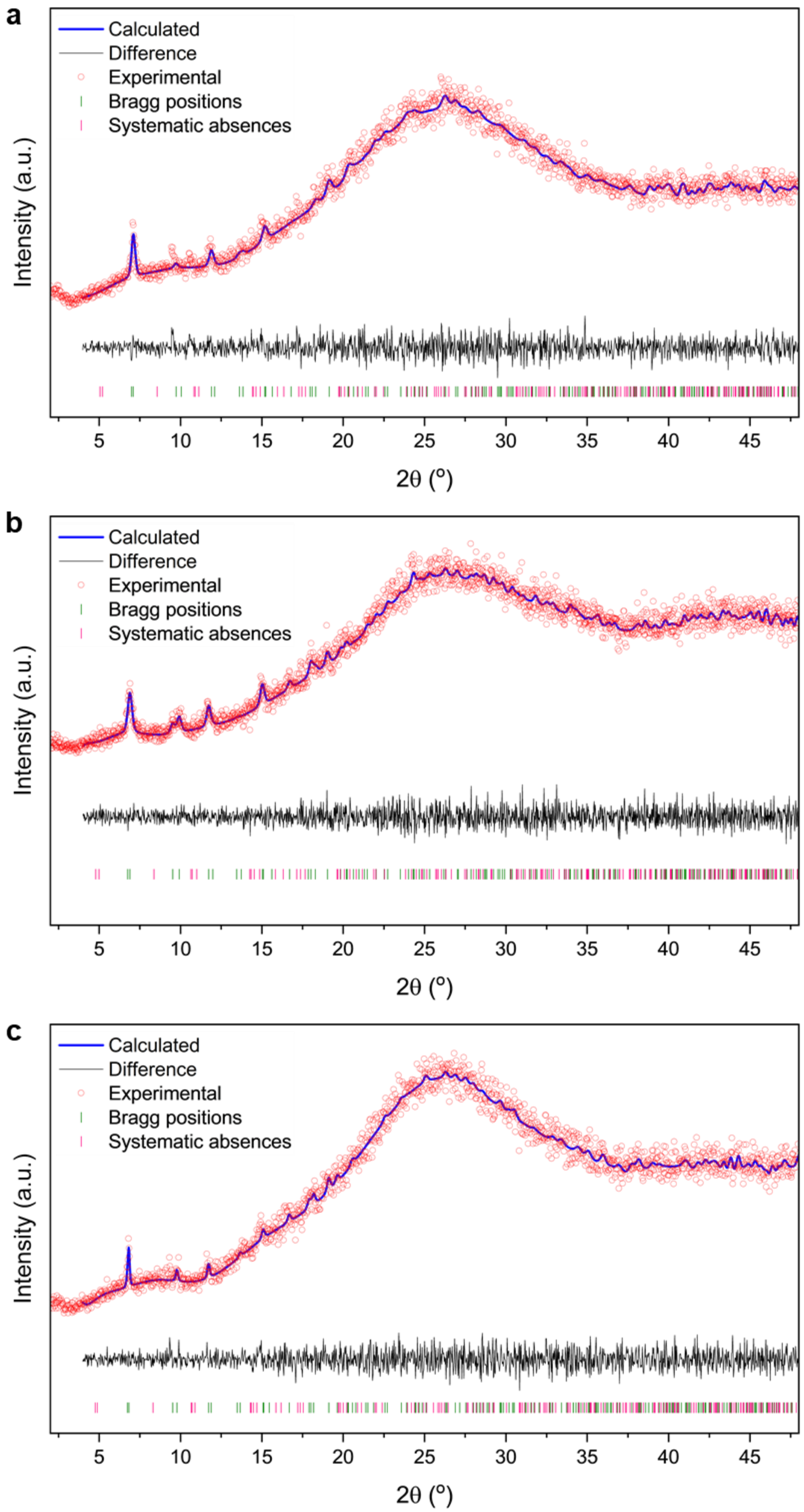

Figure S12. Pawley refinements for the composite containing 15% of Ti-MOF. **a**. physical mixture., **b**. pelletised sample and **c**. composite [(Ti-MOF)$_{0.15}$(IG)$_{0.85}$].

Table S3. Summary of results from Pawley refinements.

Sample	R_{wp}, R_p / %	Space group	Zero	Lattice parameter / Å	Profile parameters	Lattice parameter reported from [1]
(Ti-MOF)$_{0.15}$/(IG)$_{0.85}$	6.39, 4.92	*I4/mmm*	0.038 (9)	a = 18.907 (7)<br>c = 18.284 (7)	U = 0.014 (83)<br>V = 0.94 (5)<br>W = 0.017 (30)	
(Ti-MOF)$_{0.25}$/(IG)$_{0.75}$	6.46, 4.97	*I4/mmm*	0.0315 (31)	a = 18.683 (4)<br>c = 18.134 (5)	U = -0.14 (11)<br>V = -0.053 (6)<br>W = 0.02 (5)	
(Ti-MOF)$_{0.50}$/(IG)$_{0.\ 50}$	6.98, 5.46	*I4/mmm*	0.0533 (15)	a = 18.6658 (19)<br>c = 18.1168 (26)	U = 0.37 (7)<br>V = -0.017 (23)<br>W = 0.0095 (15)	
(Ti-MOF)$_{0.75}$/(IG)$_{0.25}$	8.43, 6.54	*I4/mmm*	-0.0125(7)	a = 18.6864 (9)<br>c = 18.1454 (12)	U = 0.113 (25)<br>V = -0.029 (8)<br>W = 0.0049 (5)	
Pellet (Ti-MOF)$_{0.15}$/(IG)$_{0.85}$	6.15, 4.75	*I4/mmm*	0.0017 (29)	a = 18.709 (5)<br>c = 17.950 (4)	U = 0.35 (14)<br>V = -0.21 (12)<br>W = 0.112 (20)	
Pellet (Ti-MOF)$_{0.25}$/(IG)$_{0.75}$	6.57,5.03	*I4/mmm*	0.0366 (25)	a = 18.654 (16)<br>c = 18.146 (24)	U = -0.36 (18)<br>W = 0.019 (7)	
Pellet (Ti-MOF)$_{0.50}$/(IG)$_{0.50}$	7.51, 5.84	*I4/mmm*	0.010 (2)	a = 18.6772 (26)<br>c = 18.1382 (36)	U = 0.062 (7)<br>V = -0.074 (23)<br>W = 0.0103 (16)	a = 18.6453(10) Å<br>c = 18.1444(10) Å
Pellet (Ti-MOF)$_{0.75}$/(IG)$_{0.25}$	7.81, 5.55	*I4/mmm*	0.0119(14)	a = 18.6612 (18)<br>c = 18.1211 (23)	U = 0.062 (7)<br>V = -0.074 (23)<br>W = 0.0103 (16)	
[(Ti-MOF)$_{0.15}$(IG)$_{0.85}$]	5.63, 4.37	*I4/mmm*	-0.011 (8)	a = 18.559 (5)<br>c = 18.069 (4)	U = 0.56 (29)<br>V = 0.31 (27)<br>W = -0.002 (16)	
[(Ti-MOF)$_{0.25}$(IG)$_{0.75}$]	6.72,5.11	*I4/mmm*	0.065(5)	a = 18.654 (9)<br>c = 18.1444 (18)	U = 0.257 (87)<br>V = -0.43 (8)<br>W = 0.15 (2)	
[(Ti-MOF)$_{0.50}$(IG)$_{0.\ 05}$]	6.98, 5.28	*I4/mmm*	-0.0114(8)	a = 18.6905 (9)<br>c = 18.1460 (13)	U = 0.178 (27)<br>V = 0.016 (8)<br>W = 0.0056 (6)	
[(Ti-MOF)$_{0.75}$(IG)$_{0.25}$]	7.89, 6.08	*I4/mmm*	0.0406 (14)	a = 18.6616 (17)<br>c = 18.1221 (22)	U = 0.265 (17)<br>V = -0.029 (22)<br>W = 0.0100 (15)	

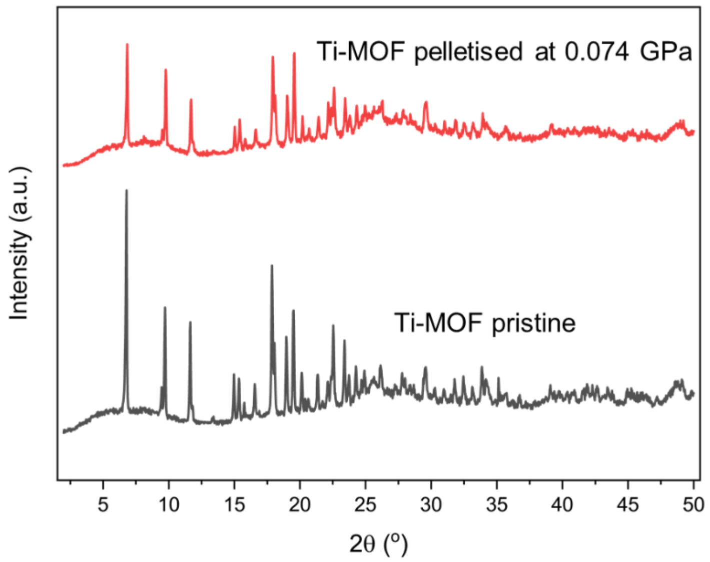

Figure S13. Comparison of the pristine Ti-MOF before and after pelletisation at 0.074 GPa.

5. Elemental analysis and molar calculations

Table S4. Molar ratios, formula and CHN analysis details for all the composites.

**Sample**	**Molar ratio MOF:IG**	**Formula**	**CHN analysis Experimental, Calculated (%)**	**Formula in molar**
[(Ti-MOF)$_{0.75}$(IG)$_{0.25}$]	3.94:1	$Ti_{31.5}C_{189}H_{134}N_{23.6}Na_6O_{183}P_{14}S$	C (27.31, 29.20)<br>H (2.54, 1.74)<br>N (3.76, 4.25)	[(Ti-MOF)$_{3.94}$(IG)]·DMF$_{0.87}$
[(Ti-MOF)$_{0.50}$(IG)$_{0.50}$]	1.31:1	$Ti_{10.5}C_{63}H_{44.3}N_{7.9}Na_6O_{88}P_{14}S$	C (21.24, 22.09)<br>H (2.46, 1.30)<br>N (2.89, 3.23)	[(Ti-MOF)$_{1.31}$(IG)]·DMF$_{0.35}$
[(Ti-MOF)$_{0.25}$(IG)$_{0.75}$]	1:2.28	$Ti_{3.5}C_{21}H_{14.9}N_{2.6}Na_6O_{57}P_{14}S$	C (11.97, 12.70)<br>H (1.98, 0.76)<br>N (1.54, 1.83)	[(Ti-MOF)(IG)$_{2.28}$]·DMF$_{0.18}$
[(Ti-MOF)$_{0.15}$(IG)$_{0.85}$]	1:4.32	$Ti_{1.85}C_{11}H_8N_{1.4}Na_6O_{49}P_{14}S$	C (8.54, 8.08)<br>H (2.12, 0.49)<br>N (1.17, 1.20)	[(Ti-MOF)(IG) $_{4.32}$]·DMF$_{0.03}$
IG	NA	$P_{14}Na_6O_{41}S$	NA	NA

6. Fourier Transformed Infrared Spectroscopy

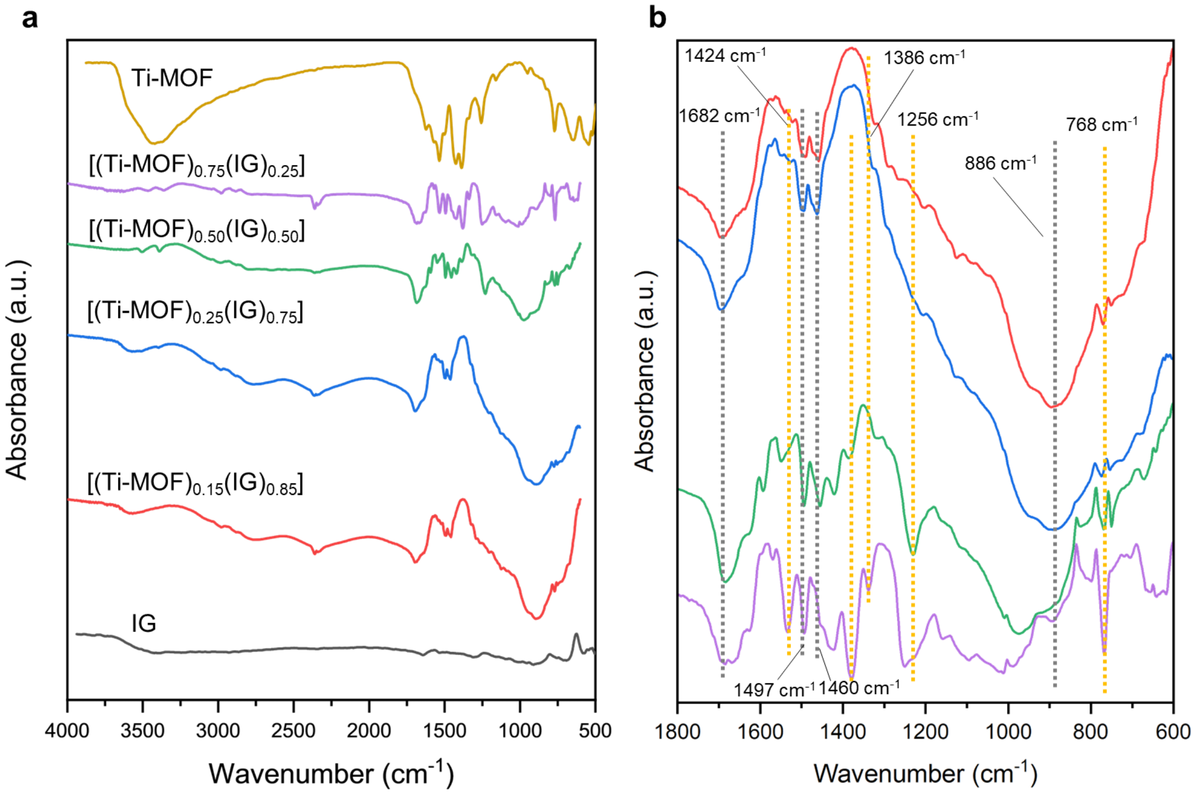

Figure S14. a. FTIR of all the composites and pristine materials. **b**. FTIR of the composites [(Ti-MOF)$_{0.15}$(IG)$_{0.85}$], [(Ti-MOF)$_{0.25}$(IG)$_{0.75}$], [(Ti-MOF)$_{0.50}$(IG)$_{0.50}$], and [(Ti-MOF)$_{0.75}$(IG)$_{0.25}$] are depicted in red, blue, green and violet, respectively. Straight dotted lines are showing the principal bands of the Ti-MOF (yellow) and the IG (grey). Main peaks of the Ti-MOF FTIR spectrum: 768, 1256, 1386, 1424, 1534 cm^{-1}. Main peaks of the inorganic glass FTIR spectrum: 886, 1460, 1497, 1682 cm-^{1}.

7. Scanning electron microscopy study

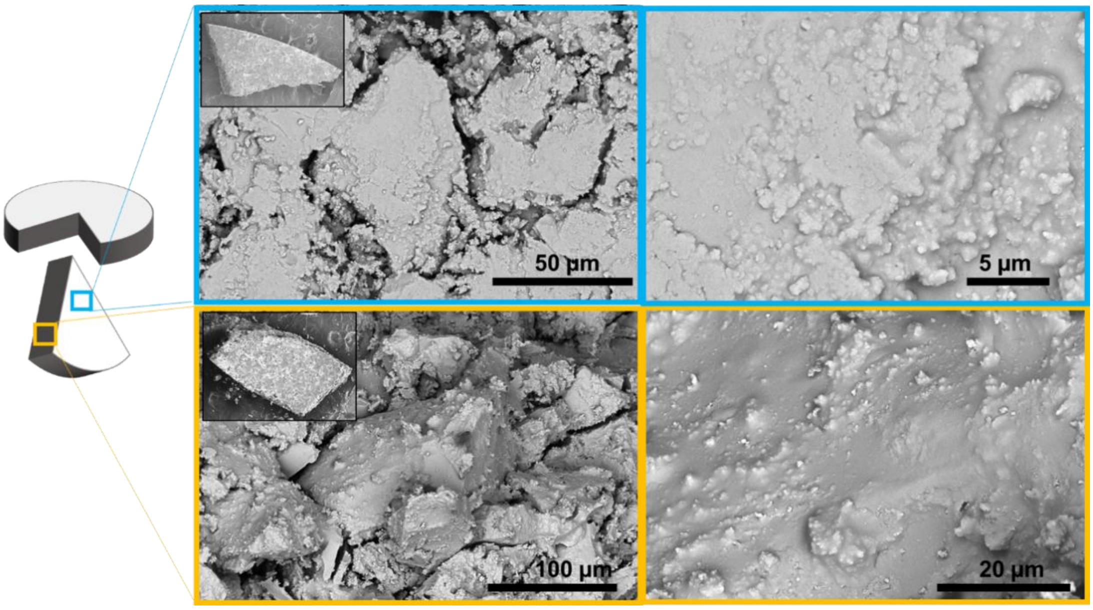

Figure S15. Scheme of a pellet piece of [$(Ti\text{-}MOF)_{0.75}(IG)_{0.25}$] composite (left) where SEM images with cyan edge were taken from the surface of the pellet (up). Yellow-edges images from inside the pellet showing MOF crystallites are embedded by the inorganic glass matrix. Insets show the pellet pieces with 1mm of diameter.

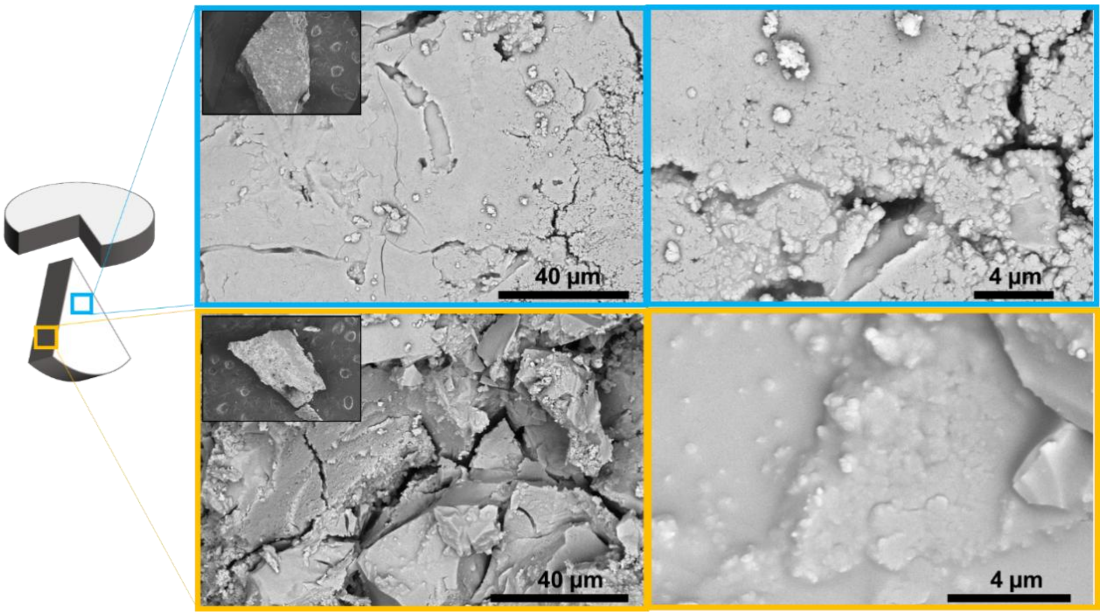

Figure S16. Scheme of a pellet piece of [$(Ti\text{-}MOF)_{0.50}(IG)_{0.50}$] composite (left) where SEM images with cyan edge were taken from the surface of the pellet (up). Yellow-edges images from inside the pellet showing MOF crystallites are embedded by the inorganic glass matrix. Insets show the pellet pieces with 1mm of diameter.

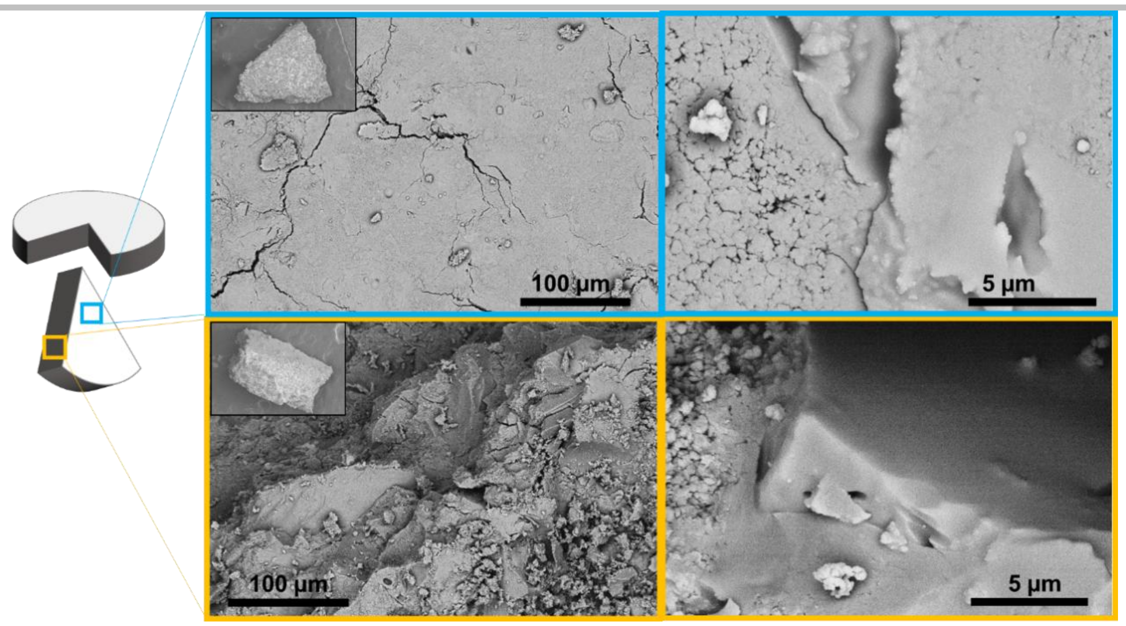

Figure S17. Scheme of a pellet piece of [(Ti-MOF)$_{0.25}$(IG)$_{0.75}$] composite (left) where SEM images with cyan edge were taken from the surface of the pellet (up). Yellow-edges images from inside the pellet showing MOF crystallites are embedded by the inorganic glass matrix. Insets show the pellet pieces with 1mm of diameter.

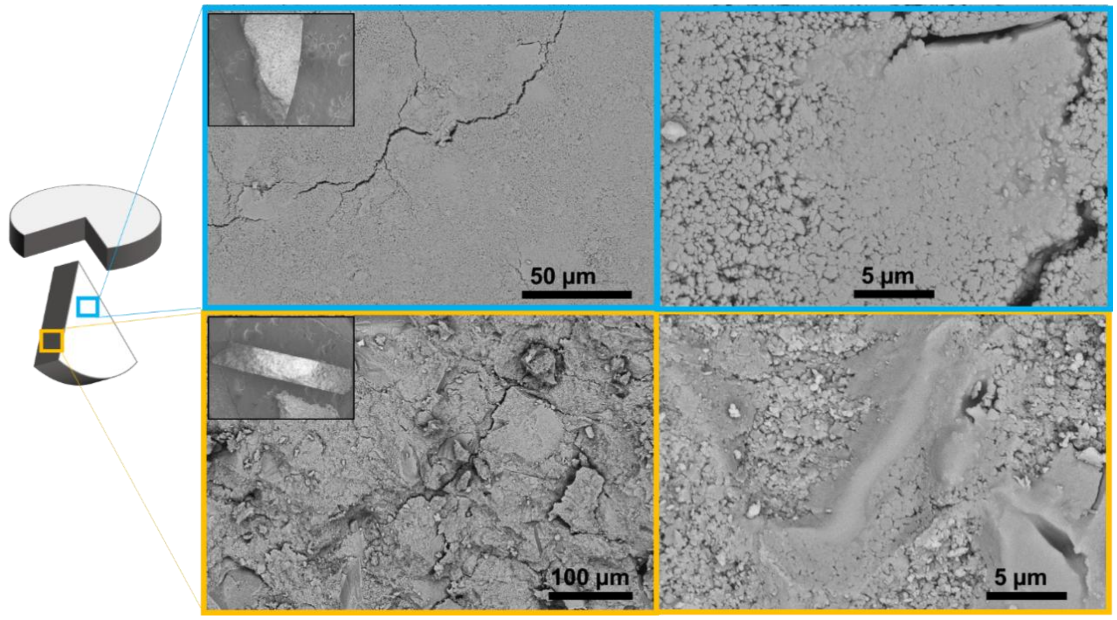

Figure S18. Scheme of a pellet piece of [(Ti-MOF)$_{0.15}$(IG)$_{0.85}$] composite (left) where SEM images with cyan edge were taken from the surface of the pellet (up). Yellow-edges images from inside the pellet showing MOF crystallites are embedded by the inorganic glass matrix. Insets show the pellet pieces with 1mm of diameter.

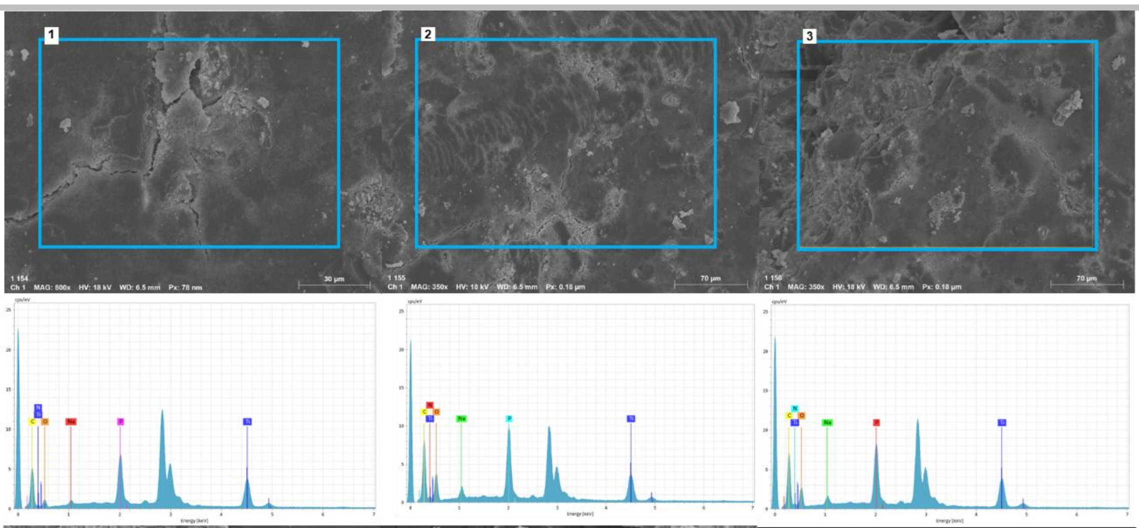

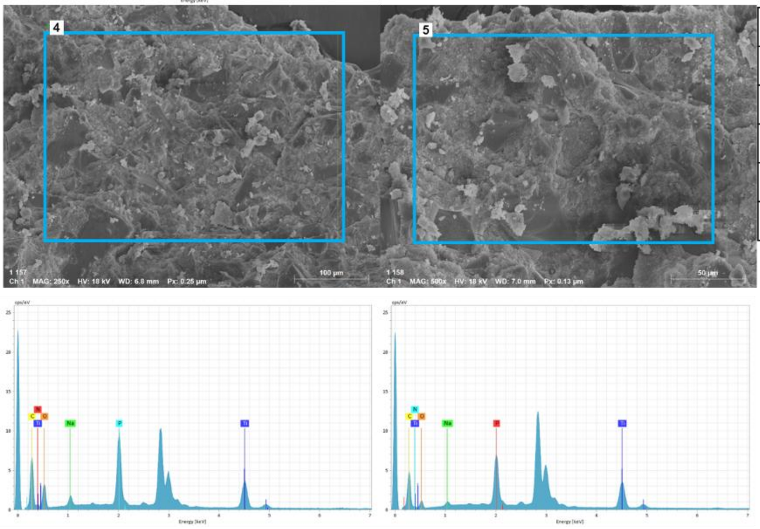

Spectrum	%Ti	%P	%Na
1	22.81	4.24	1.09
2	22.80	3.78	0.85
3	15.96	3.87	0.64
4	13.79	4.80	1.03
5	14.93	5.40	0.80

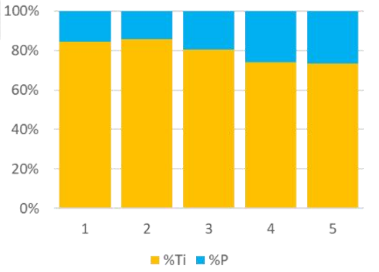

Figure S19. EDX-analyses of [(Ti-MOF)$_{0.75}$(IG)$_{0.25}$] composite. Five different areas of the material depicted in blue were analysed with their corresponding spectrum. % Weight of Na, P and Ti results were summarised in tables and graph (bottom left).

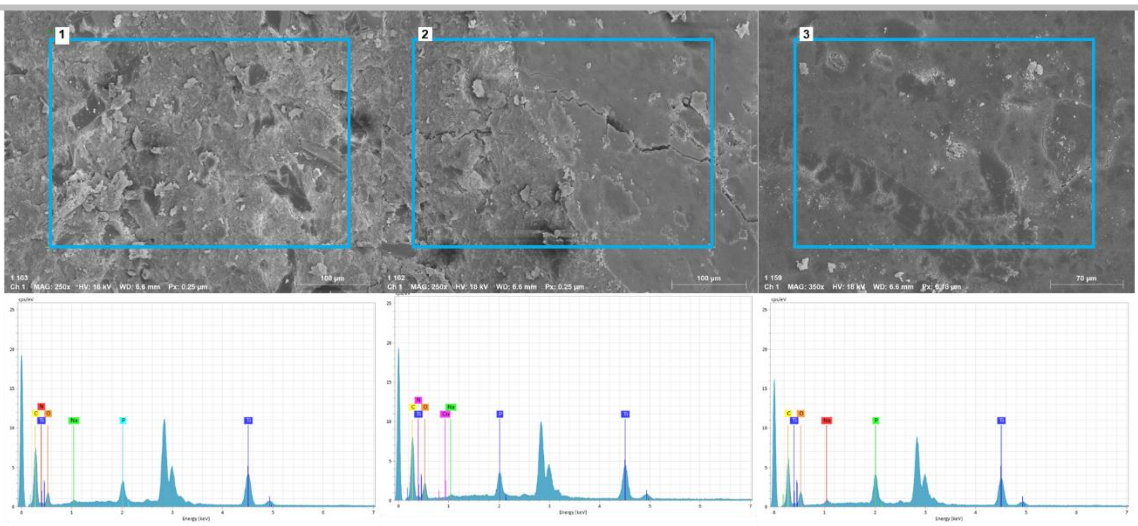

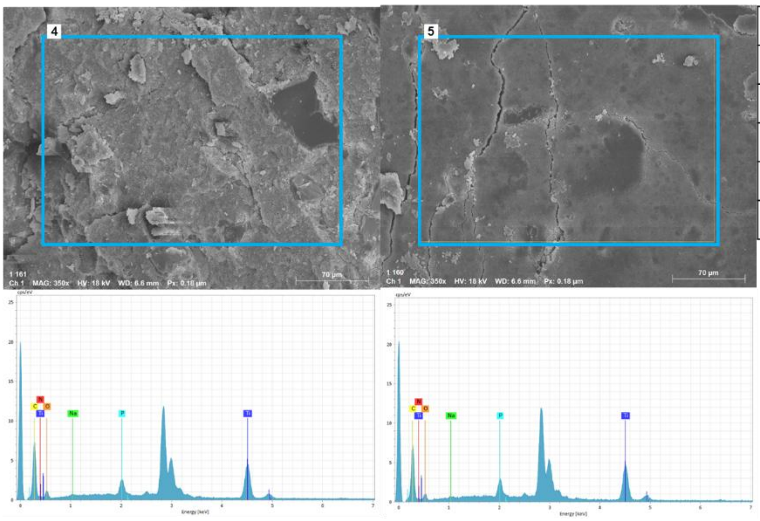

Spectrum	%Ti	%P	%Na
1	20.71	1.92	0.00
2	19.91	1.29	0.00
3	18.27	2.21	0.10
4	24.70	3.84	0.34
5	24.35	1.98	0.28

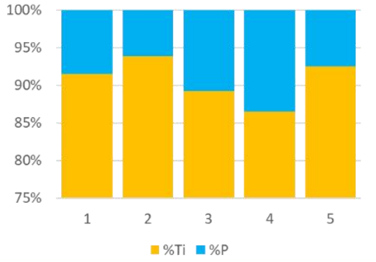

Figure S20. EDX-analyses of [(Ti-MOF)$_{0.50}$(IG)$_{0.50}$] composite. Five different areas of the material depicted in blue were analysed with their corresponding spectrum. % Weight of Na, P and Ti results were summarised in tables and graph (bottom left).

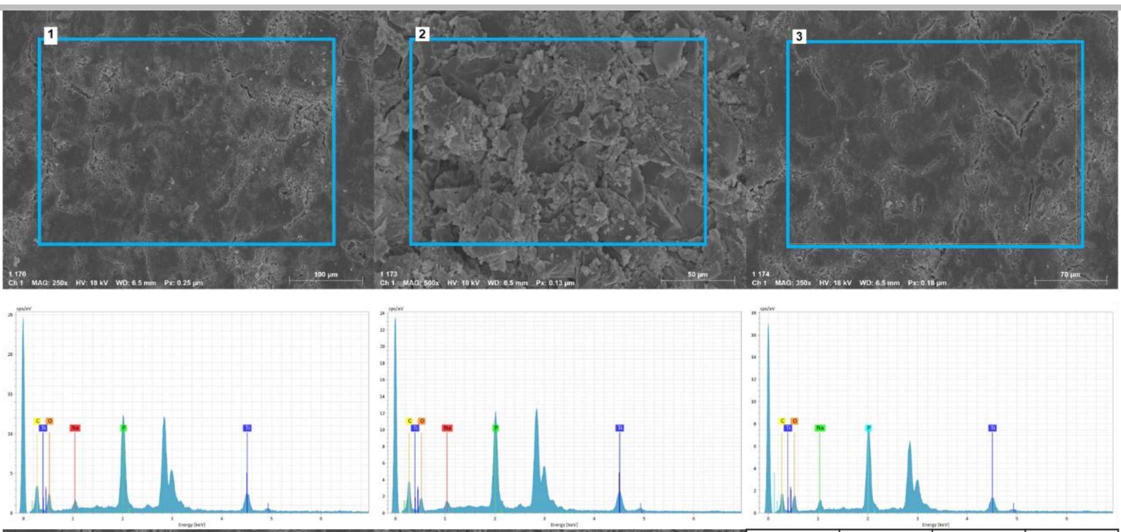

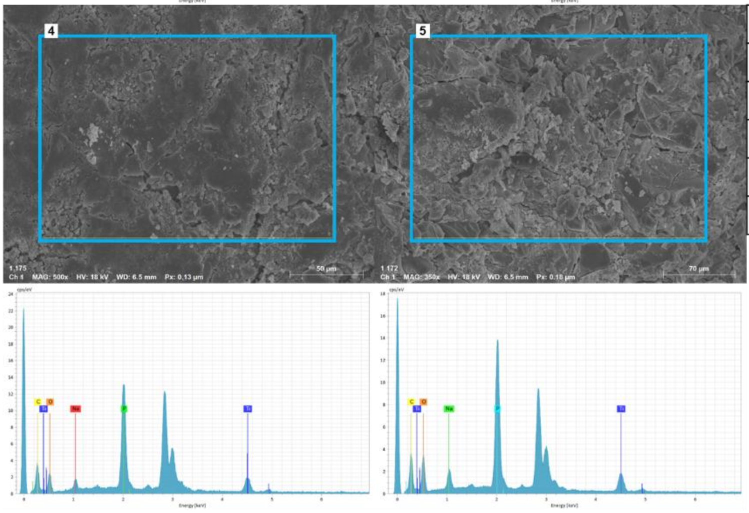

Spectrum	%Ti	%P	%Na
1	15.10	13.56	0.15
2	11.42	13.32	0.23
3	17.90	10.36	0.19
4	13.51	12.76	2.25
5	15.78	12.94	1.83

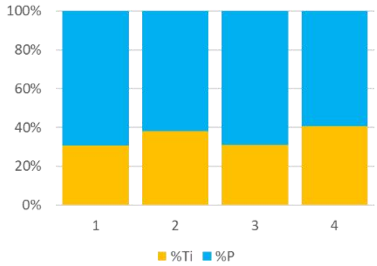

Figure S21. EDX-analyses of $[(Ti\text{-}MOF)_{0.25}(IG)_{0.75}]$ composite. Five different areas of the material depicted in blue were analysed with their corresponding spectrum. % Weight of Na, P and Ti results were summarised in tables and graph (bottom left).

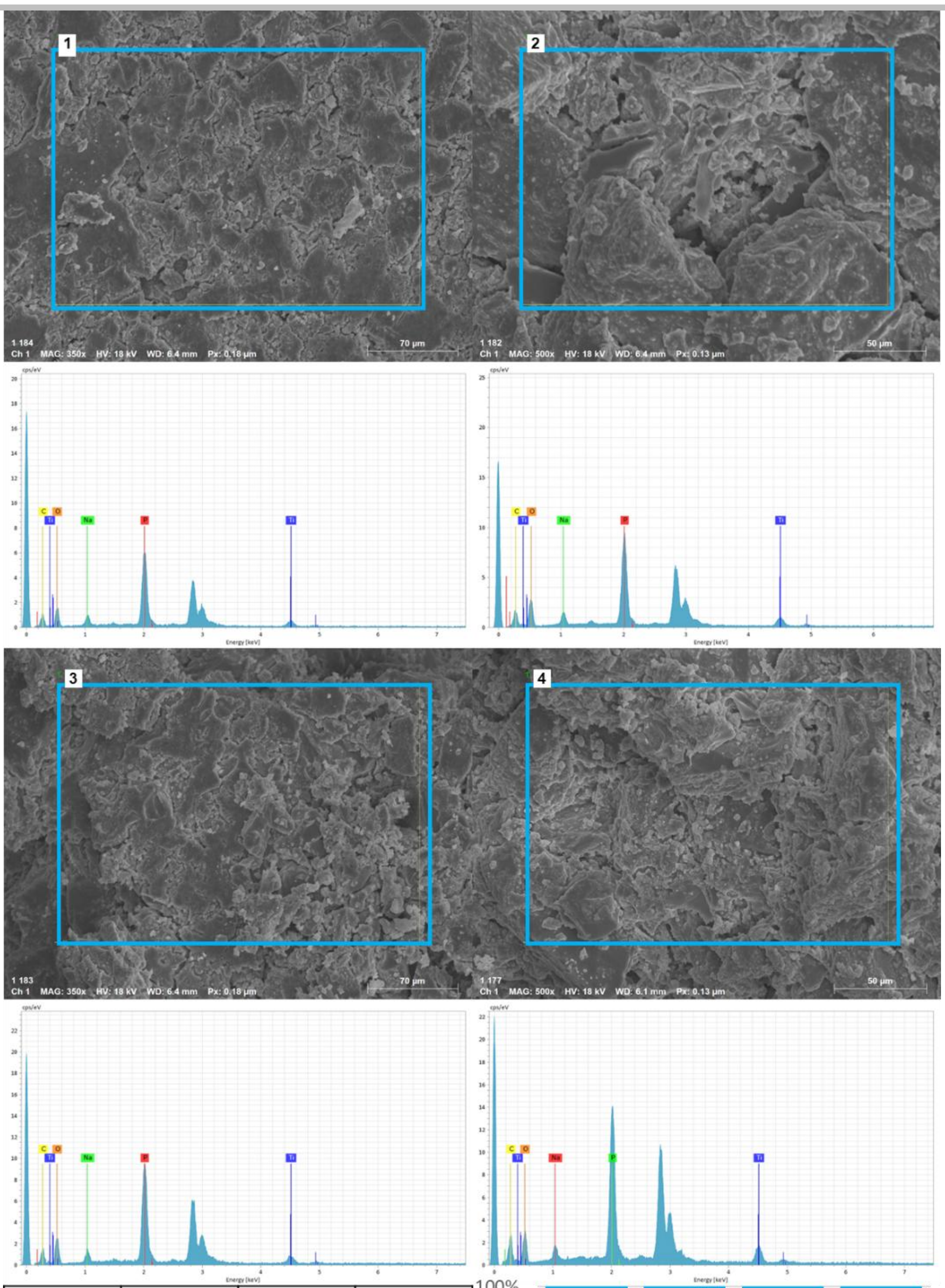

Spectrum	%Ti	%P	%Na
1	7.97	17.96	3.73
2	9.63	15.59	3.86
3	8.41	18.76	3.61
4	12.00	17.50	2.47

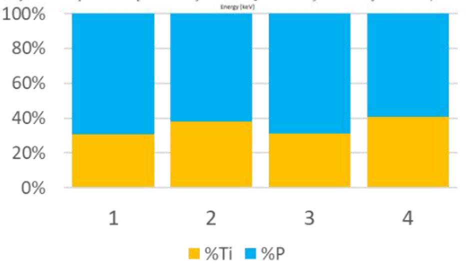

Figure S22. EDX-analyses of $[(Ti\text{-}MOF)_{0.15}(IG)_{0.85}]$ composite. Four different areas of the material depicted in blue were analysed with their corresponding spectrum. % Weight of Na, P and Ti results were summarised in tables and graph (bottom).

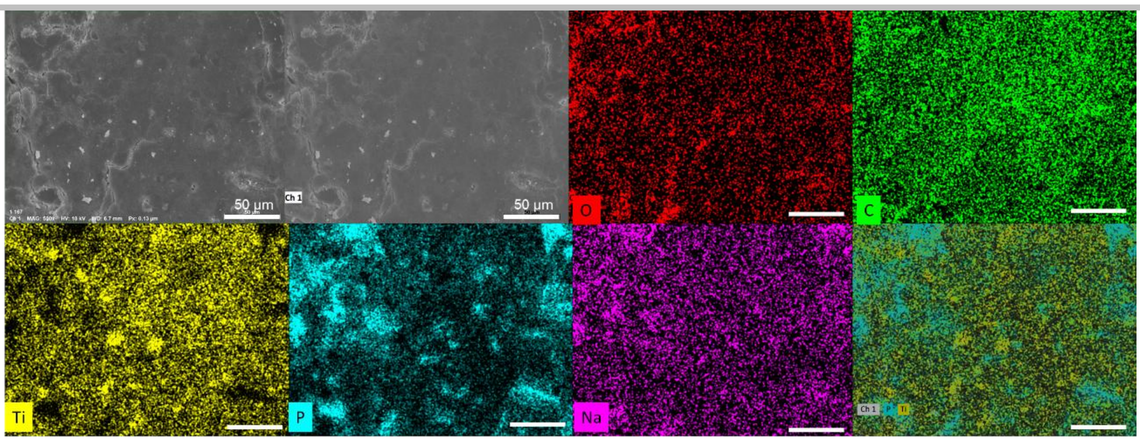

Figure S23. Mapping of the surface of the composite pellet [$(Ti\text{-}MOF)_{0.75}(IG)_{0.25}$]. First and second raw left to right: Image of the analysed area, average image of the analysed area, all individual elements mappings and map generated combining P and Ti elements. Map per element: O (red), C (green), Ti (yellow), P (cyan), Na (pink). Scale 50 µm (white bar).

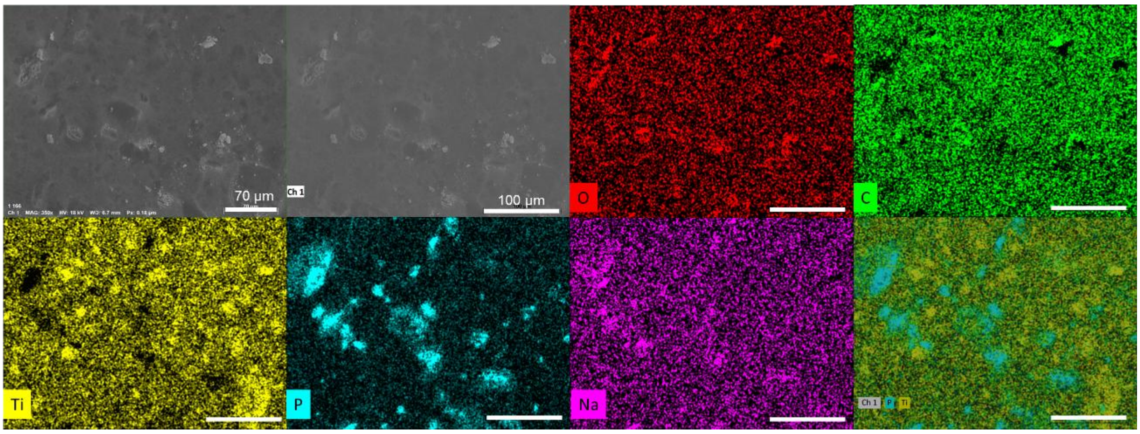

Figure S24. Mapping of the surface of the composite pellet [$(Ti\text{-}MOF)_{0.5}(IG)_{0.5}$]. First and second raw left to right: Image of the analysed area, average image of the analysed area, all individual elements mappings and map generated combining P and Ti elements. Map per element: O (red), C (green), Ti (yellow), P (cyan), Na (pink). Scale 100 µm (white bar).

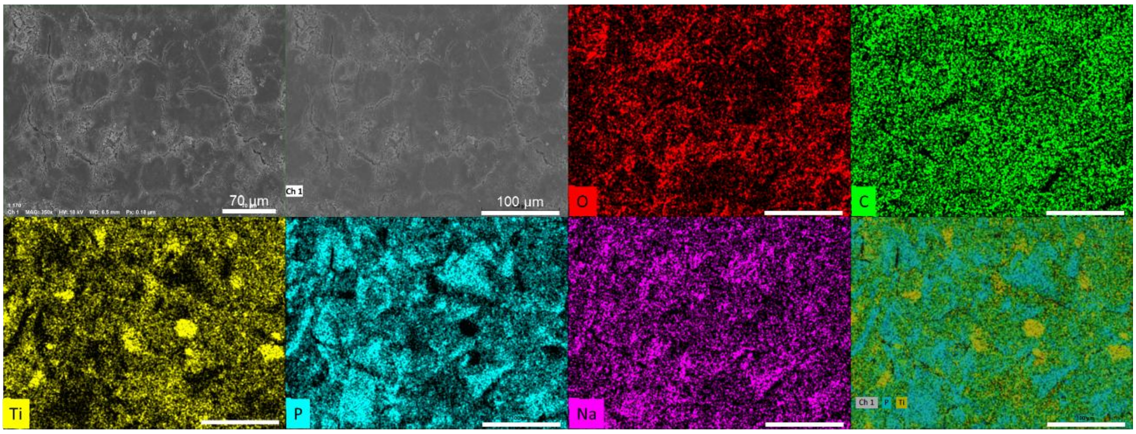

Figure S25. Mapping of the surface of the composite pellet [$(Ti\text{-}MOF)_{0.25}(IG)_{0.75}$]. First and second raw left to right: Image of the analysed area, average image of the analysed area, all individual elements mappings and map generated combining P and Ti elements. Map per element: O (red), C (green), Ti (yellow), P (cyan), Na (pink). Scale 100 µm (white bar).

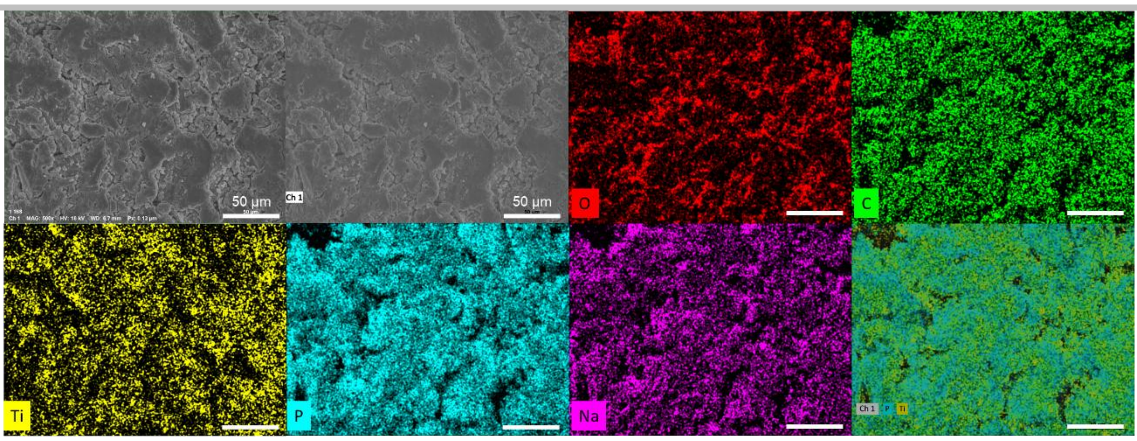

Figure S26. Mapping of the surface of the composite pellet [(Ti-MOF)$_{0.15}$(IG)$_{0.85}$]. First and second raw left to right: Image of the analysed area, average image of the analysed area, all individual elements mappings and map generated combining P and Ti elements. Map per element: O (red), C (green), Ti (yellow), P (cyan), Na (pink). Scale 50 µm (white bar).

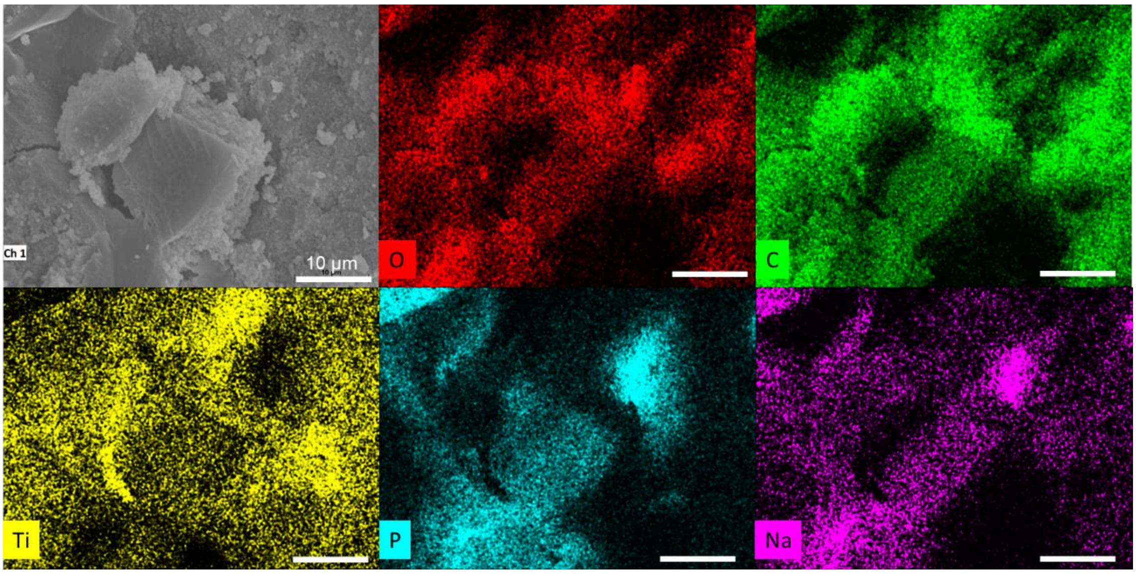

Figure S27. Mapping of inside of the composite pellet [(Ti-MOF)$_{0.75}$(IG)$_{0.25}$]. First and second raw left to right: Image of the analysed area, all individual elements mappings. Map per element: O (red), C (green), Ti (yellow), P (cyan), Na (pink). Scale 10 µm (white bar).

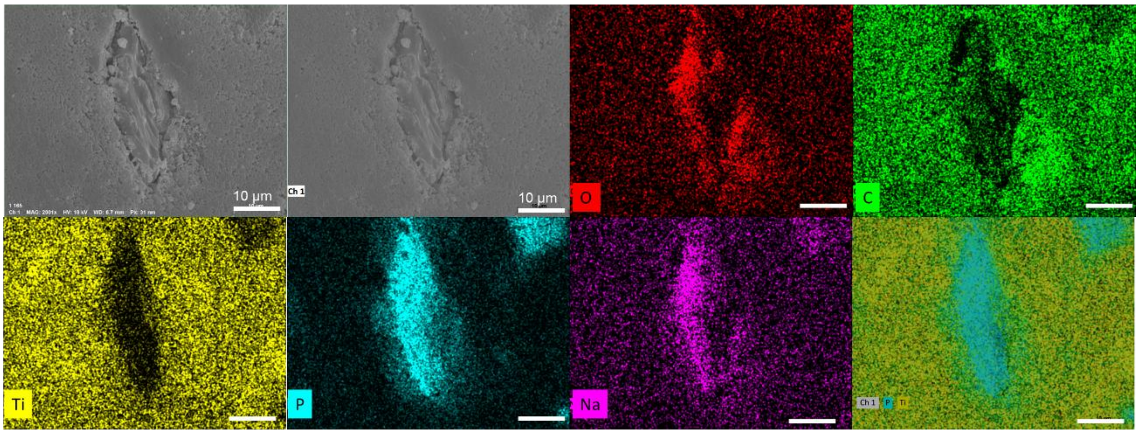

Figure S28. Mapping of inside of the composite pellet [(Ti-MOF)$_{0.5}$(IG)$_{0.5}$]. First and second raw left to right: Image of the analysed area, average image of the analysed area, all individual elements mappings and map generated combining P and Ti elements. Map per element: O (red), C (green), Ti (yellow), P (cyan), Na (pink). Scale 10 µm (white bar).

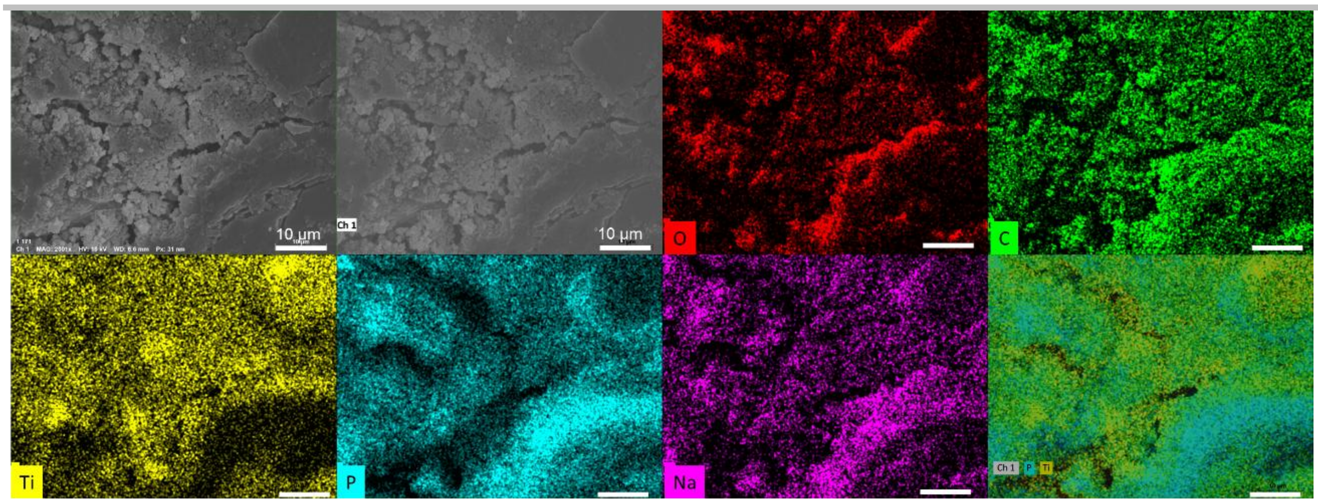

Figure S29. Mapping of inside of the composite pellet [(Ti-MOF)$_{0.25}$(IG)$_{0.75}$]. First and second raw left to right: Image of the analysed area, average image of the analysed area, all individual elements mappings and map generated combining P and Ti elements. Map per element: O (red), C (green), Ti (yellow), P (cyan), Na (pink). Scale 10 µm (white bar).

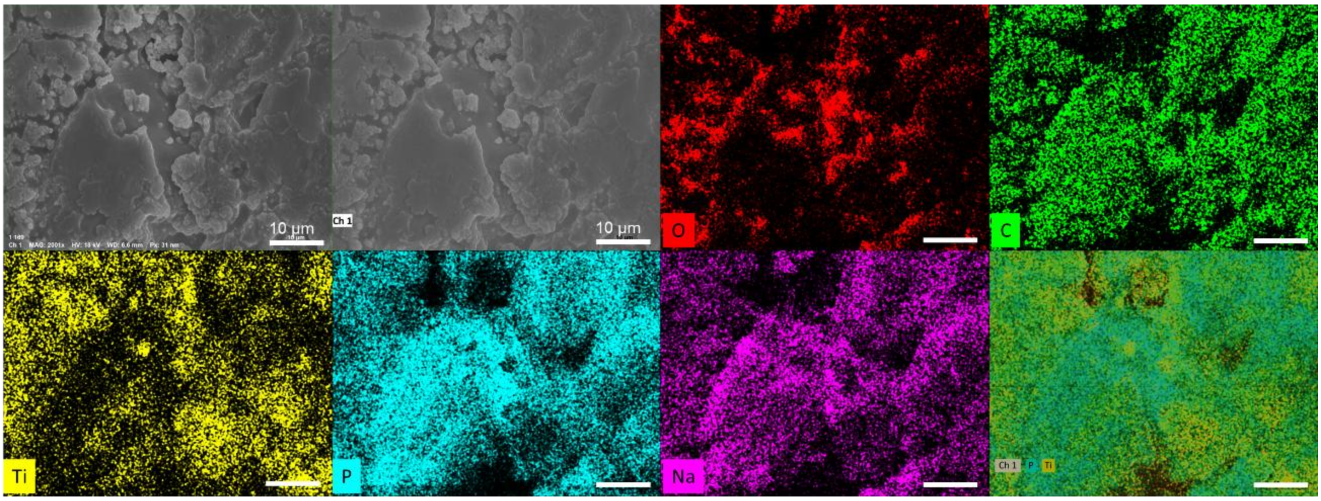

Figure S30. Mapping of inside of the composite pellet [(Ti-MOF)$_{0.15}$(IG)$_{0.85}$]. First and second raw left to right: Image of the analysed area, average image of the analysed area, all individual elements mappings and map generated combining P and Ti elements. Map per element: O (red), C (green), Ti (yellow), P (cyan), Na (pink). Scale 10 µm (white bar).

8. Thermal characterisation

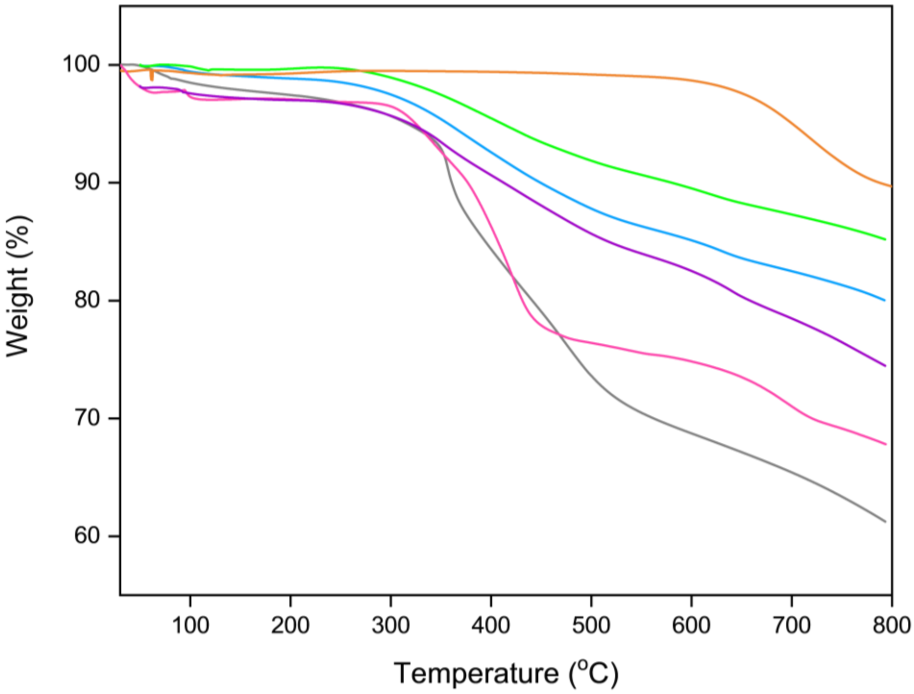

Figure S31. TGAs curves under Ar atmosphere of the inorganic glass (orange), [(Ti-MOF)$_{0.15}$(IG)$_{0.85}$] composite (green), [(Ti-MOF)$_{0.25}$(IG)$_{0.75}$] composite (cyan), [(Ti-MOF)$_{0.50}$(IG)$_{0.50}$] composite (purple), [(Ti-MOF)$_{0.75}$(IG)$_{0.25}$] composite (pink) and the activated pristine Ti-MOF (grey). Heating rate 10 ºC/min.

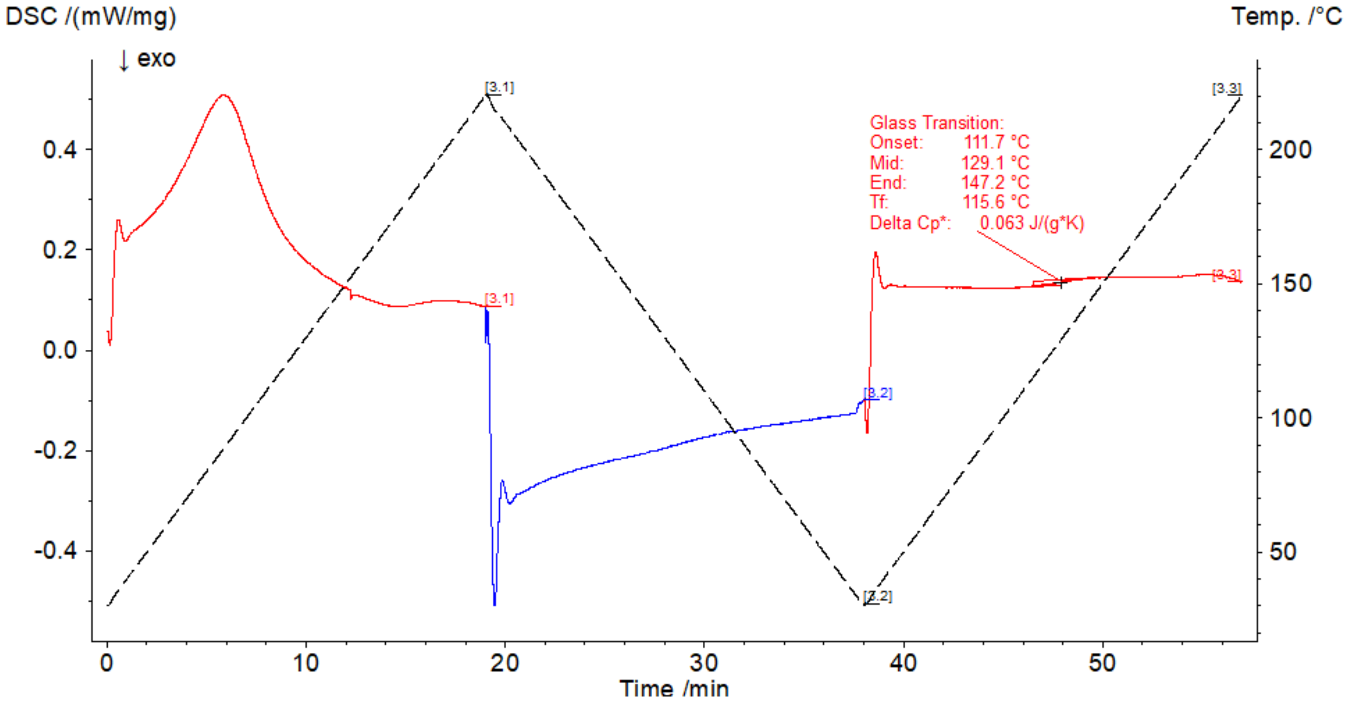

Figure S32. DSC of (Ti-MOF)$_{0.15}$/(IG)$_{0.85}$ physical mixture performed at a maximum temperature of 220 ºC for 2 heat/cool cycles under an argon atmosphere with a heating/cooling rate of 10 ºC/min. BPM

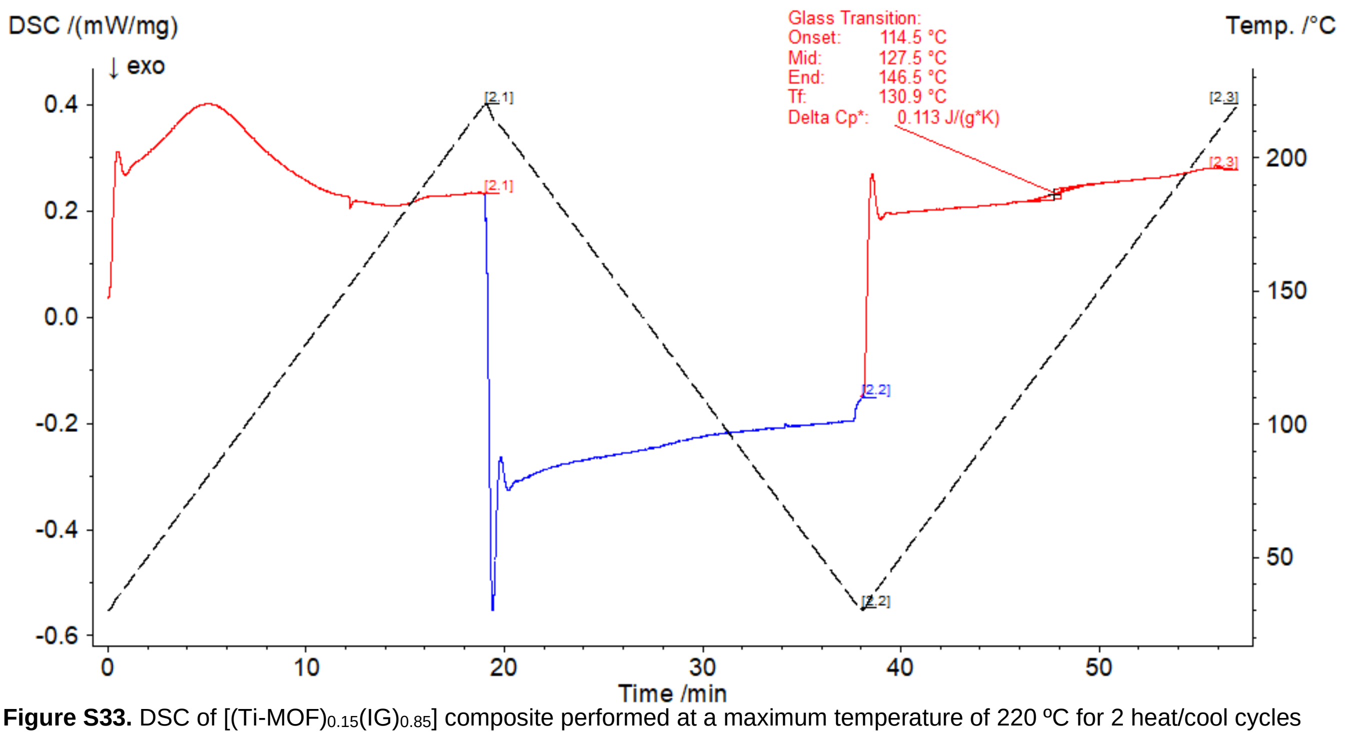

Figure S33. DSC of [(Ti-MOF)$_{0.15}$(IG)$_{0.85}$] composite performed at a maximum temperature of 220 ºC for 2 heat/cool cycles under an argon atmosphere with a heating/cooling rate of 10 ºC/min.

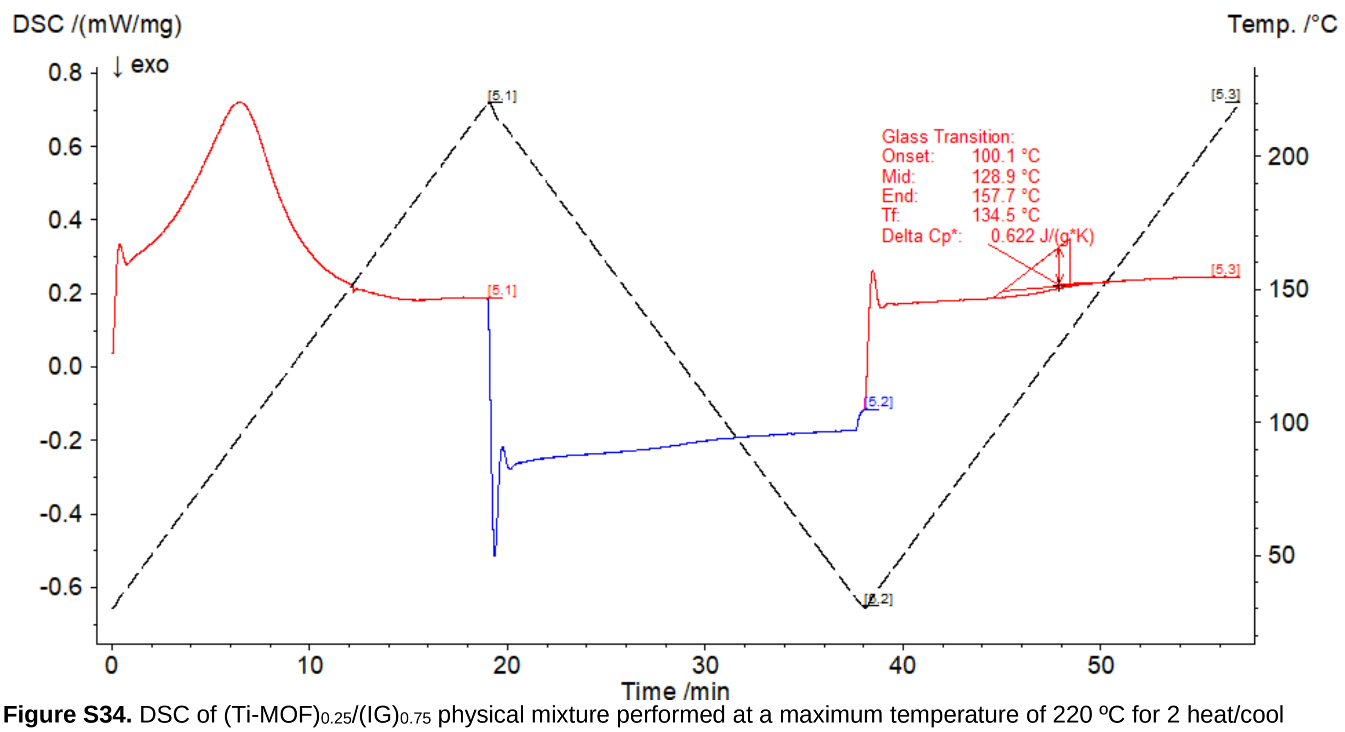

Figure S34. DSC of (Ti-MOF)$_{0.25}$/(IG)$_{0.75}$ physical mixture performed at a maximum temperature of 220 ºC for 2 heat/cool cycles under an argon atmosphere with a heating/cooling rate of 10 ºC/min.

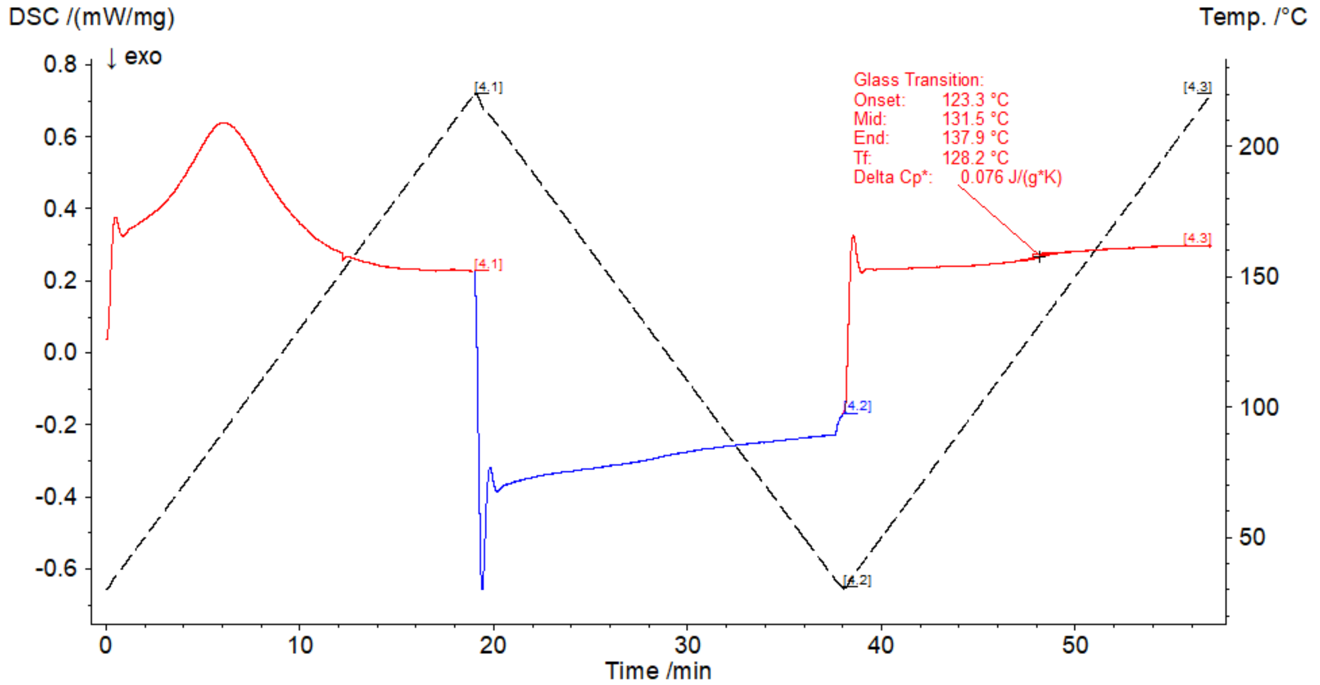

Figure S35. DSC of [(Ti-MOF)$_{0.25}$(IG)$_{0.75}$] composite performed at a maximum temperature of 220 ºC for 2 heat/cool cycles under an argon atmosphere with a heating/cooling rate of 10 ºC/min.

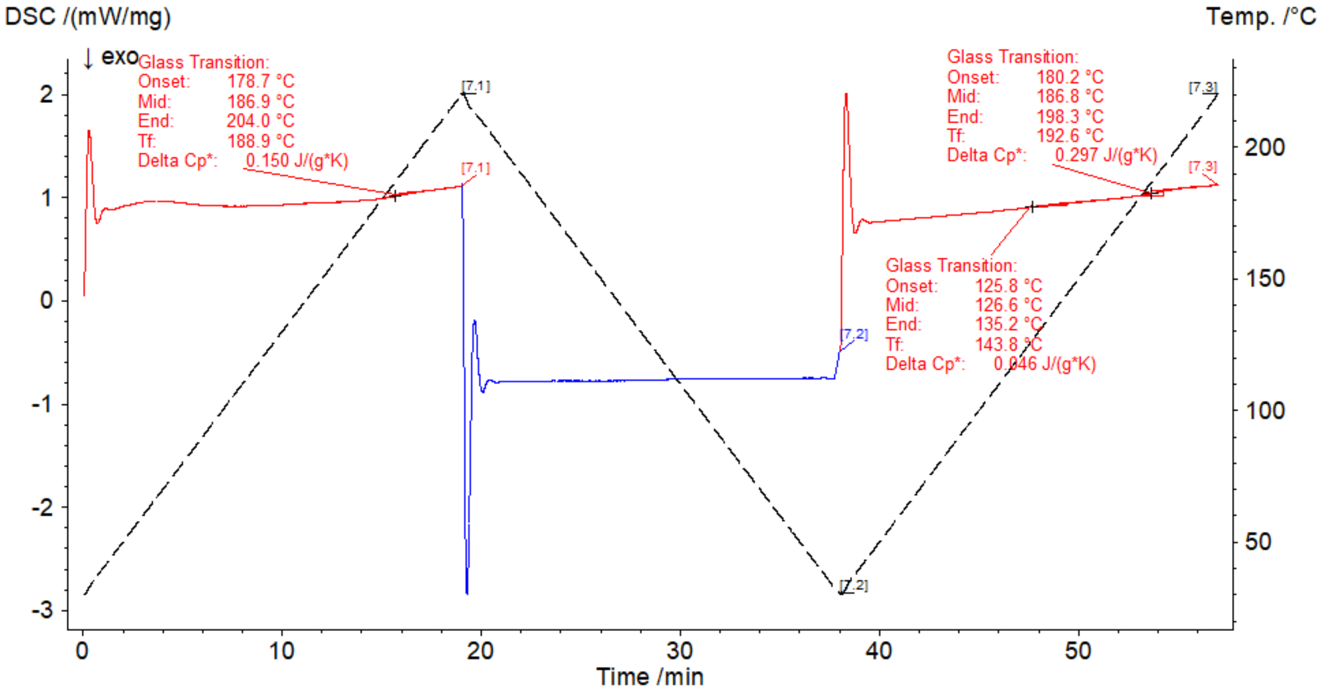

Figure S36. DSC of (Ti-MOF)$_{0.50}$/(IG)$_{0.50}$ physical mixture performed at a maximum temperature of 220 ºC for 2 heat/cool cycles under an argon atmosphere with a heating/cooling rate of 10 ºC/min.

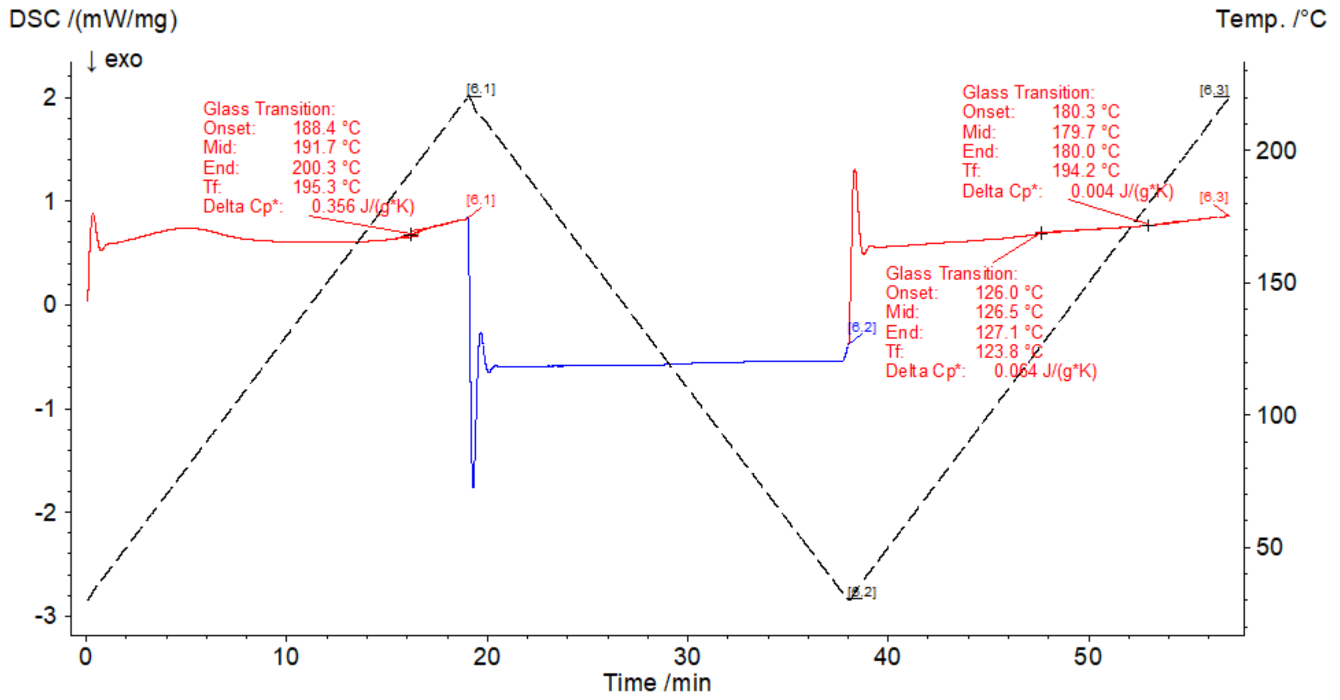

Figure S37. DSC of [(Ti-MOF)$_{0.50}$(IG)$_{0.50}$] composite performed at a maximum temperature of 220 ºC for 2 heat/cool cycles under an argon atmosphere with a heating/cooling rate of 10 ºC/min.

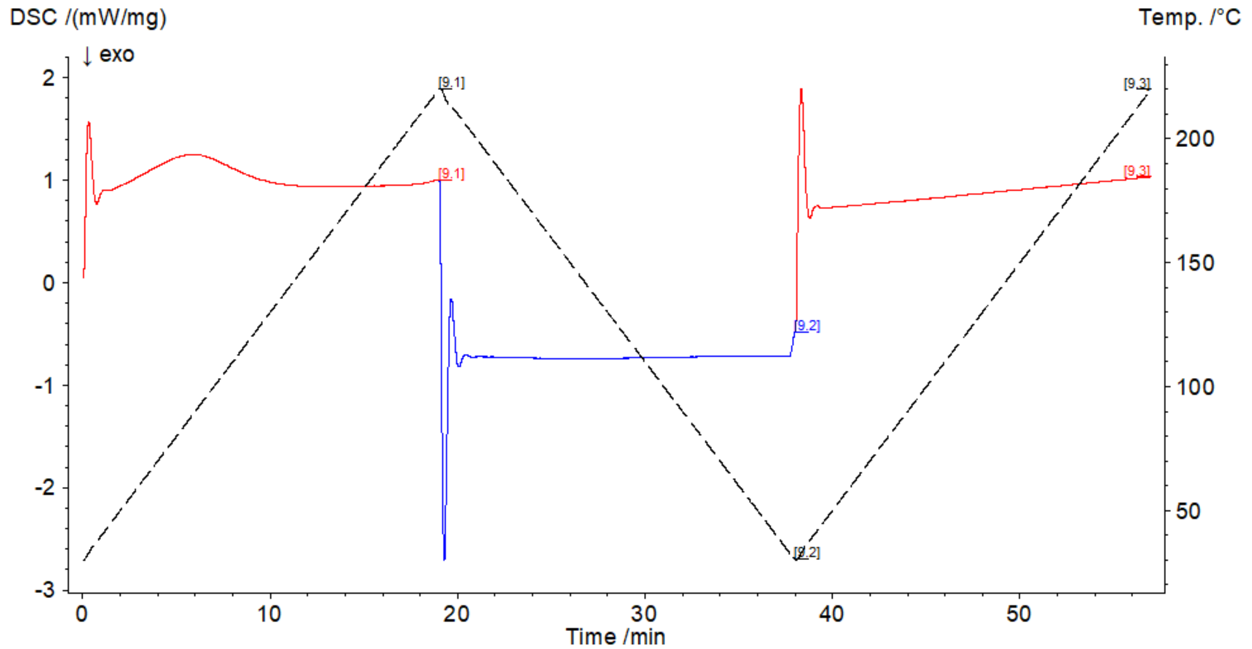

Figure S38. DSC of (Ti-MOF)$_{0.75}$/(IG)$_{0.25}$ physical mixture performed at a maximum temperature of 220 ºC for 2 heat/cool cycles under an argon atmosphere with a heating/cooling rate of 10 ºC/min.

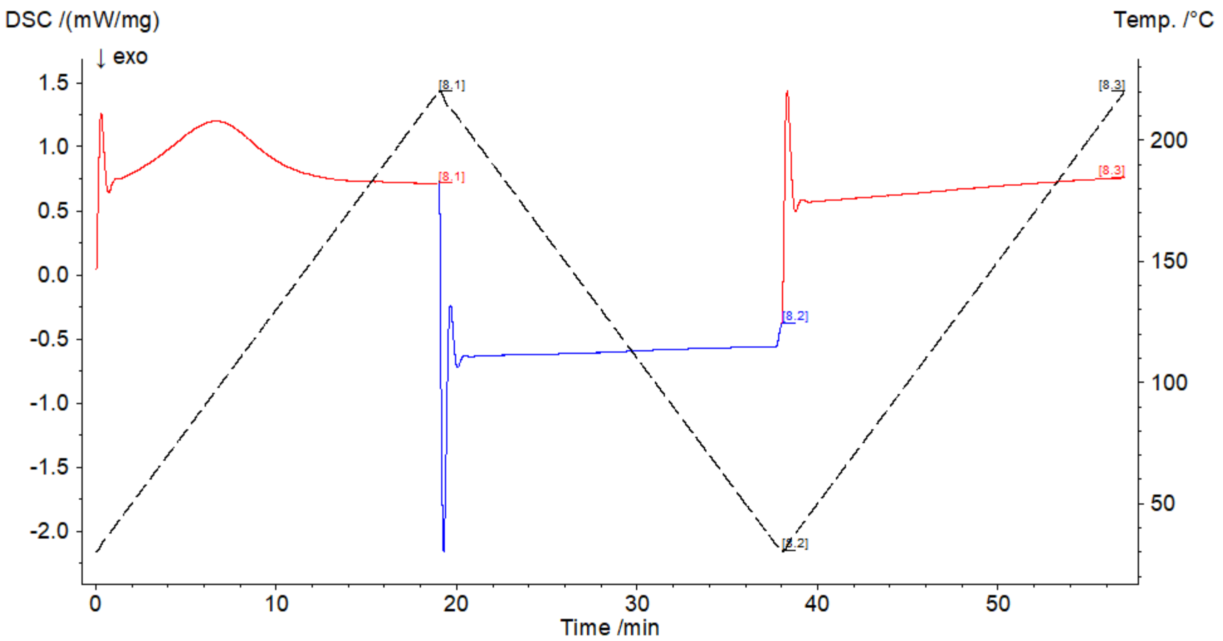

Figure S39. DSC of [(Ti-MOF)$_{0.75}$(IG)$_{0.25}$] composite performed at a maximum temperature of 220 ºC for 3 heat/cool cycles under an argon atmosphere with a heating/cooling rate of 10 ºC/min.

9. Pair distribution function study

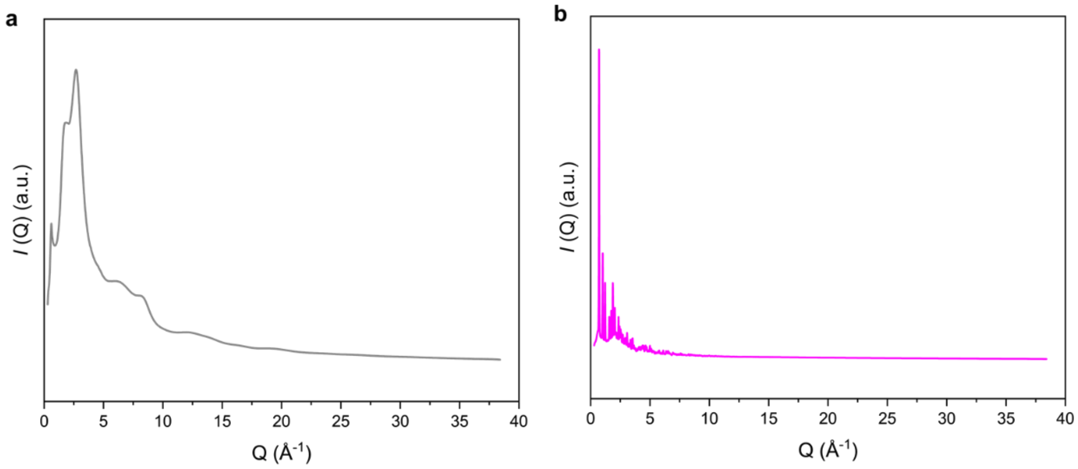

Figure S40. Measured diffraction data *I*(Q) of **a**. the inorganic glass and **b**. of the Ti-MOF.

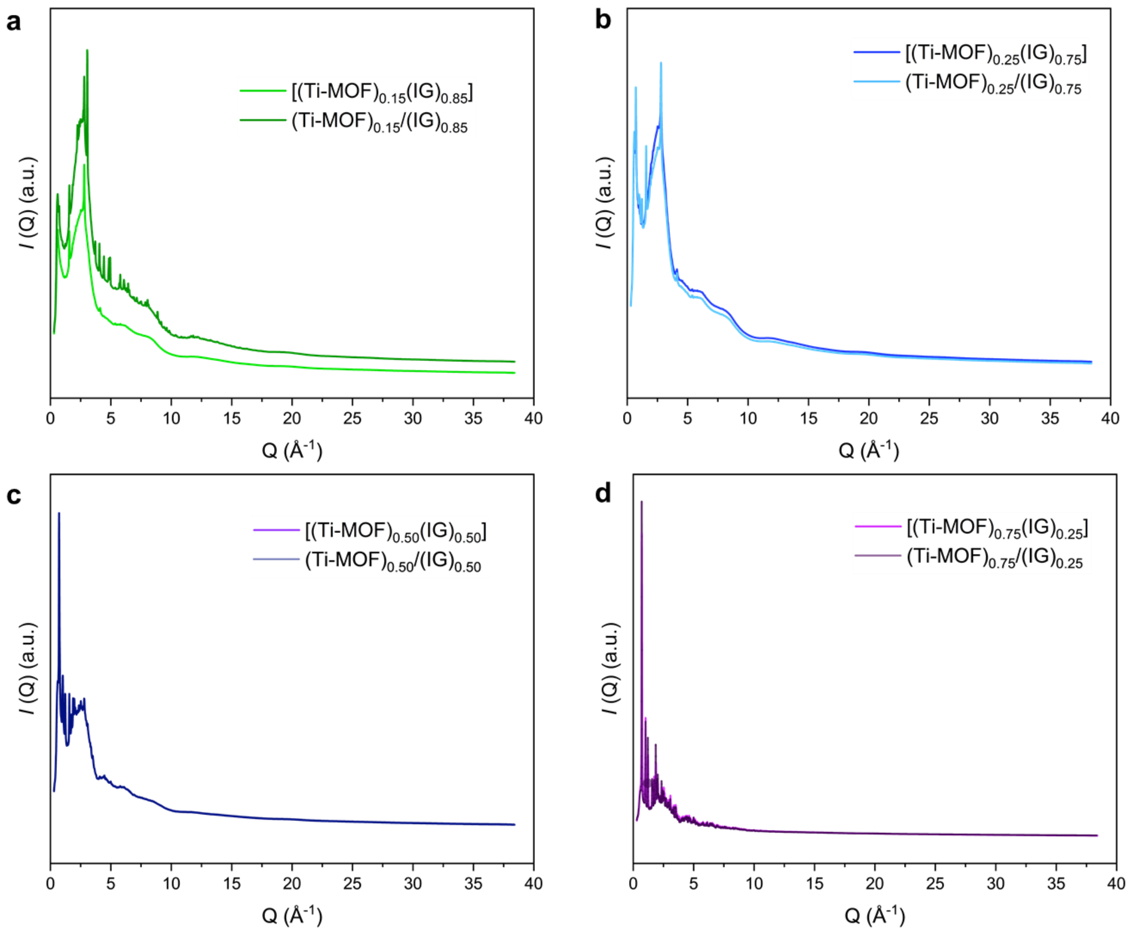

Figure S41. I(Q) of all the composites and physical mixtures. **a**. *I*(Q) of the composite and physical mixture containing a 15% weight of Ti-MOF. **b**. *I*(Q) of the composite and physical mixture containing a 25% weight of Ti-MOF. **c**. *I*(Q) of the composite and physical mixture containing a 50% weight of Ti-MOF. **d**. *I*(Q) of the composite and physical mixture containing a 75% weight of Ti-MOF.

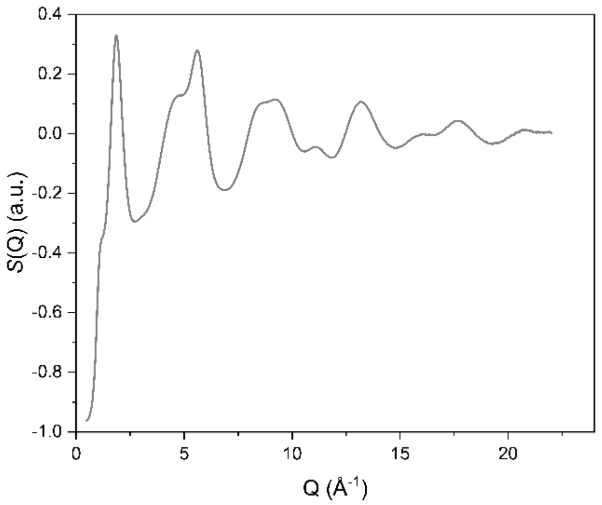

Figure S42. Normalised total scattering structure factor S(Q) of the inorganic glass.

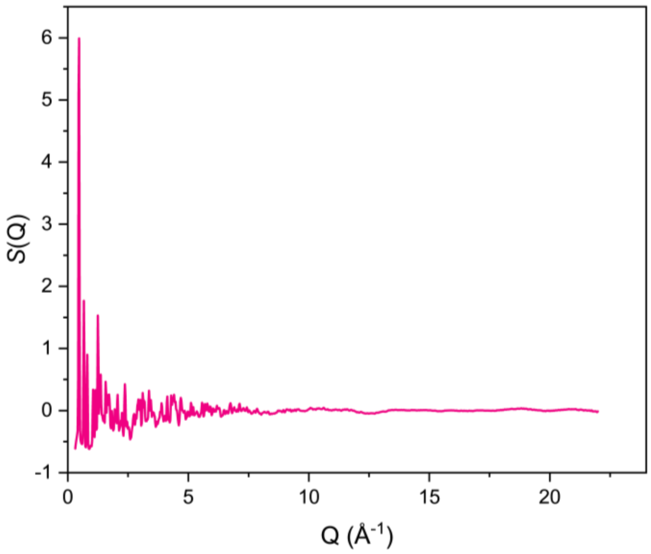

Figure S43. S(Q) of the Ti-MOF.

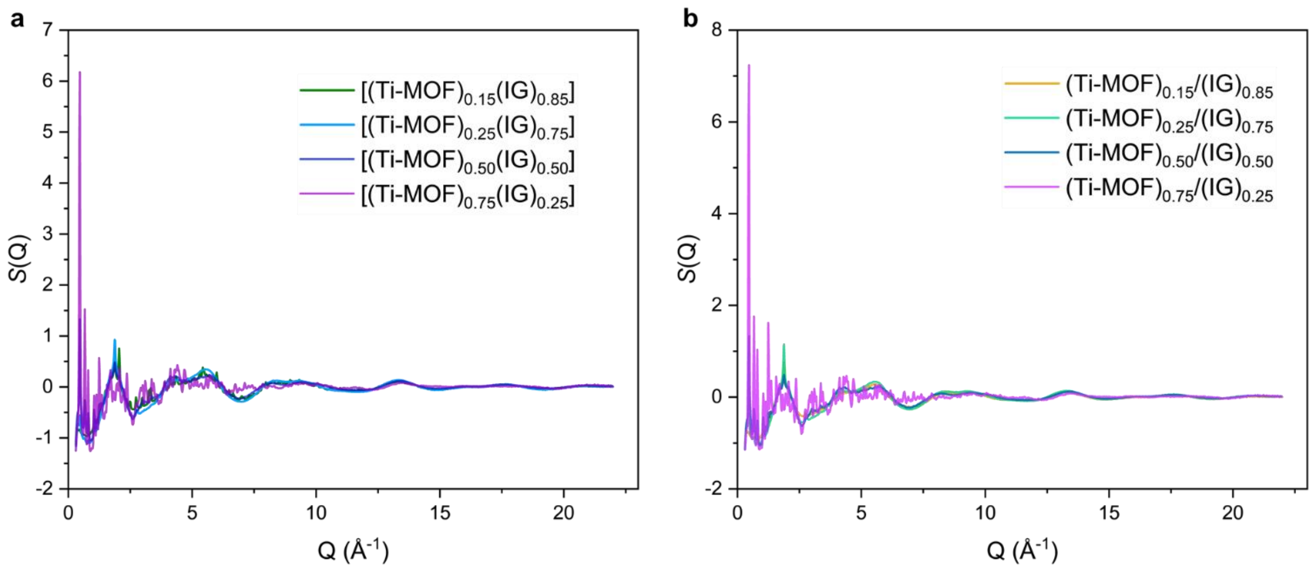

Figure S44. S(Q) of the **a**. composites and **b**. physical mixtures.

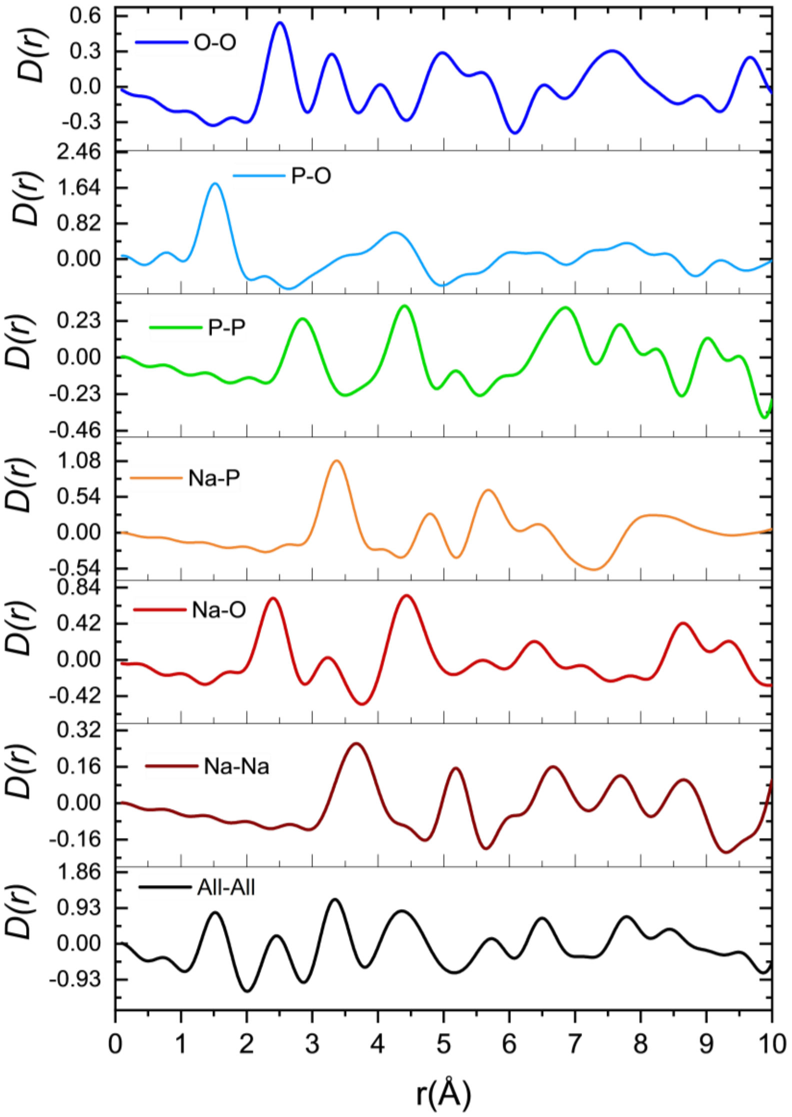

Figure S45. Calculated total and partial X-ray pair distribution functions g(r) of the crystalline phase $Na_3P_3O_9$ using PDFGUI (COD: 2310645).

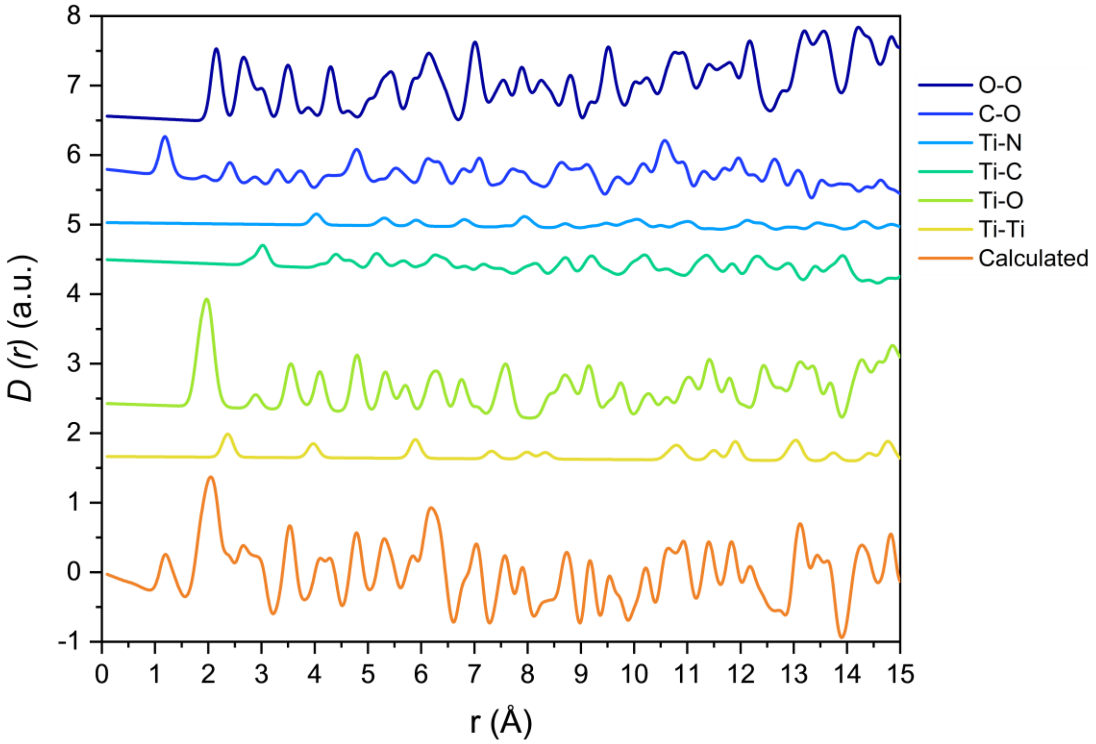

Figure S46. Calculated total and partial X-ray pair distribution function g(r) of the Ti-MOF (MIL-125-NH_2) using PDFGUI. Modified from CCDC: 1527305 using Materials Studio.

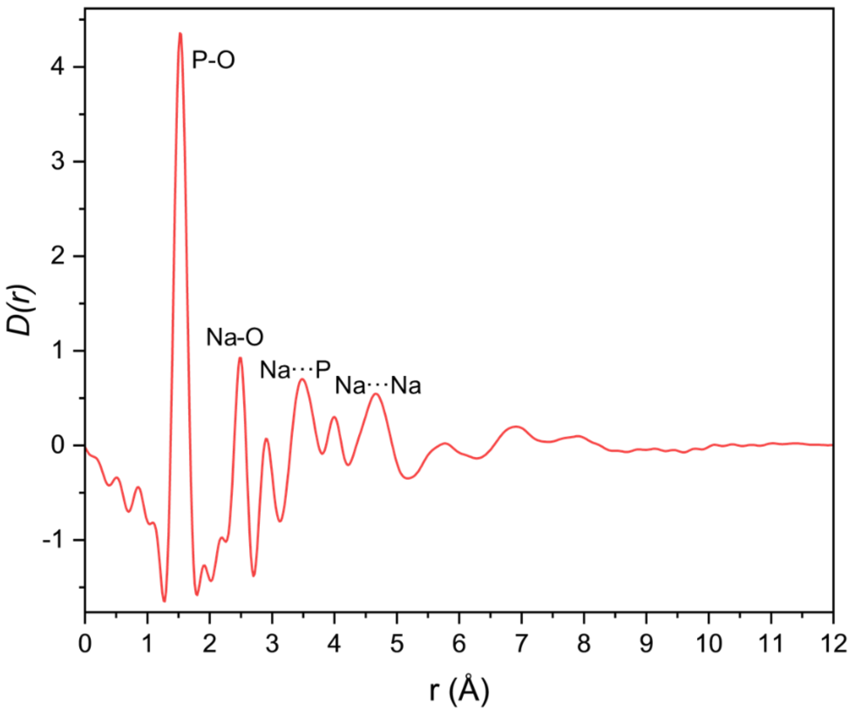

Figure S47. X-ray pair distribution function *D*(r) of the $20(Na_2O)10(Na_2SO_4)70(P_2O_5)$ glass (IG) plotted from 1-12 Å to show short-range order and principal contributions.

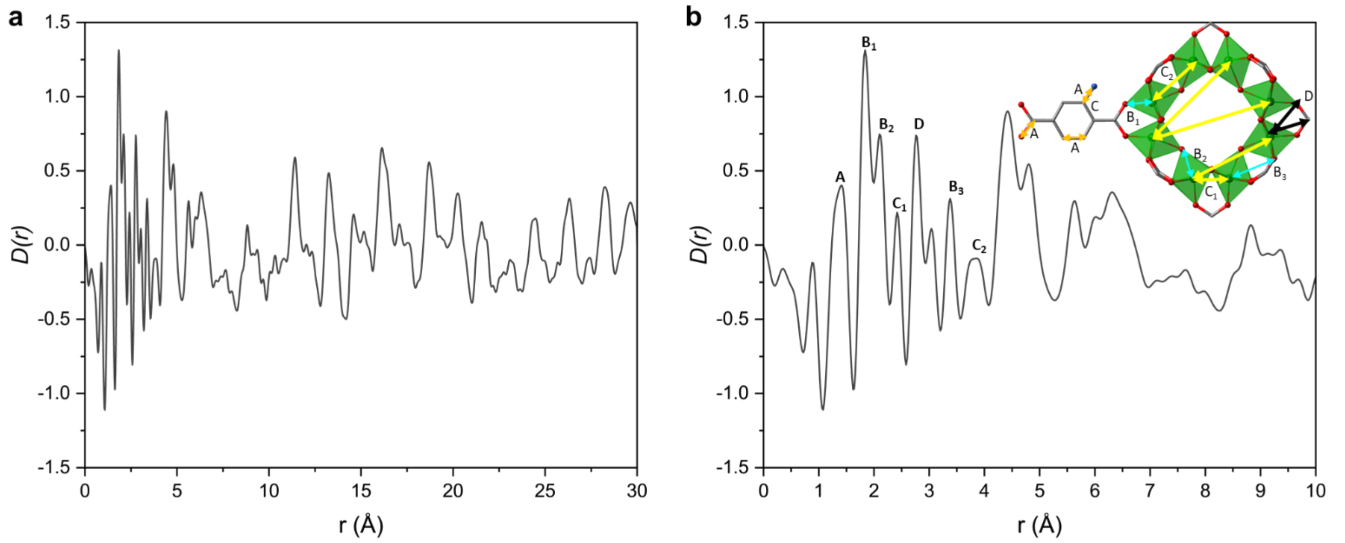

Figure S48. a. X-ray pair distribution function *D*(r) of Ti-MOF (MIL-125-NH_2). **b**. The same *D*(r) plotted from 0-10 Å to show short-range order and principal contributions. Titanium (green), nitrogen (blue), carbon (grey), oxygen (red).

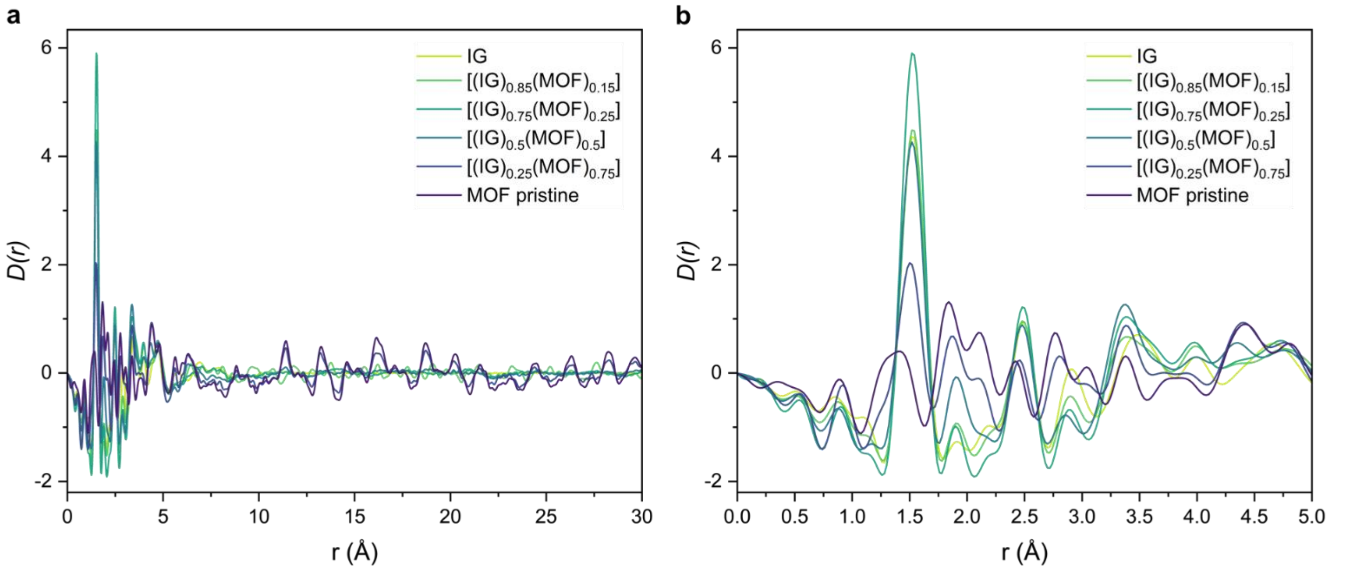

Figure S49. a. *D*(r) of the composites. **b**. *D*(r) of composites plotted from 0-5 Å to show short-range order and principal contributions of the inorganic glass and the Ti-MOF.

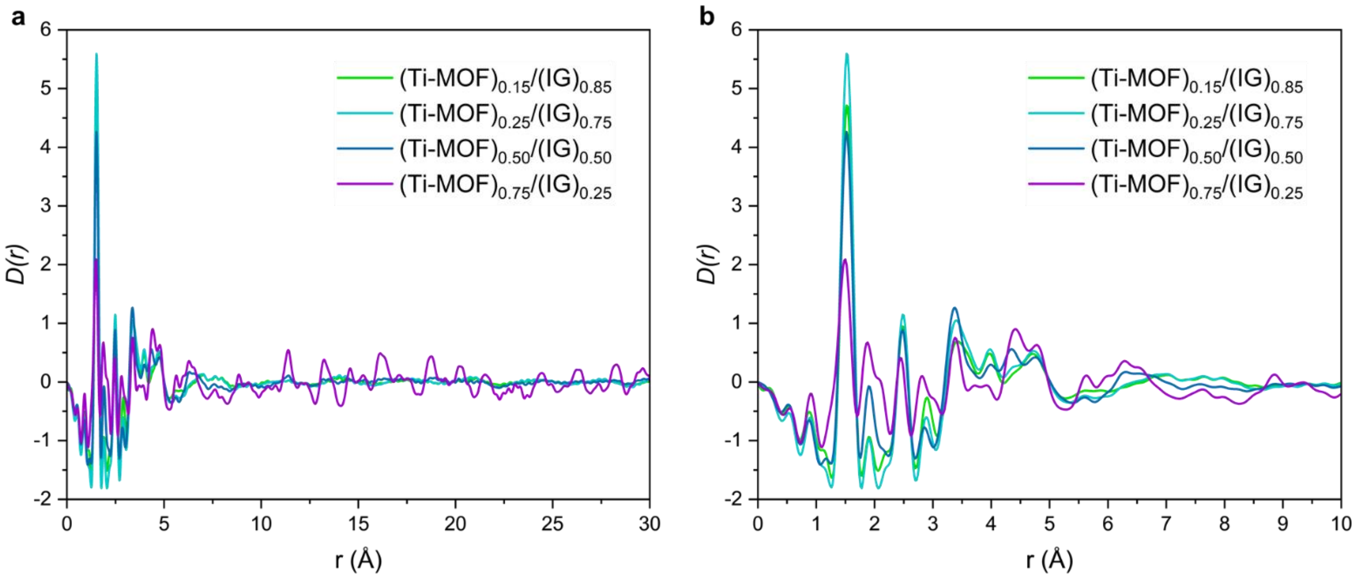

Figure S50. a. D(r) of the physical mixtures. **b**. D(r) of physical mixture plotted from 1-10 Å to show short-range order and principal contributions of the inorganic glass and the Ti-MOF.

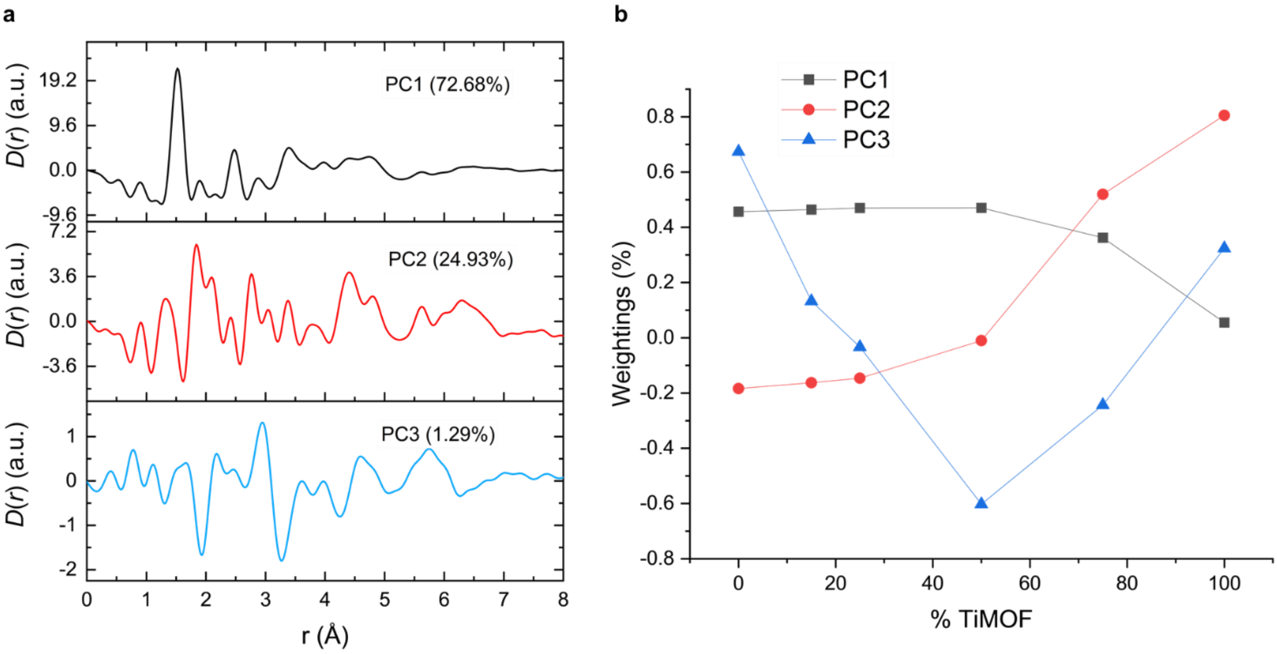

Figure S51. **a**. Extracted principal components (PCs) from the PCA. **b**. The weightings of the extracted PCs obtained for each sample.

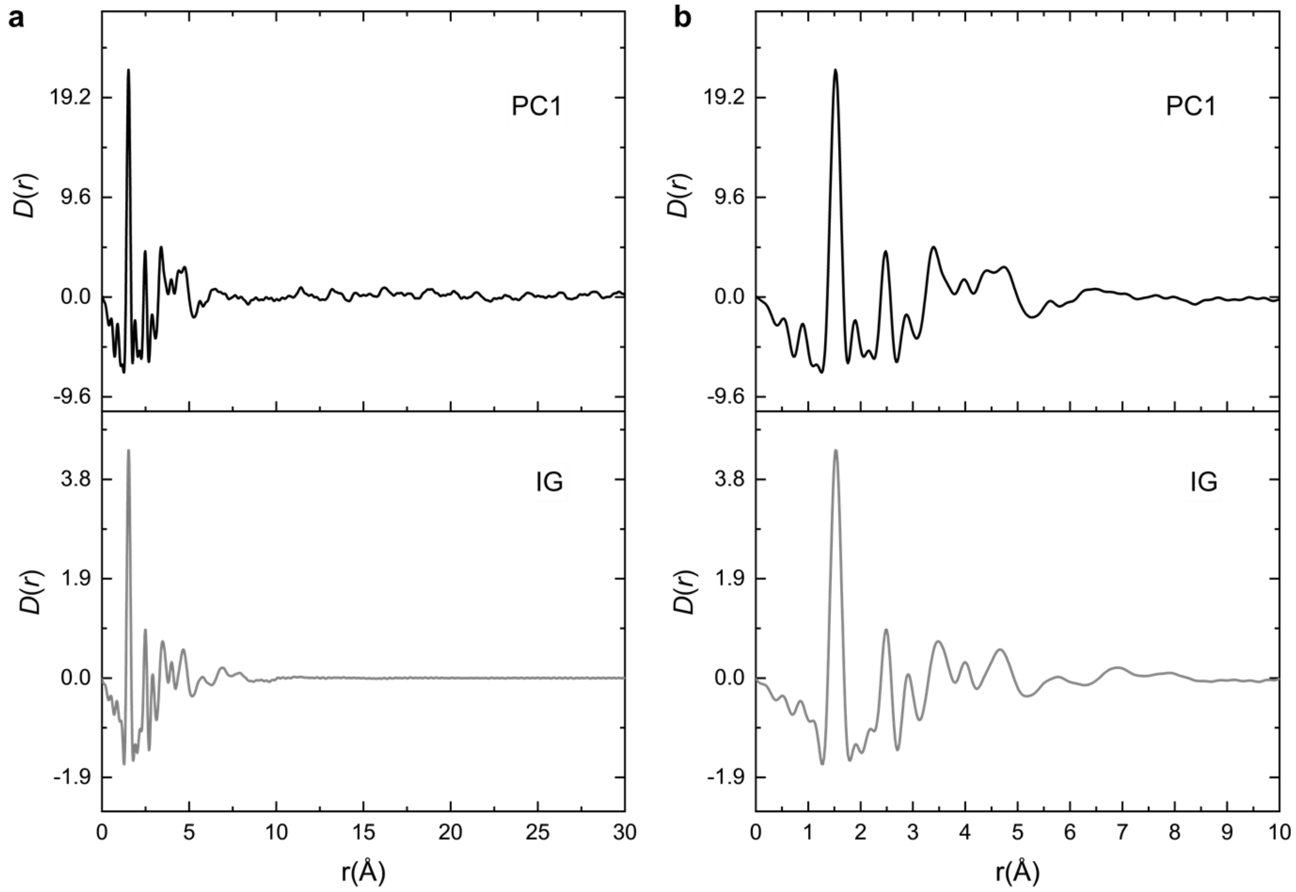

Figure S52. **a**.PC1 and *D*(r) of the inorganic glass (IG). **b**. The same functions plotted over a shorter range (0-10 Å) showing their similar correlations over these distances.

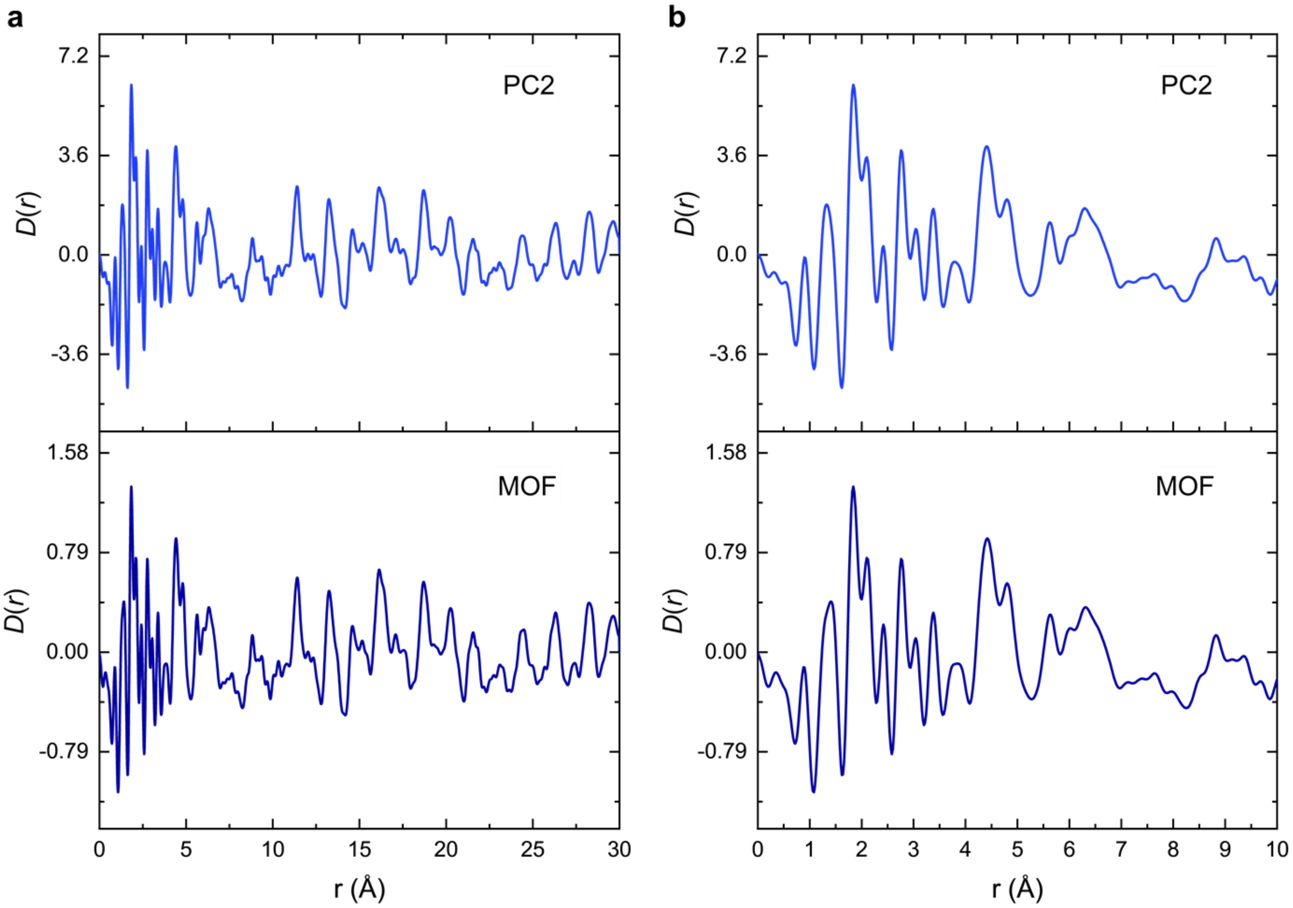

Figure S53. a. *D*(r) of the PC2 and Ti-MOF. **b**. *D*(r) plotted at short range (0-10 Å) showing same correlations.

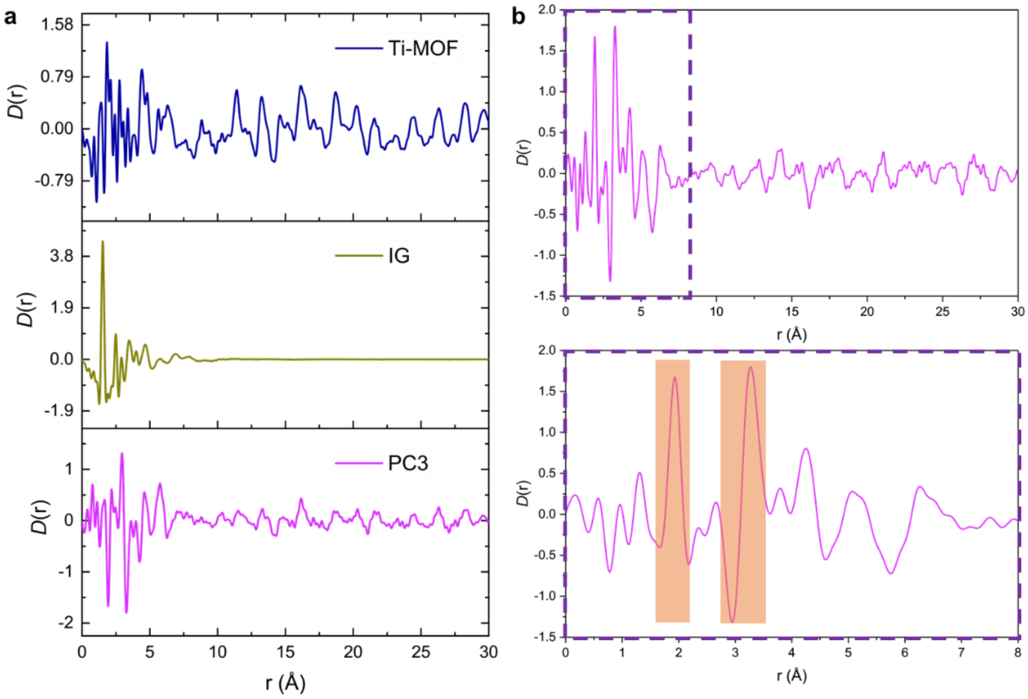

Figure S54. a. *D*(r) of the PC3 compared to pristine Ti-MOF and IG. **b**. –PC3 function highlighting 2 main contributions.

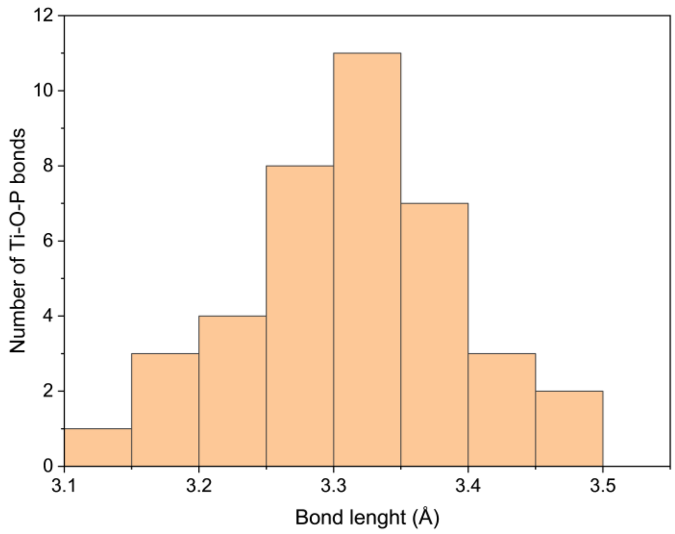

Figure S55. Distances between Ti and P for multiple materials containing Ti-O-P correlations. Employed CODs: 8103856, 7707163, 7119534, 4315114, 2011792, 2010155, 2002999, 1001530.

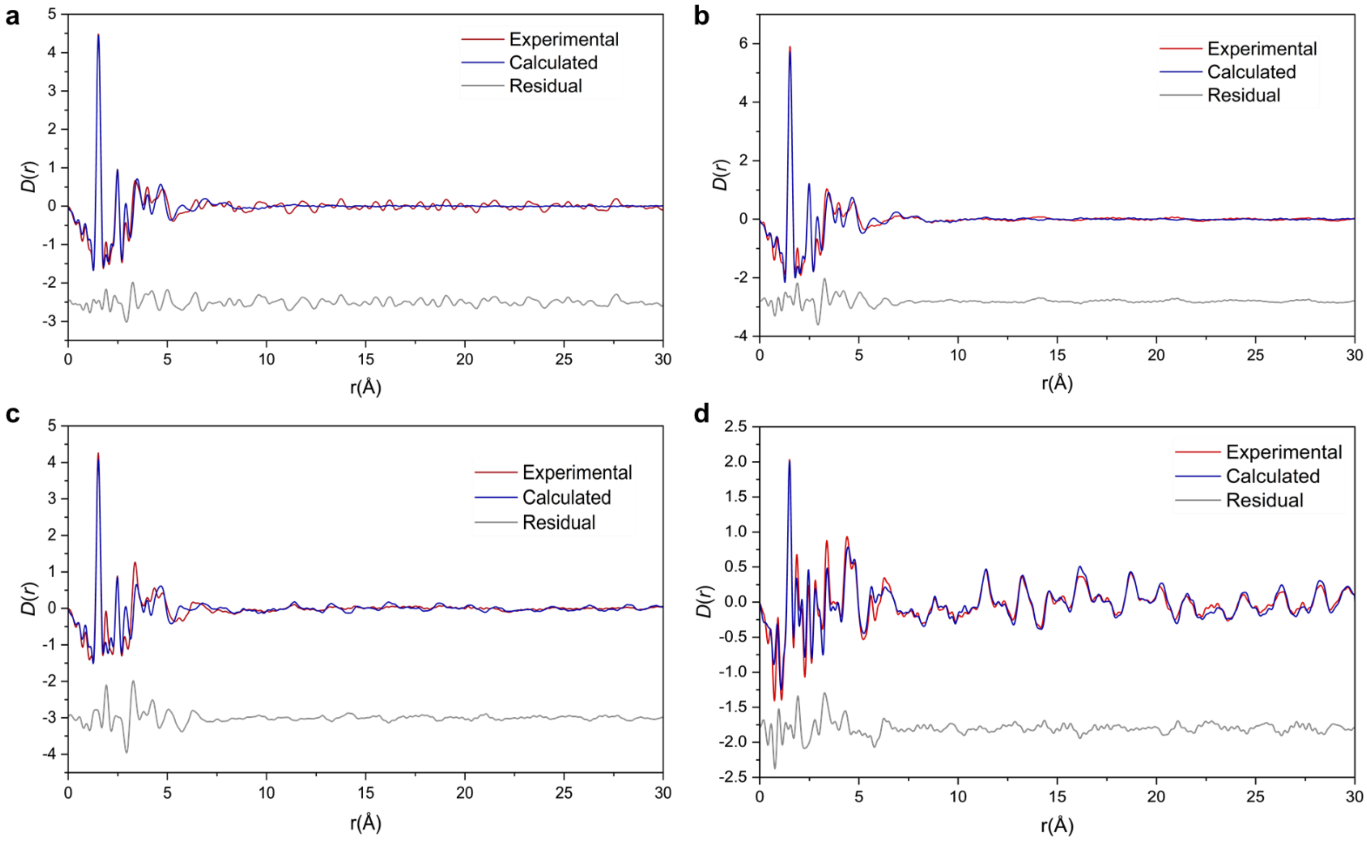

Figure S56. Fits from multilinear regression analysis of composites containing different proportions of Ti-MOF. **a**. [(Ti-MOF)$_{0.15}$(IG)$_{0.85}$], **b**. [(Ti-MOF)$_{0.25}$(IG)$_{0.75}$], **c**. [(Ti-MOF)$_{0.50}$(IG)$_{0.50}$] and **d**. [(Ti-MOF)$_{0.75}$(IG)$_{0.25}$].

Table S5. Fitting results. R^2-value of the end members is 1, due to the perfect fit with one of the components. However, for the intermediate members, we are using a two components model to fit what we believe is a three-component system: MOF-Inorganic Glass-interface. Therefore, the R-values for intermediate members of the series would be expected to decrease. They also suggest the deviation from the two-component model is greatest for the composite containing a 50% of Ti-MOF. This is perhaps the sample that maximizes the interaction between phases. We can visualize this change in the R-value through the residuals, that are left over after regression. R-values of 1 for end members mean zero residual, and consequently a flat line. Residuals in the intermediate compositions are features that cannot be accounted for using Ti-MOF or the inorganic glass, these represent the minimum difference as some of the interaction PDF features will have been fitted by the Ti-MOF/IG.

Regression coefficients	[(Ti-MOF)$_{0.15}$(IG)$_{0.85}$]	[(Ti-MOF)$_{0.25}$(IG)$_{0.75}$]	[(Ti-MOF)$_{0.50}$(IG)$_{0.50}$]	[(Ti-MOF)$_{0.75}$(IG)$_{0.25}$]
A	1.020(7)	1.315(8)	0.9419(99)	0.471(6)
B	0.031(10)	0.067(11)	0.264(14)	0.7798(86)
C	0	0	0	0
R^2	0.930(114)	0.946(127)	0.857(159)	0.896(97)

10. X-ray photoelectron spectroscopy (XPS)

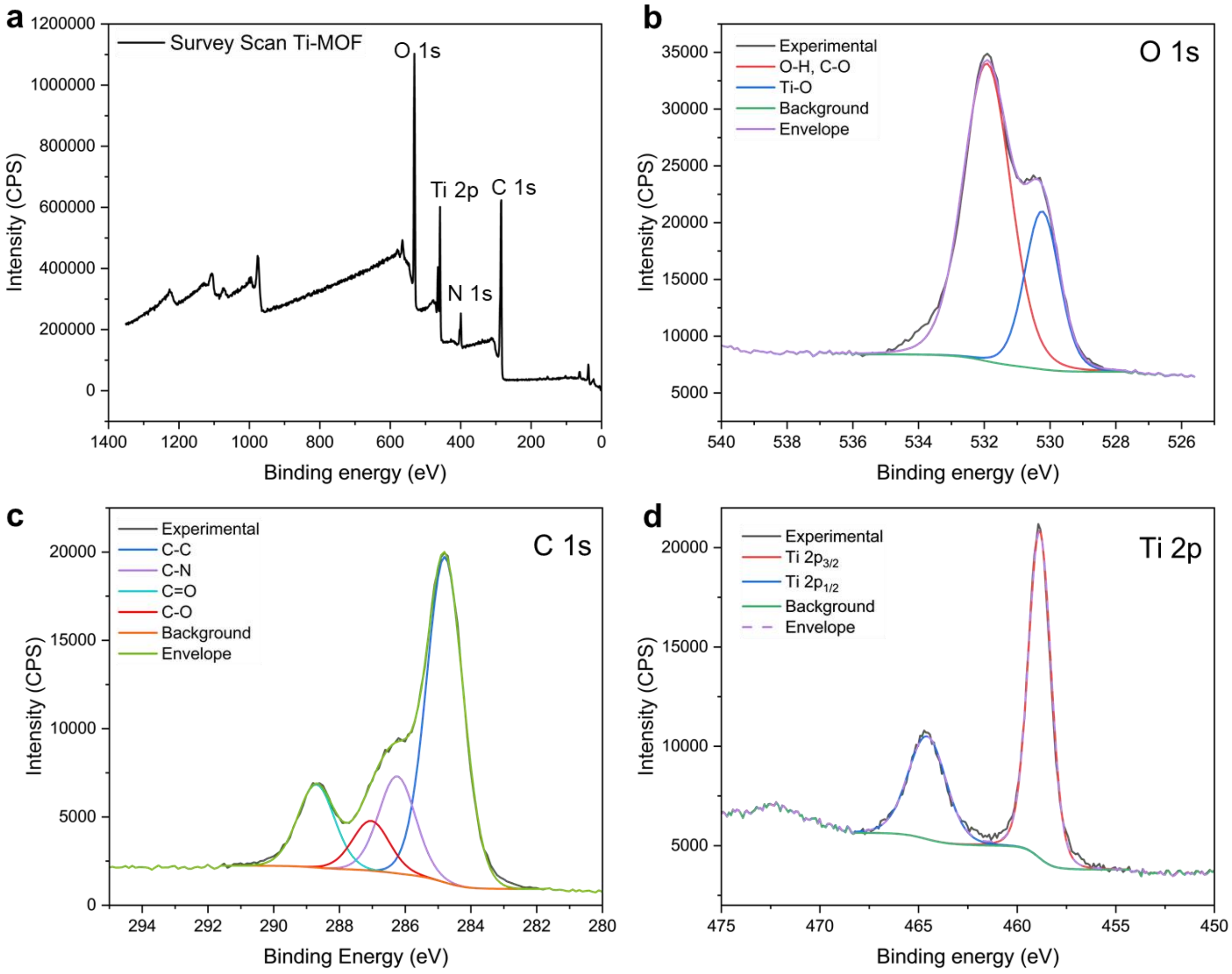

Figure S57. XPS spectra of the Ti-MOF pristine. **a**. Survey scan with main peaks labelled. **b**. O1s spectrum with deconvoluted contributions labelled. **c**.C1s spectrum with deconvoluted contributions labelled. **d**. Ti2p spectrum with deconvoluted contributions labelled.

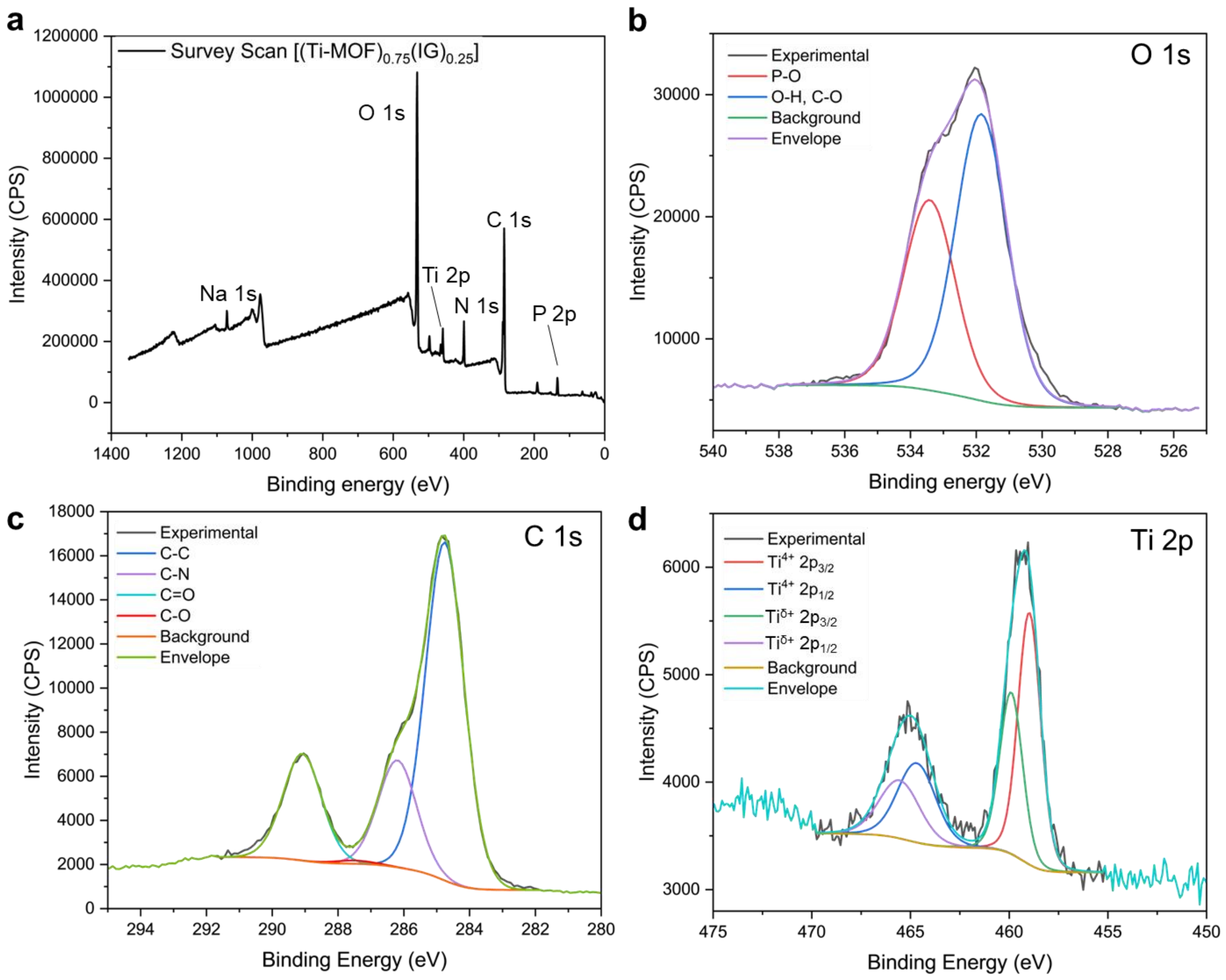

Figure S58. XPS spectra of the [(Ti-MOF)$_{0.75}$(IG)$_{0.25}$] composite. **a**. Survey scan with main peaks labelled. **b**. O1s spectrum with deconvoluted contributions labelled. **c**.C1s spectrum with deconvoluted contributions labelled. **d**. Ti2p spectrum with deconvoluted contributions labelled.

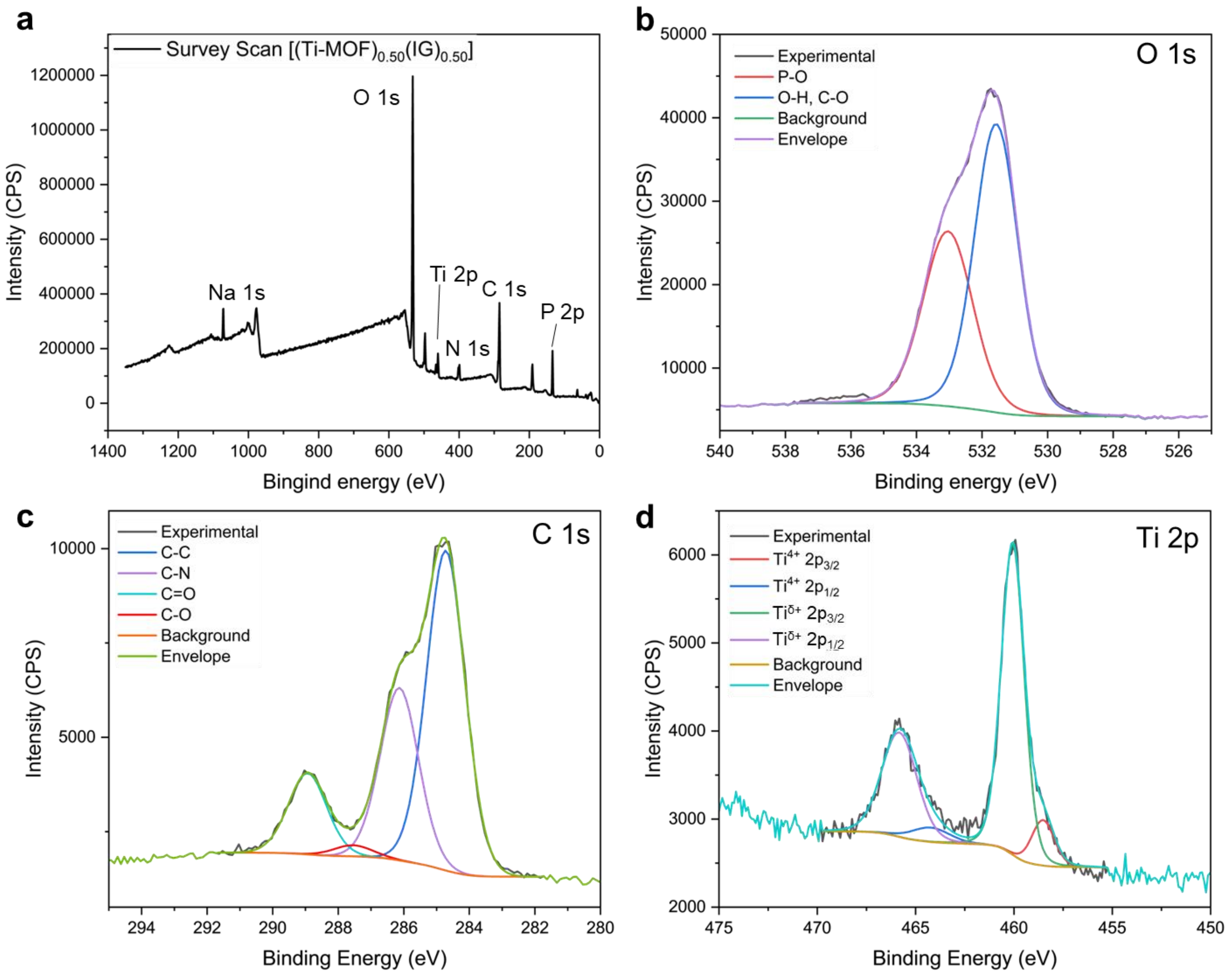

Figure S59. XPS spectra of the [(Ti-MOF)$_{0.50}$(IG)$_{0.50}$] composite. **a**. Survey scan with main peaks labelled. **b**. O1s spectrum with deconvoluted contributions labelled. **c**.C1s spectrum with deconvoluted contributions labelled. **d**. Ti2p spectrum with deconvoluted contributions labelled.

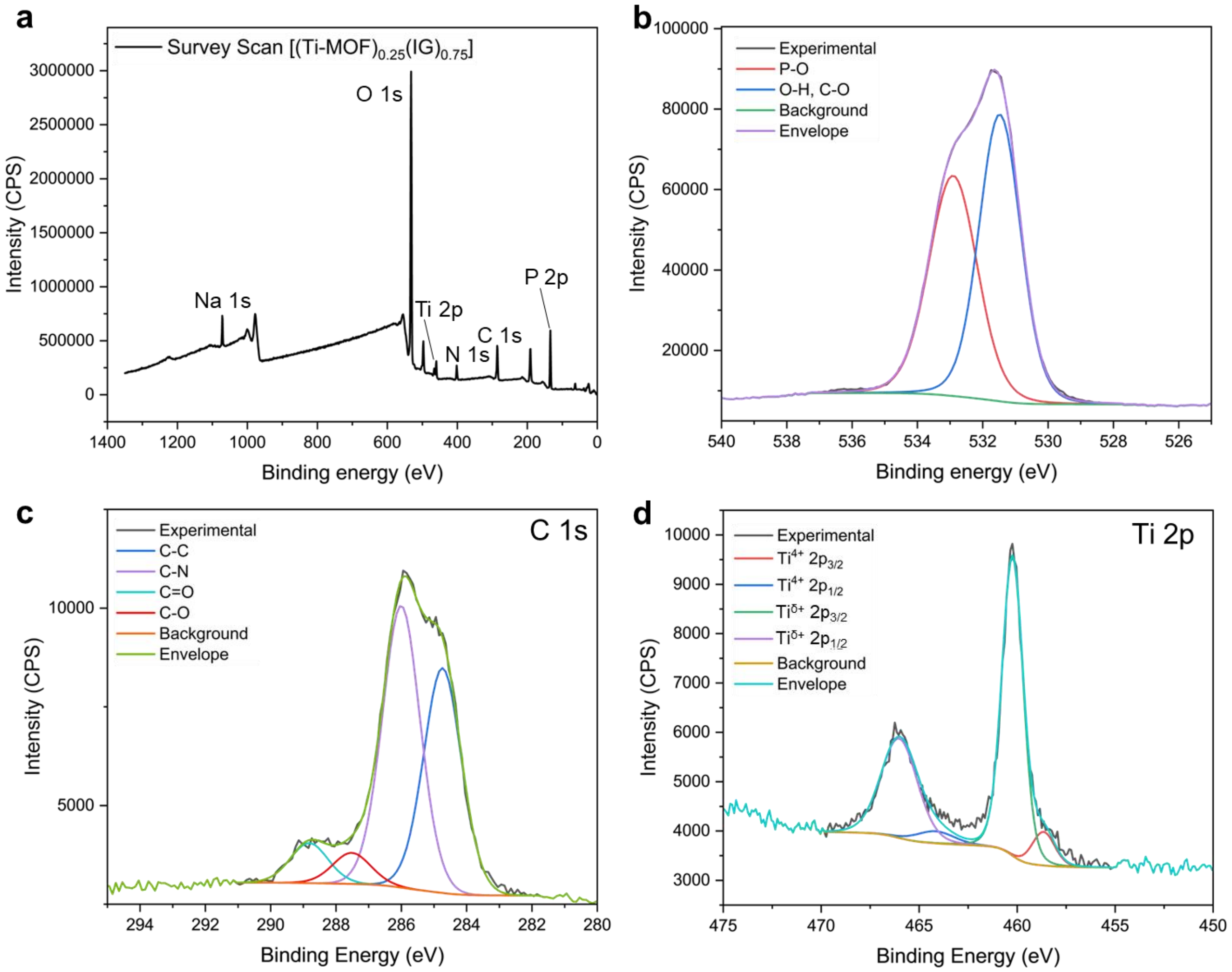

Figure S60. XPS spectra of the [$(Ti\text{-}MOF)_{0.25}(IG)_{0.75}$] composite. **a**. Survey scan with main peaks labelled. **b**. O1s spectrum with deconvoluted contributions labelled. **c**.C1s spectrum with deconvoluted contributions labelled. **d**. Ti2p spectrum with deconvoluted contributions labelled.

Table S6. Summary of the fittings of the deconvolutions of the C1s spectra extracted with CasaXPS software.

Sample	Peak	Position (eV)	FWHM	Area	Lineshape
Ti-MOF pristine	C-C	284.80	1.32	26313.33	GL(30)
	C-N	286.25	1.32	7863.16	GL(30)
	C=O	288.71	1.32	6715.48	GL(30)
	C-O	287.04	1.32	4029.98	GL(30)
$[(Ti\text{-}MOF)_{0.75}(IG)_{0.25}]$	C-C	284.75	1.38	22943.24	GL(30)
	C-N	286.19	1.38	7263.07	GL(30)
	C=O	289.09	1.38	7239.79	GL(30)
	C-O	287.50	1.38	221.39	GL(30)
$[(Ti\text{-}MOF)_{0.50}(IG)_{0.50}]$	C-C	284.72	1.38	12706.37	GL(30)
	C-N	286.13	1.38	6853.18	GL(30)
	C=O	288.94	1.38	3224.17	GL(30)
	C-O	287.55	1.38	439.66	GL(30)
$[(Ti\text{-}MOF)_{0.25}(IG)_{0.75}]$	C-C	284.74	1.36	8426.04	GL(30)
	C-N	286.00	1.36	10564.48	GL(30)
	C=O	288.82	1.36	1499.26	GL(30)
	C-O	287.52	1.36	1146.33	GL(30)

Table S7. Summary of the fittings of the deconvolutions of the O1s spectra extracted with CasaXPS software.

Sample	Peak	Position (eV)	FWHM	Area	Lineshape
Ti-MOF pristine	Ti-O	530.24	1.24	19173.78	LA(1.53,243)
	C=O/O-H	531.92	1.71	49665.28	LA(1.53,243)
$[(Ti\text{-}MOF)_{0.75}(IG)_{0.25}]$	C=O/O-H	531.84	1.83	47728.16	LA(1.53,243)
	P-O	533.42	1.83	31697.82	LA(1.53,243)
$[(Ti\text{-}MOF)_{0.50}(IG)_{0.50}]$	C=O/O-H	531.57	1.78	60035.73	LA(1.53,243)
	P-O	533.04	1.78	41546.47	LA(1.53,243)
$[(Ti\text{-}MOF)_{0.25}(IG)_{0.75}]$	C=O/O-H	532.91	1.74	105711.29	LA(1.53,243)
	P-O	531.48	1.74	118365.71	LA(1.53,243)

Table S8. Summary of the fittings of the deconvolutions of the Ti2p spectra extracted with CasaXPS software.

Sample	Peak	Position (eV)	FWHM	Area	Lineshape
Ti-MOF pristine	Ti $2p_{3/2}$	464.56	2.27	13055.75	LA(1.53,243)
	Ti $2p_{1/2}$	458.86	1.35	24691.57	LA(1.53,243)
$[(Ti\text{-}MOF)_{0.75}(IG)_{0.25}]$	Ti^{4+} $2p_{3/2}$	458.98	1.35	3519.62	LA(1.53,243)
	Ti^{4+} $2p_{1/2}$	464.68	2.27	1861.01	LA(1.53,243)
	$Ti^{\delta+}$ $2p_{3/2}$	459.90	1.37	2283.7	LA(1.53,243)
	$Ti^{\delta+}$ $2p_{1/2}$	465.55	2.43	1455.45	LA(1.53,243)
$[(Ti\text{-}MOF)_{0.50}(IG)_{0.50}]$	Ti^{4+} $2p_{3/2}$	458.52	1.35	775.53	LA(1.53,243)
	Ti^{4+} $2p_{1/2}$	464.22	2.27	410.06	LA(1.53,243)
	$Ti^{\delta+}$ $2p_{3/2}$	460.06	1.38	5410.78	LA(1.53,243)
	$Ti^{\delta+}$ $2p_{1/2}$	465.83	2.11	2771.9	LA(1.53,243)
$[(Ti\text{-}MOF)_{0.25}(IG)_{0.75}]$	Ti^{4+} $2p_{3/2}$	458.65	1.35	1022.53	LA(1.53,243)
	Ti^{4+} $2p_{1/2}$	464.15	2.27	613.52	LA(1.53,243)
	$Ti^{\delta+}$ $2p_{3/2}$	460.23	1.27	8541.72	LA(1.53,243)
	$Ti^{\delta+}$ $2p_{1/2}$	466.02	2.2	4907.01	LA(1.53,243)

11. Gas sorption

11.1 Nitrogen isotherms

Table S9. Data collected from N_2 isotherms at 77K, BET and t-plot analysis. BET surface area for the reported pristine Ti-MOF is 1469 m^2/g.[2]

Sample	Heat treatment	Surface area (BET) (m^2/g)
Ti-MOF pristine	110 ºC for 2 hours under vacuum	747
[$(Ti\text{-}MOF)_{0.75}(IG)_{0.25}$]	110 ºC for 2 hours under vacuum	420
[$(Ti\text{-}MOF)_{0.50}(IG)_{0.50}$]	110 ºC for 2 hours under vacuum	168
[$(Ti\text{-}MOF)_{0.25}(IG)_{0.75}$]	110 ºC for 2 hours under vacuum	N/A
[$(Ti\text{-}MOF)_{0.15}(IG)_{0.85}$]	110 ºC for 2 hours under vacuum	N/A
IG	110 ºC for 2 hours under vacuum	N/A

.

11.2 Carbon dioxide isotherms

Table S10. STP values of carbon dioxide adsorption isotherms.

**Sample**	**CO_2 STP (cm^3/g)**	
**Temperature**	**273 K**	**283 K**
**$70P_2O_5$-20%Na_2O-10%Na_2SO_4**	3.54	1.67
**$[(Ti\text{-}MOF)_{0.15}(IG)_{0.85}]$**	4.97	2.48
**$[(Ti\text{-}MOF)_{0.25}(IG)_{0.75}]$**	6.22	3.02
**$[(Ti\text{-}MOF)_{0.50}(IG)_{0.50}]$**	25.18	37.88
**$[(Ti\text{-}MOF)_{0.75}(IG)_{0.25}]$**	51.15	48.13
**Ti-MOF**	75.05	56.69

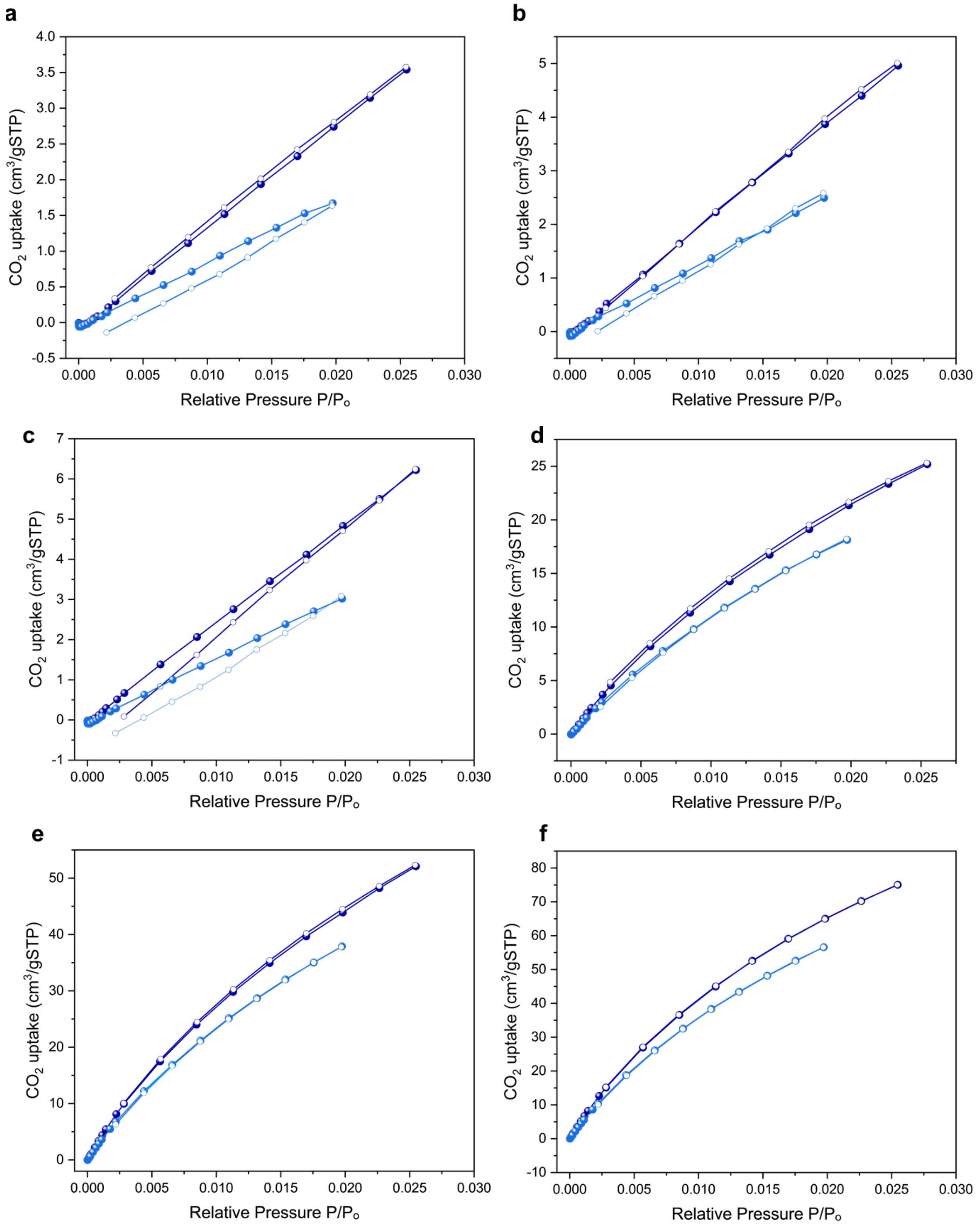

Figure S61. CO_2 adsorption/desorption isotherms collected at 273 K (navy) and 283 K (cyan) of the inorganic glass (**a**), compositional series of $[(Ti\text{-}MOF)_x(IG)_{1-x}]$ composites and the pristine Ti-MOF (**f**). **b**. $[(Ti\text{-}MOF)_{0.15}(IG)_{0.85}]$; **c**. $[(Ti\text{-}MOF)_{0.25}(IG)_{0.75}]$; **d**. $[(Ti\text{-}MOF)_{0.50}(IG)_{0.50}]$; **e**. $[(Ti\text{-}MOF)_{0.75}(IG)_{0.25}]$.

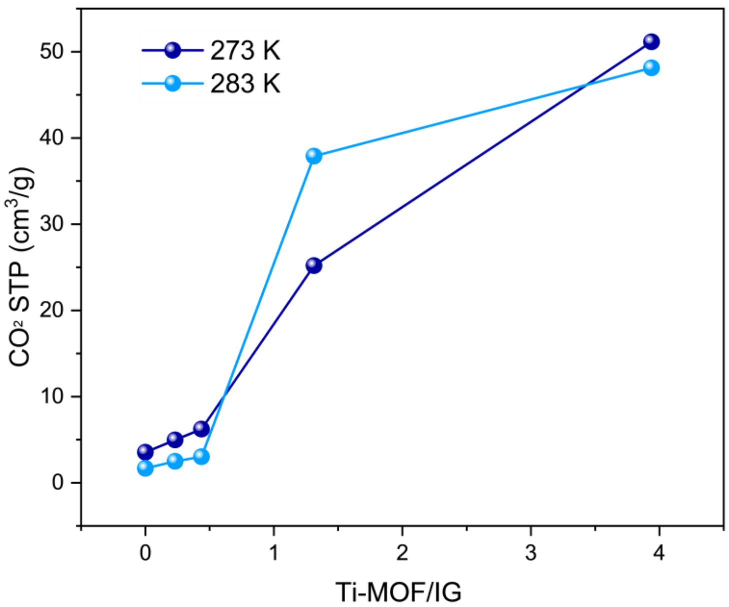

Figure S62. Ti-MOF/IG molar ratio vs the CO_2 uptake values.

11.3 Heat of adsorption

Isosteric heat of adsorption is a parameter that relates the strength of the attractive force between the CO_2 and the material. This parameter often appears within literature indistinctly as isosteric heat of adsorption (Q_{st}) or as enthalpy of adsorption (ΔH_{ads}), and they can be calculated through the use of the Clausius–Clapeyron equation using the Langmuir model (**eq 1**).[3]

$$\Delta H_{ads}(n) = -R \cdot ln\left(\frac{p_2}{p_1}\right)\frac{T_1 \cdot T_2}{(T_2 - T_1)} \qquad \text{eq 1}$$

where n is the amount adsorbed (the loading) in mmol g^{-1}, p the pressure in kPa, a is the maximal loading in mmol g^{-1}.

Regarding these composites possess domains, the isotherms were fitted using Sips isotherm model (extension of Langmuir model that accounts for heterogeneity in the surface) (**eq 2**) with the adsorbed amount Q_e at coresponding partial pressures P (**Figure S60**).[4]

$$Qe = \frac{Q_{ms} \cdot a_S \cdot P^b}{1 + a_S \cdot P^b}, \qquad \text{eq 2}$$

where Q_{ms}, a_s and b are isotherm constants. Q_{ms} represents maximum adsorption capacity of the adsorbent, a_s Sips equilibrium constant *and b* heterogeneity factor. Fitted parameters are given in **Table S8**.

Table S11. Fitting parameters of Sips model for CO_2 isotherms measured at specified temperatures.

Sample	Isotherms	Q_{ms}	a_s	b	R^2
**Glass4**	273K	0.61 ± 0.14	0.40 ± 0.12	1.26 ± 0.05	0.99939
	283K	0.21 ± 0.03	0.64± 0.16	1.36 ± 0.06	0.99926
**15% composite**	273K	1.08 ± 0.20	0.29 ± 0.07	1.16 ± 0.03	0.99975
	283K	0.88 ± 0.57	0.16 ± 0.12	1.11 ± 0.06	0.99917
**25% composite**	273K	2.42 ± 0.99	0.15 ± 0.07	1.09 ± 0.03	0.99972
	283K	0.36 ± 0.12	0.69 ± 0.37	1.34 ± 0.13	0.99404
**50% composite**	273K	3.26 ± 0.14	0.59 ± 0.04	0.96 ± 0.01	0.9999
	283K	2.36 ± 0.14	0.59 ± 0.05	1.01 ± 0.01	0.99986
**75% composite**	273K	7.04 ± 0.31	0.55 ± 0.04	0.91 ± 0.01	0.99992
	283K	5.03 ± 0.22	0.56 ± 0.04	0.96 ± 0.01	0.99993
**100% MOF**	273K	7.96 ± 0.12	0.81 ± 0.02	0.941 ± 0.005	0.99998
	283K	6.74 ± 0.14	0.67 ± 0.02	0.959 ± 0.006	0.99997

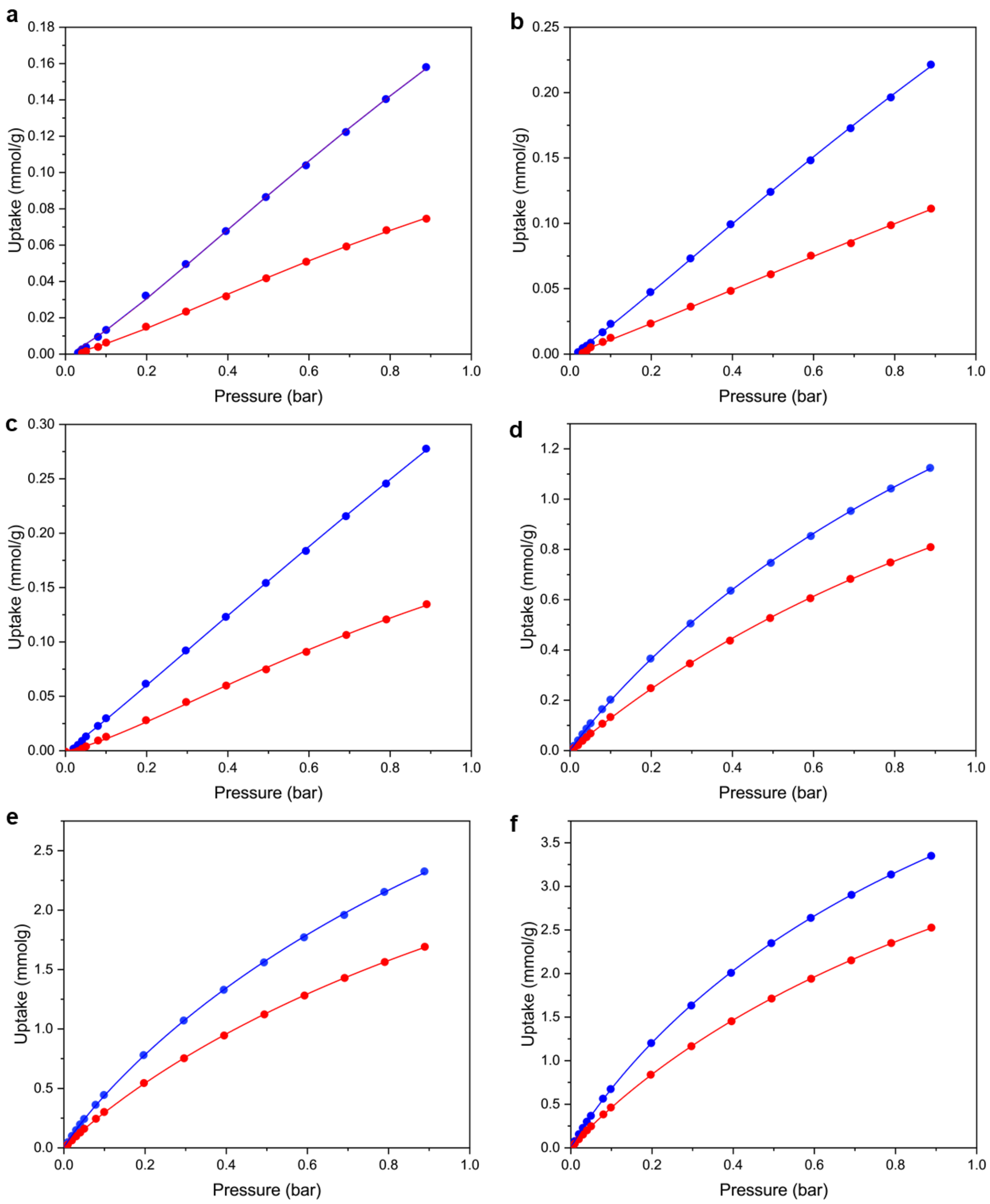

Figure S63. Fittings of the CO_2 isotherms (273 K in blue, 283 K in red) using the Sips model. **a**. Inorganic glass. **b**. [(Ti-MOF)$_{0.15}$(IG)$_{0.85}$]; **c**. [(Ti-MOF)$_{0.25}$(IG)$_{0.75}$]; **d**. [(Ti-MOF)$_{0.50}$(IG)$_{0.50}$]; **e**. [(Ti-MOF)$_{0.75}$(IG)$_{0.25}$]. **f**. Pristine Ti-MOF.

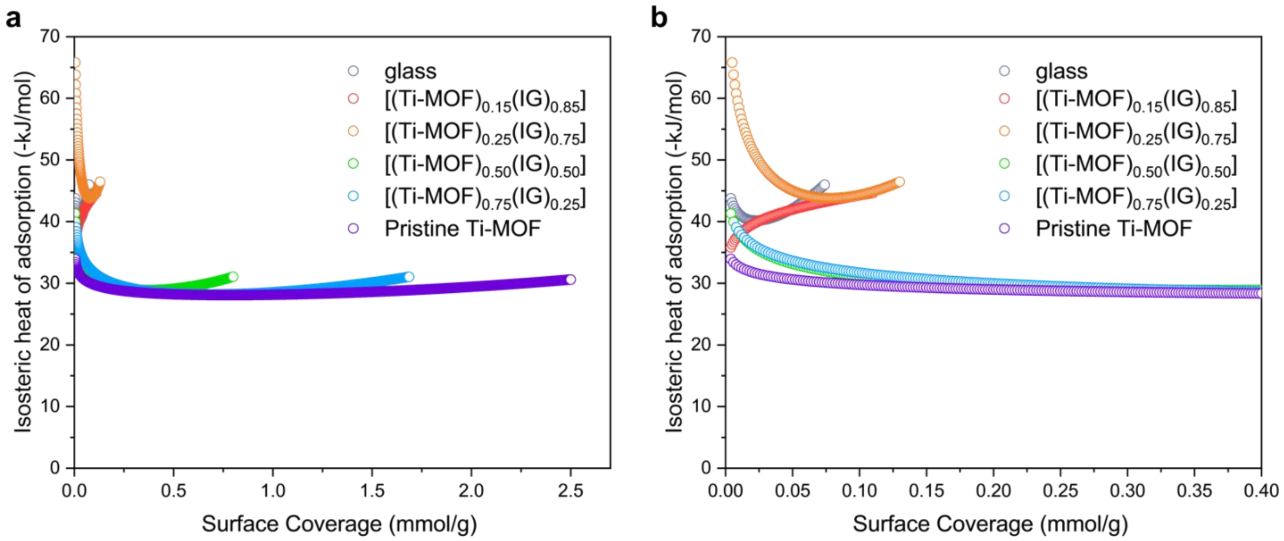

Figure S64. Isosteric heat of adsorption for the composite series and the pristine Ti-MOF and inorganic glass materials. a. Isosteric heat of adsorption at the whole range of the surface coverage. b. Zoom-in of the region between 0 and 0.4 mmol/g of the surface coverage.

12. Photocatalytic activity

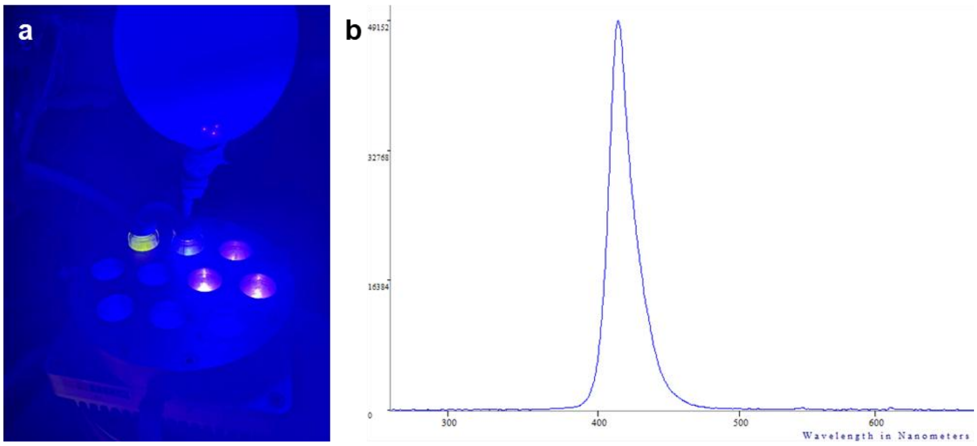

Figure S65. a. Picture of the photo reactor. **b**. UV-VIS spectrum of the region irradiated with the LEDs.

Table S12. Comparative study of the catalytic properties of the pristine Ti-MOF and pelletised Ti-MOF in the oxidative coupling of benzylamine.[a]

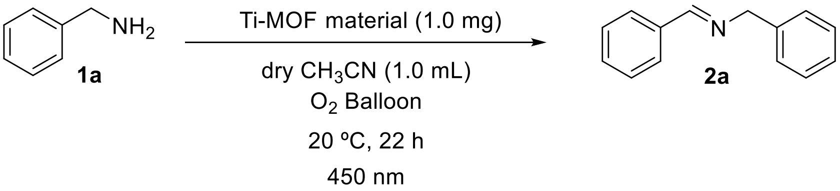

Entry	Material	Conversion into 2a (%)[b]
1	Pristine Ti-MOF	100
2	Pelletized Ti-MOF	94

[a] Reaction conditions: A mixture of **1a** (0.1 mmol), 1.0 mg of the corresponding Ti-MOF material in 1 mL of dry CH_3CN under oxygen atmosphere was irradiated at 450 nm for 22 h. [b] Conversions determined by ^1H NMR of the crude mixture.

Table S13. Comparison of the catalytic results with other conditions and other MIL-125-NH_2 composites.

Material	CH_3CN amount	Substrate amounts	Amount of Catalyst	Time	Yield (%)	Reference
MIL-125-NH_2	2 mL	0.1 mmol	5 mg	12 h	73%	6
$Pd_{0.5}$/ MIL-125-NH_2 Nanosheets	2 mL	0.1 mmol	5 mg	12 h	99.2	7
$Pd_{0.5}$/ MIL-125-NH_2	2 mL	0.1 mmol	5 mg	12 h	80.5	7
MIL-125-NH_2 Nanosheets	2 mL	0.1 mmol	5 mg	12 h	82.1	7
MIL-125-NH_2	1 mL	0.1 mmol	1 mg	48 h	95	This work
$[(Ti\text{-}MOF)_{0.75}(IG)_{0.25}]$	1 mL	0.1 mmol	0.5 mg<br>1 mg<br>1.5 mg	48 h	94<br>87<br>78	This work
$[(Ti\text{-}MOF)_{0.50}(IG)_{0.50}]$	1 mL	0.1 mmol	1 mg	48 h	58	This work
$[(Ti\text{-}MOF)_{0.25}(IG)_{0.75}]$	1 mL	0.1 mmol	1 mg	48 h	23	This work

12.1 Photocatalytic oxidative coupling of benzylamines. Optimisation of the reaction conditions

Table S14. Evaluation of solvents in the oxidative coupling of benzylamine.[a]

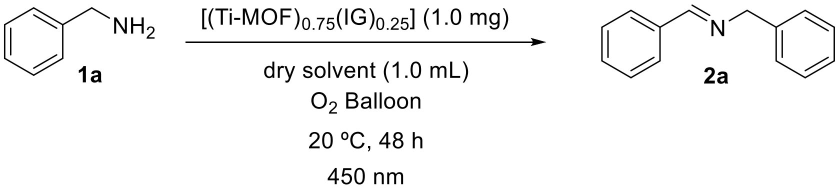

Entry	Solvent	Conversion into 2a (%)[b]
1	CH_3CN	85
2	DMF	85
3	CH_2Cl_2	79

[a] Reaction conditions: A mixture of **1a** (0.1 mmol), 1.0 mg of [(Ti-MOF)$_{0.75}$(IG)$_{0.25}$] in 1 mL of dry solvent was irradiated at 450 nm for 48 h. [b] Conversions determined by ^{1}H NMR of the crude mixture.

Table S15. Study of the effect of the amount of catalyst, [(Ti-MOF)$_{0.75}$(IG)$_{0.25}$] in the oxidative coupling of benzylamine.[a]

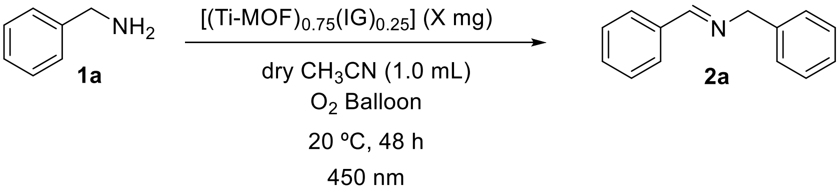

Entry	Amount of [(Ti-MOF)$_{0.75}$(IG)$_{0.25}$] (mg)	Conversion into 2a (%)[b]
1	1.5	78
2	1.0	85
3	0.5	94

[a] Reaction conditions: A mixture of **1a** (0.1 mmol), the corresponding amount of [(Ti-MOF)$_{0.75}$(IG)$_{0.25}$] in 1 mL of dry CH_3CN was irradiated at 450 nm for 48 h. [b] Conversions determined by ^{1}H NMR of the crude mixture.

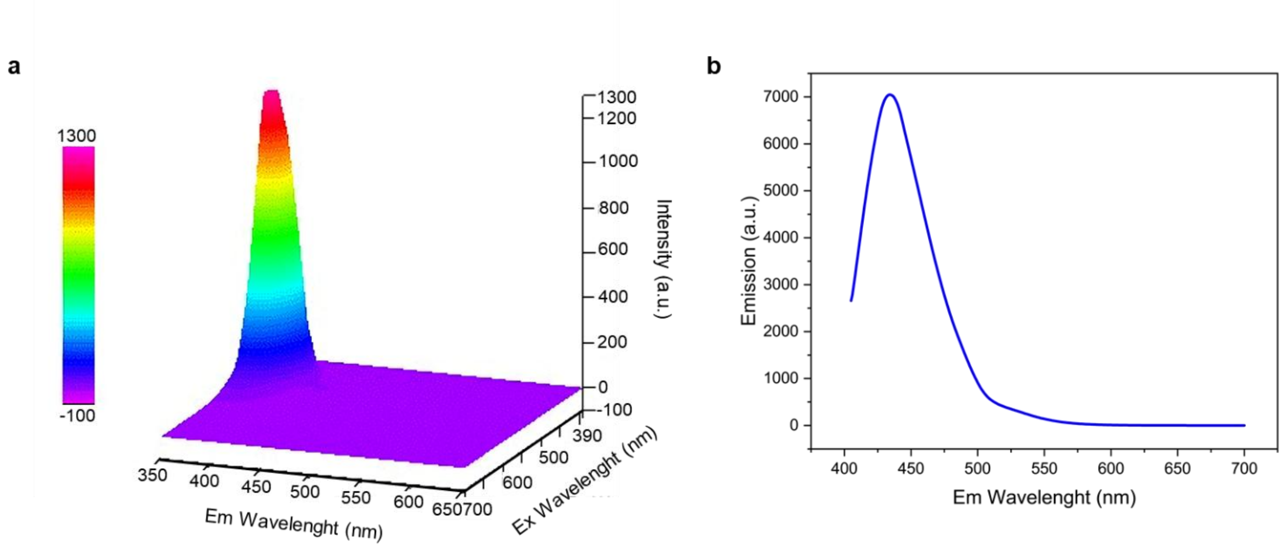

Figure S66. **a**. 2D spectra (emission vs excitation) of the [(Ti-MOF)$_{0.75}$(IG)$_{0.25}$] suspension. **b**. Emission spectra excited at 375 nm of the [(Ti-MOF)$_{0.75}$(IG)$_{0.25}$] suspension.

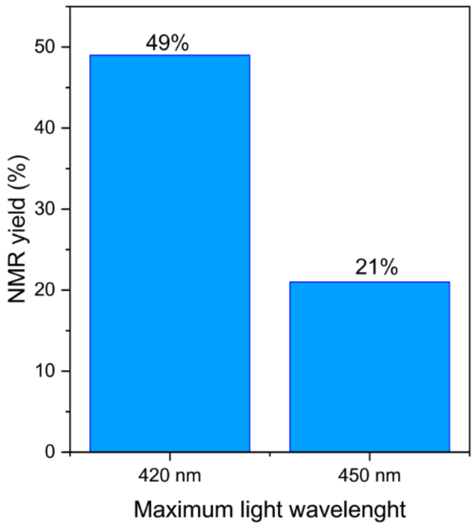

Figure S67. Catalytic study of the of the wavelength. Reaction conditions: A mixture of **1a** (0.1 mmol), 0.5 mg of [(Ti-MOF)$_{0.75}$(IG)$_{0.25}$] in 1 mL of dry CH_3CN was irradiated for 48 h.

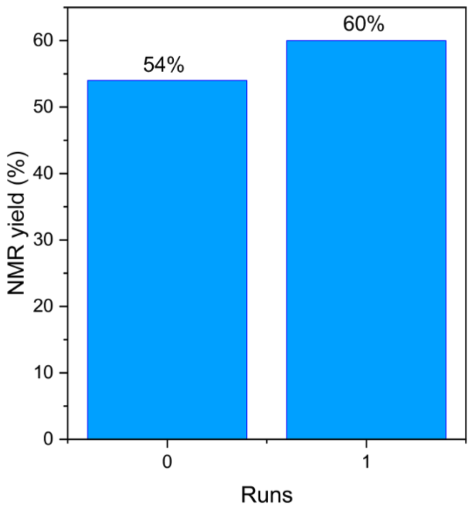

Figure S68. Recyclability of the composite. Reaction conditions: A mixture of **1a** (0.1 mmol), 0.5 mg of [(Ti-MOF)$_{0.75}$(IG)$_{0.25}$] in 1 mL of dry CH_3CN was irradiated at 420 nm for 48 h. Then, the reaction mixture was filtered and washed with acetonitrile and dichloromethane, and dried. Next, the composite was collected in a vial for being used in the next cycle by addition of a new batch of **1a** and solvent for undergoing the general procedure of the oxidative coupling.

12.2 General procedure for the oxidative coupling of amines

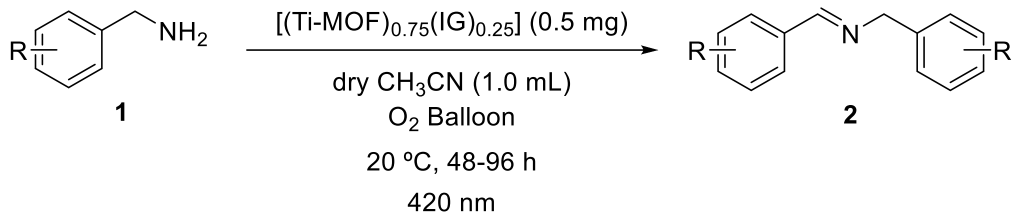

In a 10.0 mL vial charged with a stirring bar, the corresponding benzylamine **1a-h** (0.1 mmol), $[(Ti\text{-}MOF)_{0.75}(IG)_{0.25}]$ (0.5 mg) and 1.0 mL of dry acetonitrile were added. Then, the vial was sealed with a PTFE/rubber septum and a O_2 balloon was added. The reaction mixture was stirred under 420 nm irradiation at 20.0 ºC for 48-96 h to obtain the desired compound.

Due to the instability of the imine towards purification in silica, the crude reactions were analysed by ^{1}H NMR. For that, 1,3,5-trimethoxybenzene (2.8 mg, 0.016 mmol, 6.1 ppm, 3H; 3.8 ppm, 9H) as internal standard was added to the reaction crude; NMR tube was prepared by adding 0.5 mL of $CDCl_3$ to the reaction mixture, which was shaken.

(*E*)-*N*-Benzyl-1-phenylmethanimine (2a)

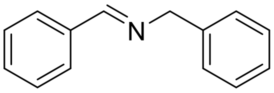

2a

Following the general procedure, phenylmethanamine (10.9 µL, 0.1 mmol) gave product **2a** (55% ^{1}H NMR yield) after 48 h.

^{1}H NMR (300 MHz, $CDCl_3$) δ 8.41 (s, 1H), 7.84 – 7.77 (m, 2H), 7.45 – 7.41 (m, 3H), 7.40 – 7.31 (m, 4H), 7.31 – 7.27 (m, 1H), 4.84 (s, 2H).

Spectra are consistent with those reported in the literature.[5]

(*E*)-*N*-(4-Methoxybenzyl)-1-(4-methoxyphenyl)methanimine (2b)

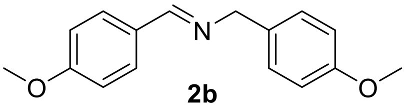

Following the general procedure, (4-methoxyphenyl)methanamine (13.7 mg, 0.1 mmol) gave product **2b** (80% ^{1}H NMR yield) after 96 h.

^{1}H NMR (300 MHz, $CDCl_3$) δ 8.30 (s, 1H), 7.78 – 7.67 (m, 2H), 7.31 – 7.20 (m, 2H), 6.99 – 6.83 (m, 4H), 4.73 (s, 2H), 3.81 (s, 3H), 3.78 (s, 3H).

Spectra are consistent with those reported in the literature.[5]

(*E*)-*N*-(4-Methylbenzyl)-1-(p-tolyl)methanimine (2c)

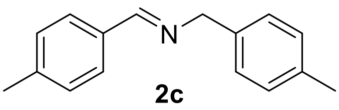

Following the general procedure, *p*-tolylmethanamine (12.1 mg, 0.1 mmol) gave product **2c** (70% ^{1}H NMR yield) after 96 h.

^{1}H NMR (300 MHz, $CDCl_3$) δ 8.35 (s, 1H), 7.67 (d, *J* = 8.1 Hz, 2H), 7.25 – 7.20 (m, 4H), 7.15 (d, *J* = 7.9 Hz, 2H), 4.78 (s, 2H), 2.39 (s, 3H), 2.34 (s, 3H).

Spectra are consistent with those reported in the literature.[5]

(*E*)-*N*-(4-(Trifluoromethyl)benzyl)-1-(4-(trifluoromethyl)phenyl)methanimine (2d)

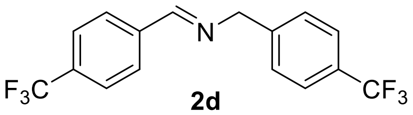

2d

Following the general procedure, (4-(trifluoromethyl)phenyl)methanamine (17.5 mg, 0.1 mmol) gave product **2d** (66% ^{1}H NMR yield) after 96 h.

^{1}H NMR (300 MHz, $CDCl_3$) δ 8.47 (d, *J* = 1.5 Hz, 1H), 7.92 (d, *J* = 8.0 Hz, 2H), 7.70 (d, *J* = 8.2 Hz, 2H), 7.63 (d, *J* = 8.0 Hz, 2H), 7.48 (d, *J* = 8.8 Hz, 2H), 4.91 (s, 2H).

Spectra are consistent with those reported in the literature.[5]

(*E*)-*N*-(3,5-Bis(trifluoromethyl)benzyl)-1-(3,5-bis(trifluoromethyl)phenyl)methanimine (2e)

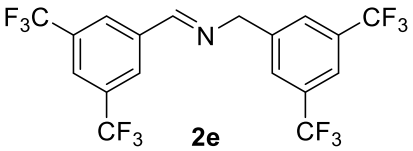

2e

Following the general procedure, (3,5-bis(trifluoromethyl)phenyl)methanamine (24.3 mg, 0.1 mmol) gave product **2e** (52% ^{1}H NMR yield) after 96 h.

^{1}H NMR (300 MHz, $CDCl_3$) δ 8.56 (s, 1H), 8.27 (s, 2H), 7.98 (s, 1H), 7.84 (s, 3H), 4.98 (s, 2H).

Spectra are consistent with those reported in the literature.[5]

(*E*)-4-(((4-Cyanobenzyl)imino)methyl)benzonitrile (2f)

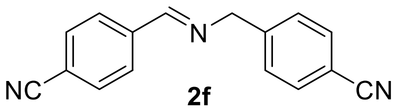

2f

Following the general procedure, 4-(aminomethyl)benzonitrile (13.2 mg, 0.1 mmol) gave product **2f** (67% ^{1}H NMR yield) after 96 h.

^{1}H NMR (300 MHz, $CDCl_3$): δ 8.47 (s, 1H), 7.91 (d, *J* = 8.4 Hz, 2H), 7.74 (d, *J* = 8.3 Hz, 2H), 7.66 (d, *J* = 8.3 Hz, 2H), 7.48 (d, *J* = 8.0 Hz, 2H), 4.92 (s, 2H).

Spectra are consistent with those reported in the literature.[5]

(E)-N-(4-Bromobenzyl)-1-(4-bromophenyl)methanimine (2g)

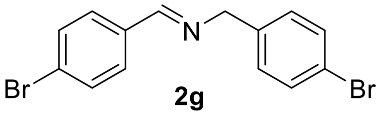

2g

Following the general procedure, (4-bromophenyl)methanamine (18.6 mg, 0.1 mmol) gave product **2g** (85% ^{1}H NMR yield) after 96 h.

^{1}H NMR (300 MHz, $CDCl_3$) δ 8.34 (s, 1H), 7.65 (d, *J* = 8.5 Hz, 2H), 7.56 (d, *J* = 8.5 Hz, 2H), 7.48 (d, *J* = 8.4 Hz, 2H), 7.22 (d, *J* = 8.3 Hz, 2H), 4.76 (s, 2H).

Spectra are consistent with those reported in the literature.[5]

<u>(*E*)-*N*-(4-Chlorobenzyl)-1-(4-chlorophenyl)methanimine</u> (2h)

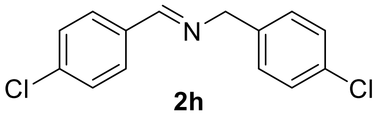

2h

Following the general procedure, (4-chlorophenyl)methanamine (14.2 mg, 0.1 mmol) gave product **2h** (61% ^{1}H NMR yield) after 96 h.

^{1}H NMR (300 MHz, $CDCl_3$) δ 8.35 (s, 1H), 7.72 (d, *J* = 8.5 Hz, 2H), 7.40 (d, *J* = 8.5 Hz, 2H), 7.35 – 7.23 (m, 3H), 4.78 (s, 2H).

Spectra are consistent with those reported in the literature.[5]

13. [1]H Nuclear magnetic resonance spectra

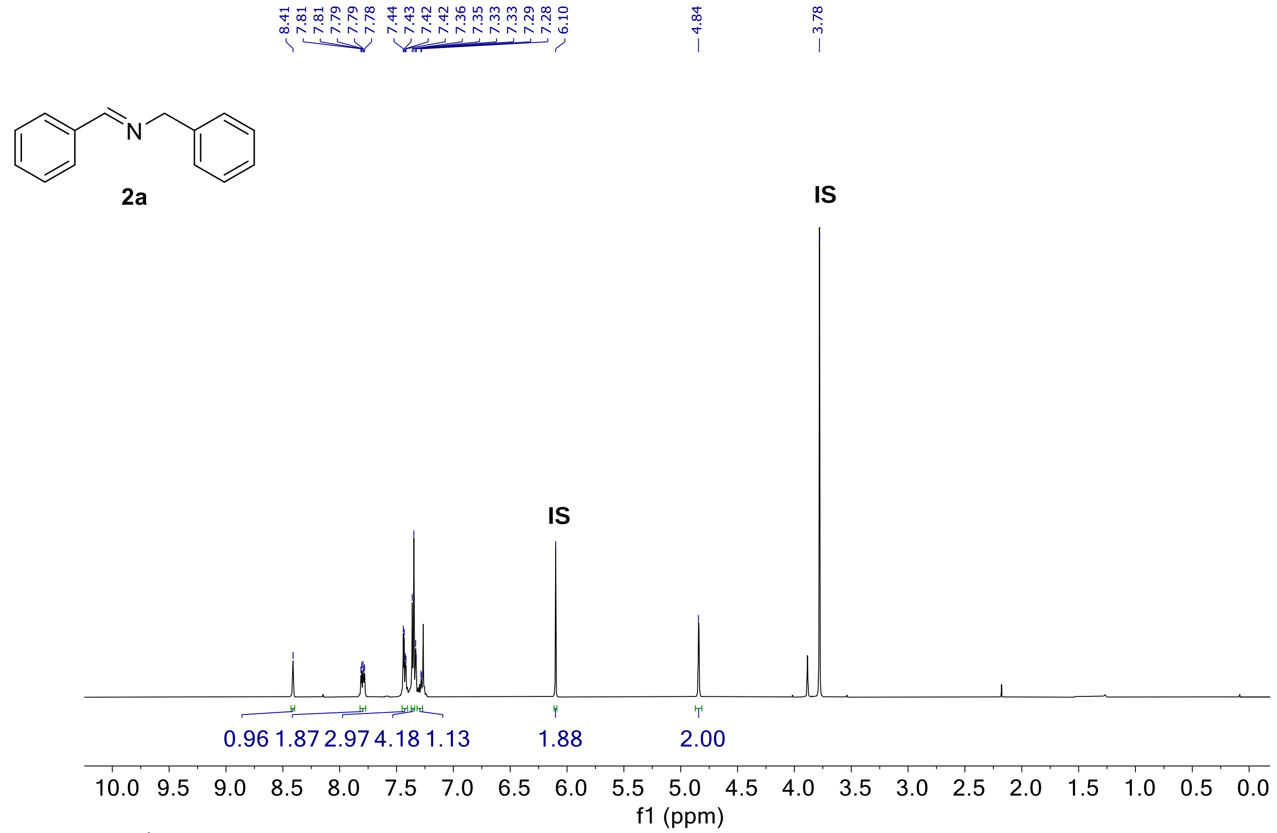

Figure S63. ^{1}H NMR spectrum of **2a** in $CDCl_3$. IS: internal standard.

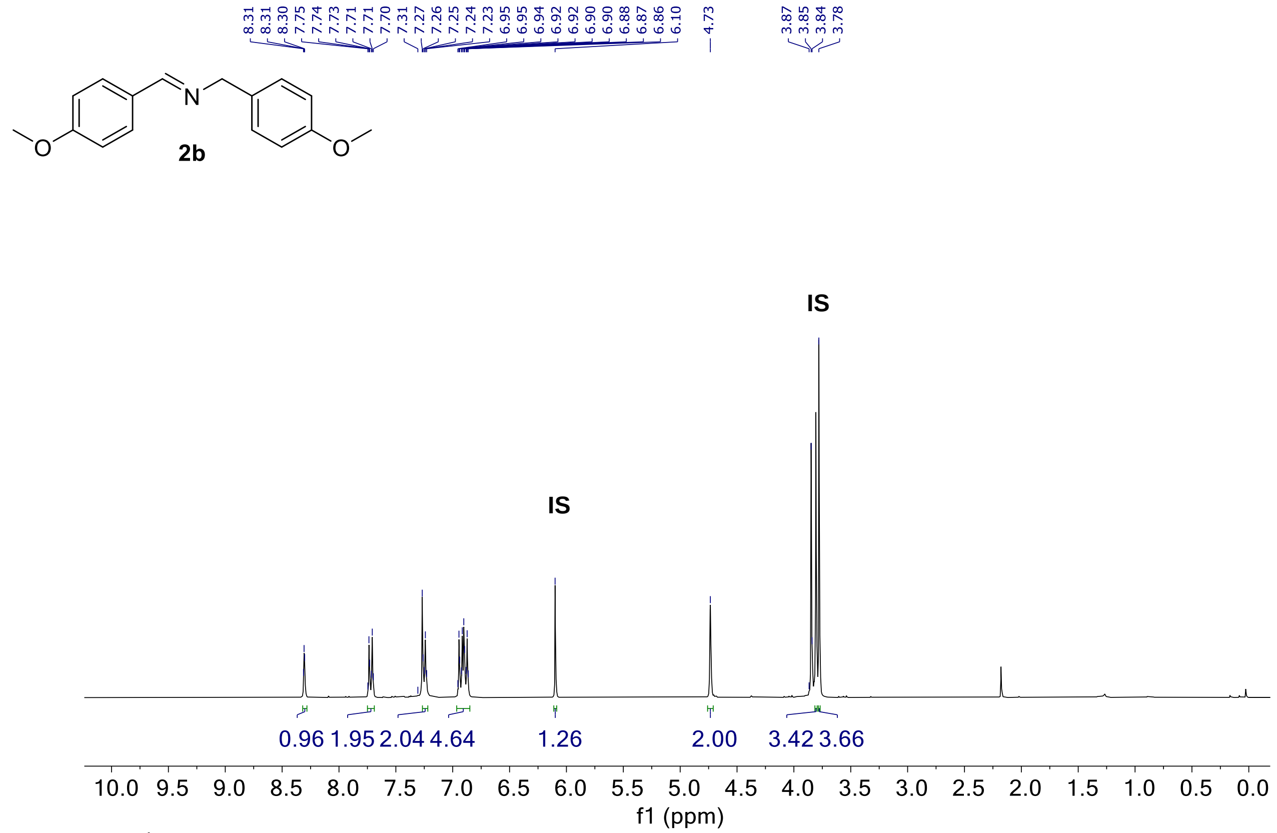

Figure S70. ^{1}H NMR spectrum of **2b** in $CDCl_3$. IS: internal standard.

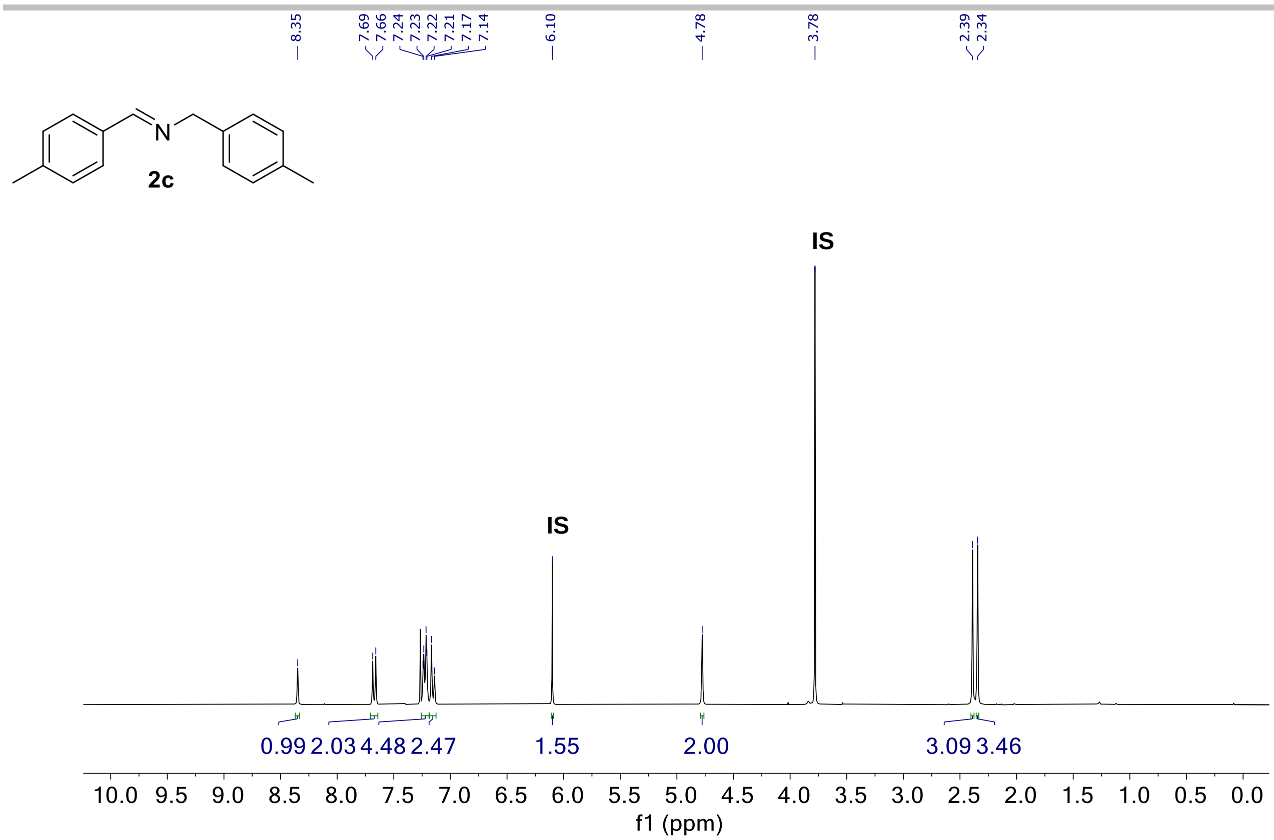

Figure S7164. ^{1}H NMR spectrum of **2c** in $CDCl_3$. IS: internal standard.

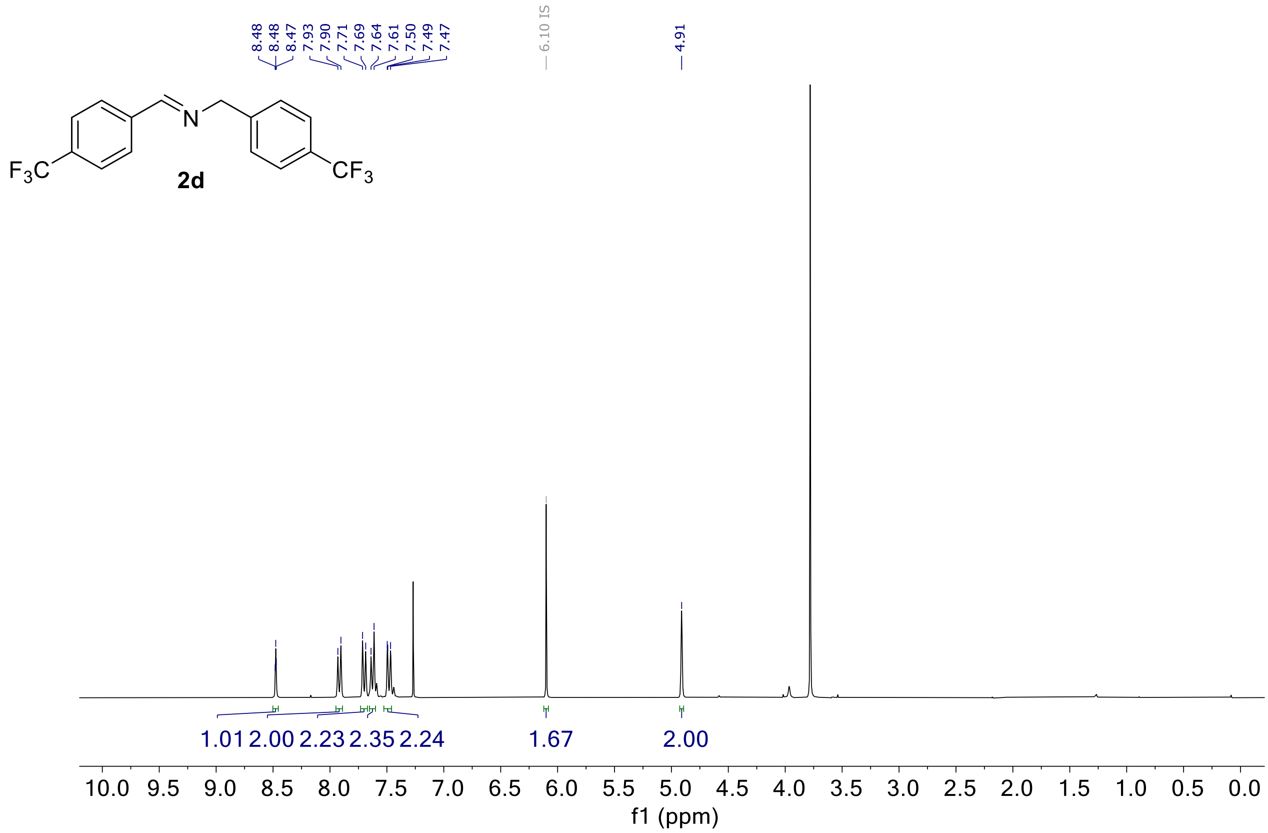

Figure S72. ^{1}H NMR spectrum of **2d** in $CDCl_3$. IS: internal standard.

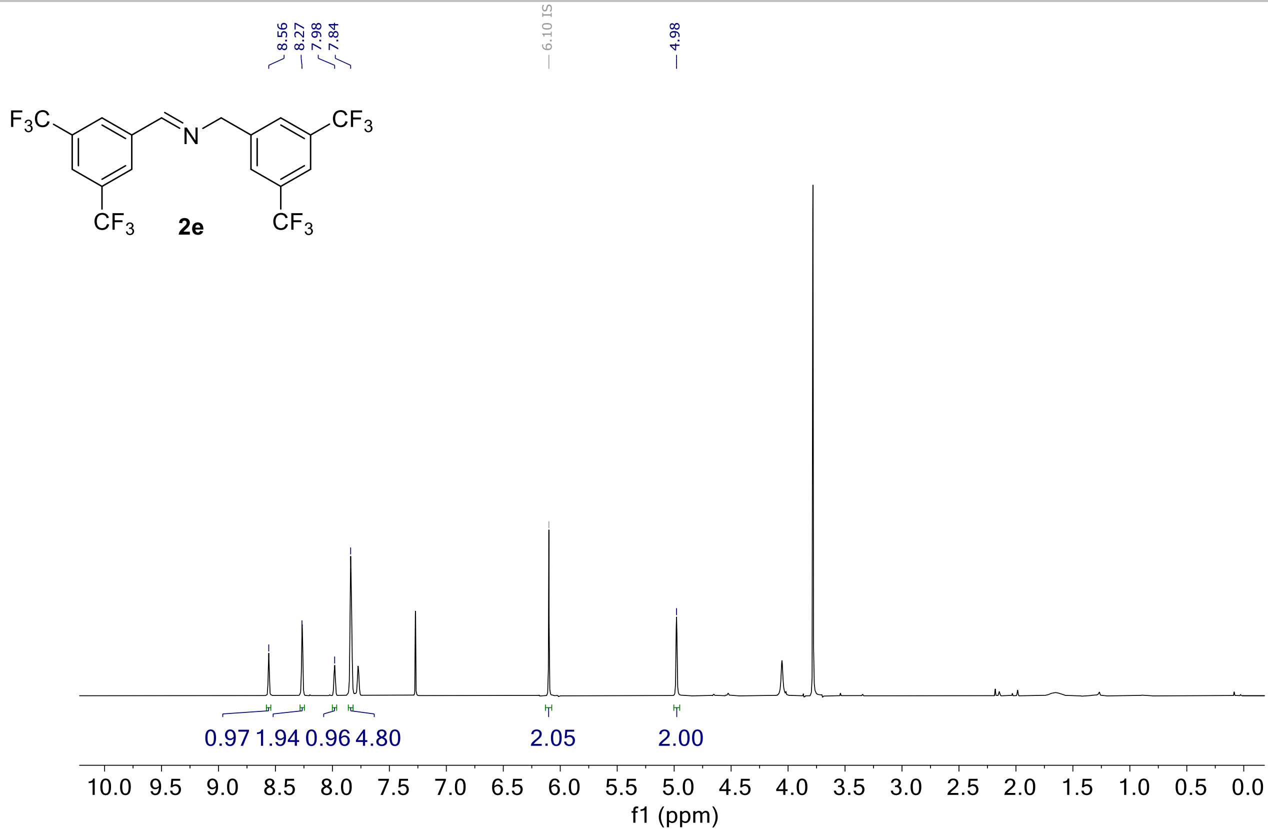

Figure S653. ^{1}H NMR spectrum of **2e** in $CDCl_3$. IS: internal standard.

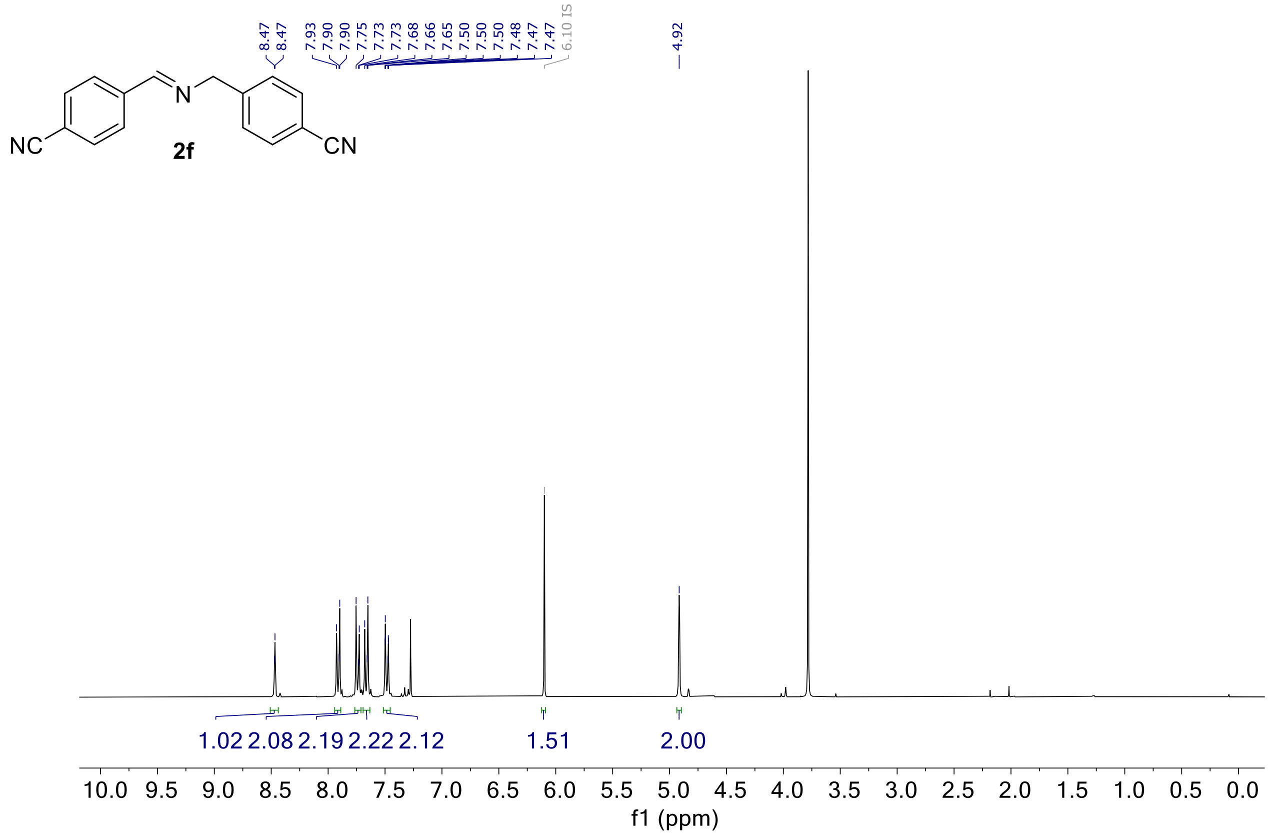

Figure S74. ^{1}H NMR spectrum of **2f** in $CDCl_3$. IS: internal standard.

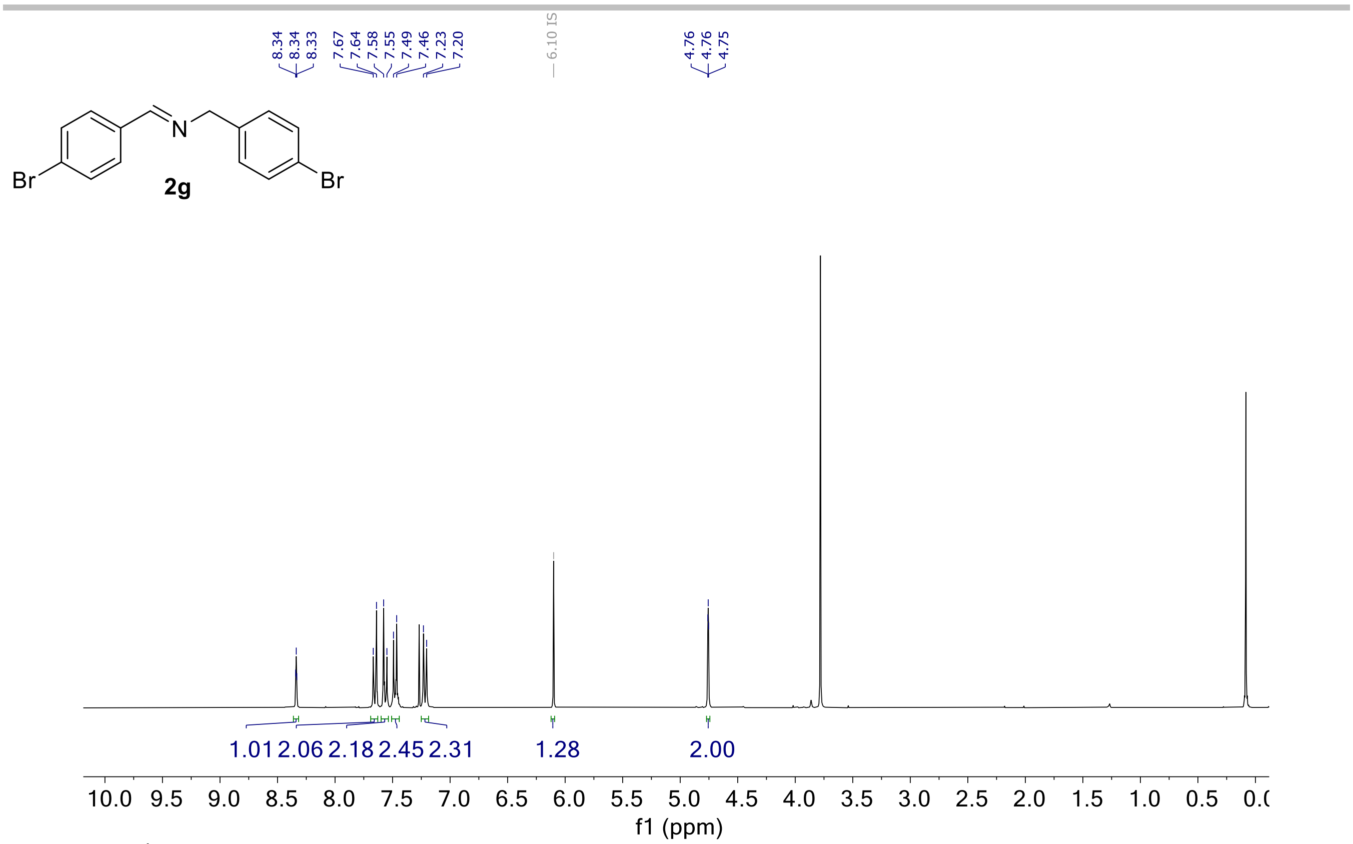

Figure S75. ^{1}H NMR spectrum of **2g** in $CDCl_3$. IS: internal standard.

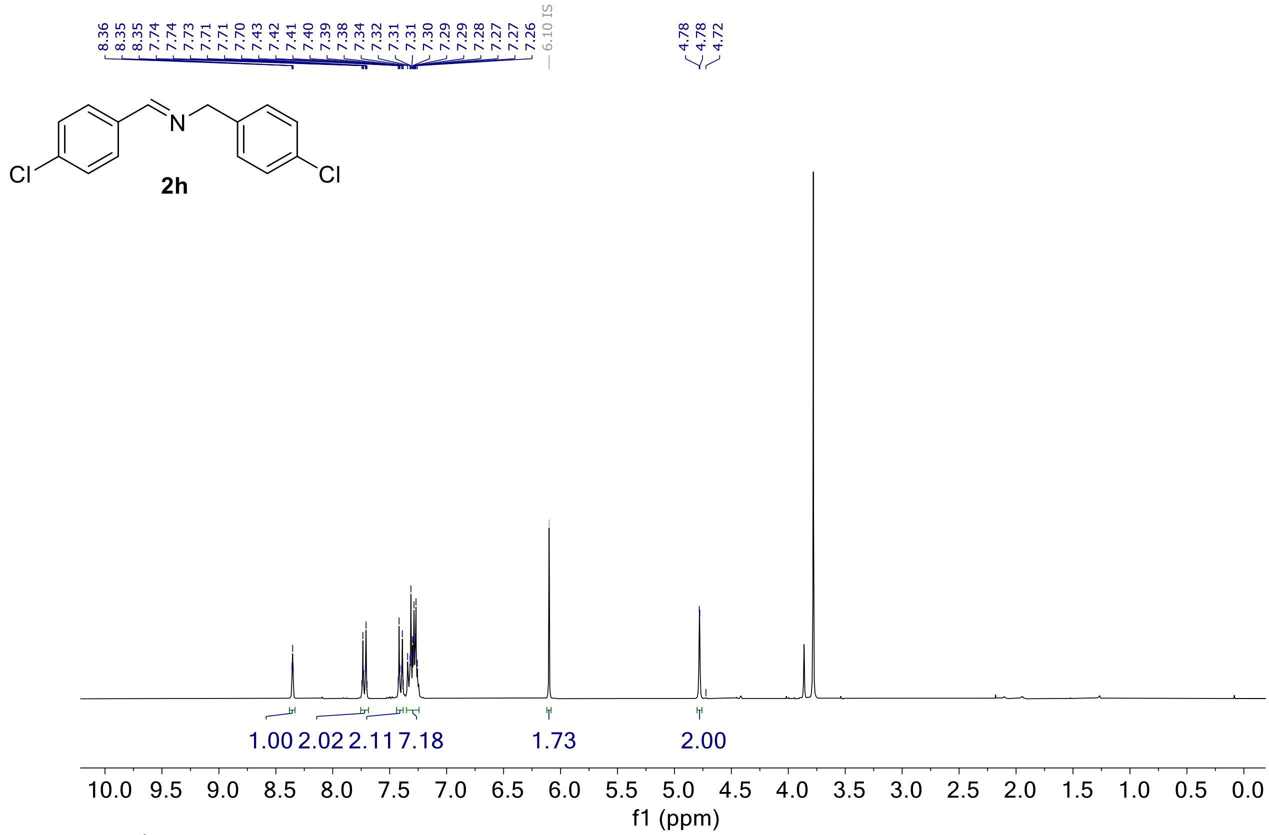

Figure S76. ^{1}H NMR spectrum of **2h** in $CDCl_3$. IS: internal standard.

14. Stability study

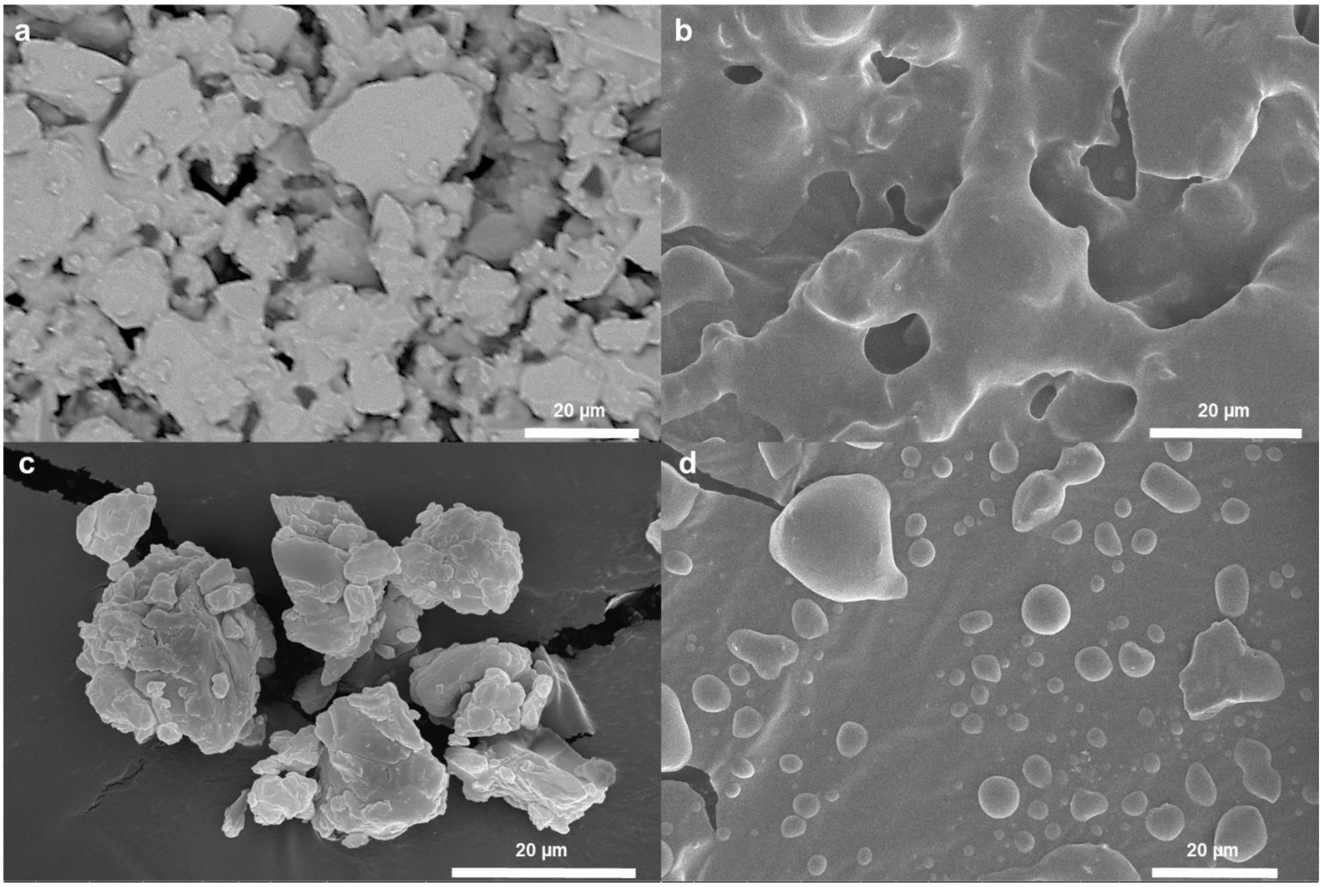

Figure S77. SEM images of **a**. the inorganic glass (IG) after manual grinding, **b**. After manual grinding and exposed to the air for two hours, **c**. after ball milling in air for 30 min at 30 Hz and **d**. after ball milling in air for 30 min at Hz and exposed to the air for two hours.

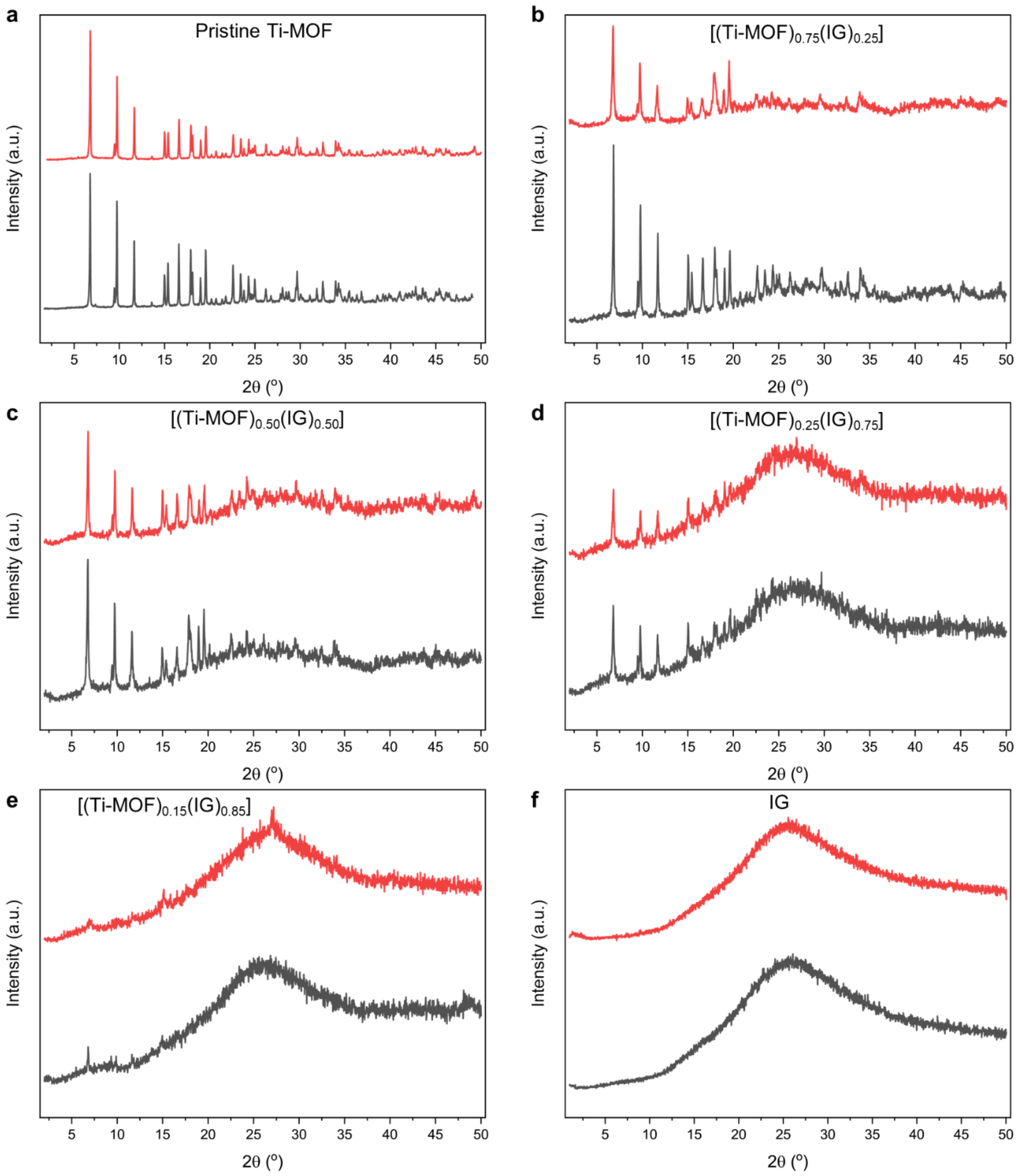

Figure S78. PXRD patterns of the materials without any exposure to the air (grey) and after exposure to the air for seven days (red) for a. pristine Ti-MOF, **b**. $[(Ti\text{-}MOF)_{0.75}(IG)_{0.25}]$ composite, **c**. $[(Ti\text{-}MOF)_{0.50}(IG)_{0.50}]$ composite, **d**. $[(Ti\text{-}MOF)_{0.25}(IG)_{0.75}]$ composite, **e**. $[(Ti\text{-}MOF)_{0.15}(IG)_{0.85}]$ composite and **f**. the inorganic glass.

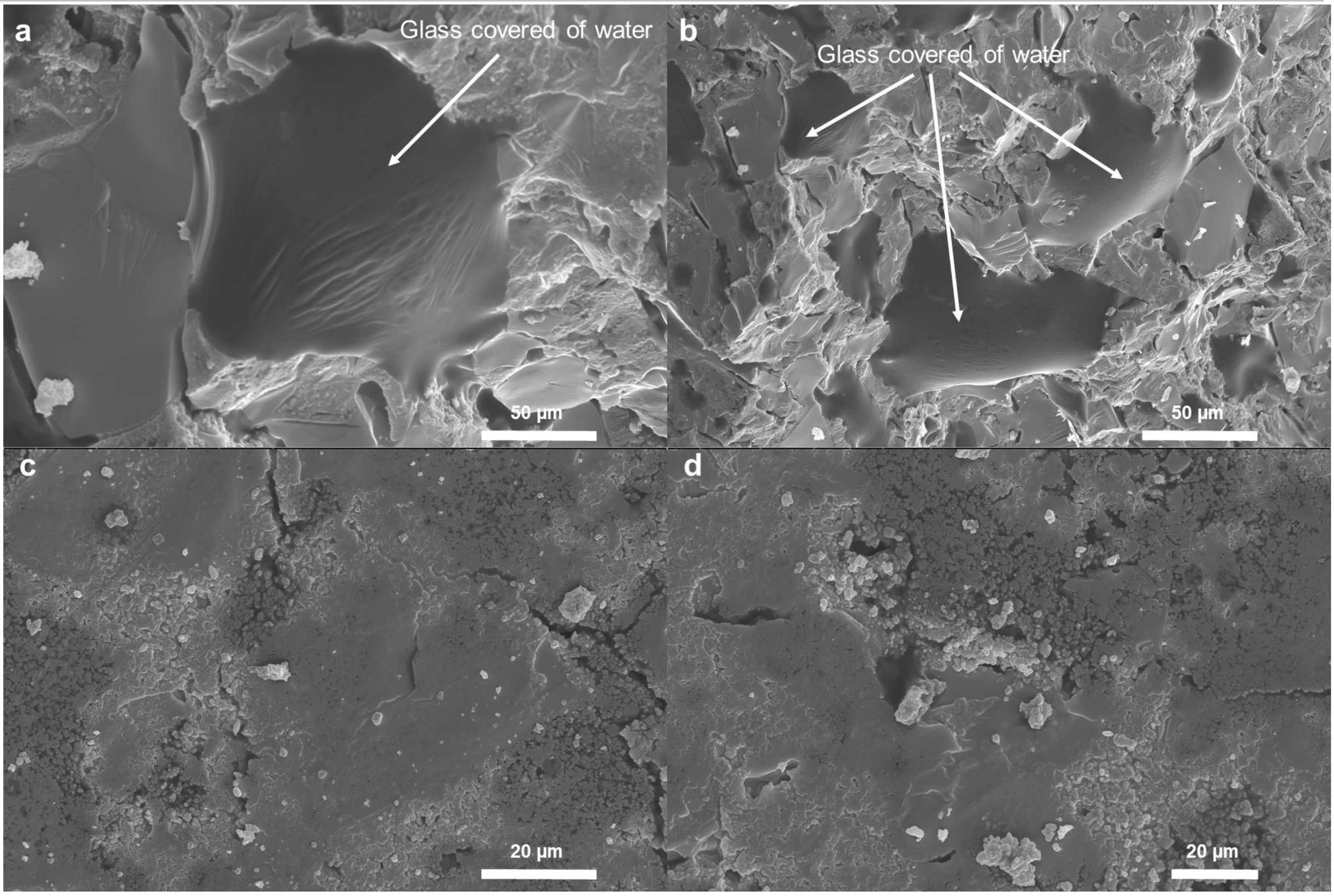

Figure S79. SEM images of **a**. [(Ti-MOF)$_{0.15}$(IG)$_{0.85}$] composite, **b**. [(Ti-MOF)$_{0.25}$(IG)$_{0.75}$] composite, **c**. [(Ti-MOF)$_{0.50}$(IG)$_{0.50}$] composite, **d**. [(Ti-MOF)$_{0.75}$(IG)$_{0.25}$] composite exposed to the air for seven days.

15. References

(1) Dan-Hardi, M.; Serre, C.; Frot, T.; Rozes, L.; Maurin, G.; Sanchez, C.; Férey, G. A New Photoactive Crystalline Highly Porous Titanium(IV) Dicarboxylate. *J. Am. Chem. Soc.* **2009**, *131* (31), 10857–10859.

(2) Kim, S.-N.; Kim, J.; Kim, H.-Y.; Cho, H.-Y.; Ahn, W.-S. Adsorption/Catalytic Properties of MIL-125 and NH2-MIL-125. *Catal. Today* **2013**, *204*, 85–93.

(3) Nuhnen, A.; Janiak, C. A Practical Guide to Calculate the Isosteric Heat/Enthalpy of Adsorption via Adsorption Isotherms in Metal–Organic Frameworks, MOFs. *Dalt. Trans.* **2020**, *49* (30), 10295–10307.

(4) Serafin, J.; Dziejarski, B. Application of Isotherms Models and Error Functions in Activated Carbon CO2 Sorption Processes. *Microporous Mesoporous Mater.* **2023**, *354*, 112513.

(5) Jiménez-Almarza, A.; López-Magano, A.; Mas-Ballesté, R.; Alemán, J. Tuning the Activity–Stability Balance of Photocatalytic Organic Materials for Oxidative Coupling Reactions. *ACS Appl. Mater. Interfaces* **2022**, *14* (14), 16258–16268.

(6) Sun, D.; Ye, L.; Li, Z. Visible-Light-Assisted Aerobic Photocatalytic Oxidation of Amines to Imines over NH2-MIL-125(Ti). *Appl. Catal. B Environ.* **2015**, *164*, 428–432.

(7) Wang, H.; Yu, J.; Wei, S.; Lin, M.; Song, Y.; Wu, L. Surface Coordination Enhanced Visible-Light Photocatalytic Coupling of Benzylamine to N-Benzylidene Benzylamine over the Pd/NH2-MIL-125(Ti) Nanosheets. *Chem. Eng. J.* **2022**, *441*, 136020.
